# Tuning intracellular immunity by Nodamura virus B2 protein enhances self-amplifying RNA activity

**DOI:** 10.1038/s41467-026-77816-2

**Published:** 2026-09-28

**Authors:** Raul Y. Sanchez-David, Josephine Nemegeer, Hoang Duy Le, Eva Žusinaite, Livia Spiga, Amanda Gonçalves, John S. Tregoning, Andres Merits, Pierre V. Maillard

**Affiliations:** 1 Centre for Immunobiology and Infection, Blizard Institute, Queen Mary University of London, London, UK; 2 Institute of Bioengineering, University of Tartu, Tartu, Estonia; 3 Department of Infectious Disease, South Kensington Campus, Imperial College London, London, UK; 4 Department of Biomedical Molecular Biology, Faculty of Science, Ghent University, Ghent, Belgium; 5 VIB BioImaging Core, VIB-UGhent Center for Inflammation Research, Technologiepark-Zwijnaarde, Ghent, Belgium; 6 Present Address: Thayer School of Engineering, Dartmouth College, Hanover, NH USA

**Keywords:** Pattern recognition receptors, Viral host response, RNA vaccines

## Abstract

Self-amplifying RNA (saRNA) offers a powerful approach for vaccine development, as its replicative nature within cells provides prolonged antigen expression together with self-adjuvanticity. However, its potency is often limited by innate immune responses, particularly those triggered by intracellular double-stranded RNA (dsRNA), highlighting the need for strategies that can boost saRNA efficacy without compromising its immunostimulatory properties. Here we show that the *cis*-expression of the Nodamura virus (NoV) B2 protein markedly enhances saRNA-driven transgene expression by alleviating key cell-intrinsic antiviral effectors. In stem cells, NoV B2 limits the accumulation of Dicer-generated viral small RNAs, consistent with suppression of antiviral RNA interference, while in somatic cells, it prevents protein kinase R (PKR)-driven translational shutdown. NoV B2 sequesters dsRNA at the cell periphery, shielding it from antiviral effectors without suppressing type I interferon induction or signalling, thereby preserving saRNA’s immunostimulatory properties, while enhancing its transgene expression potential. These findings reveal a strategy to overcome intracellular antiviral restriction of saRNA while preserving adjuvanticity, with implications for the design of more effective saRNA-based vaccines and therapeutics.

## Abstract (web summary)

This study shows that expressing the viral protein B2 in self-amplifying RNA overcomes innate antiviral defences, boosting transgene expression while preserving immune-stimulating properties.

## Introduction

Following the success of messenger RNA (mRNA)-based vaccines to tackle the global COVID-19 pandemic, there is tremendous interest in their further optimisation. Self-amplifying RNA (saRNA) holds great promise for efficient vaccine design since its ability to replicate within cells enables robust and prolonged antigen expression in vivo, thereby allowing lower doses to confer protective immunity compared to non-replicative mRNAs^1–3^.

SaRNAs are often derived from alphaviruses, a family which includes human pathogens such as chikungunya virus (CHIKV) and Venezuelan equine encephalitis (VEEV)^2,4^. The alphavirus genome is a ∼12 kilobases (kb) long RNA of positive (+) polarity and comprises two open reading frames (ORFs). The 5’ ORF encodes for a polyprotein, which after processing, results in four non-structural proteins (nsP1-4) that form the viral replication complex (vRC). The 3’ ORF is expressed from a subgenomic RNA (sgRNA) produced from an internal promoter located on the negative (-)-sense RNA replication intermediate. The sgRNA encodes for all the structural proteins, which together with newly synthesised genomes form new viral particles^4^.

In saRNA, all the structural genes are replaced by a gene of interest (GOI) thereby eliminating its capacity to produce viral particles while maintaining its ability to self-amplify^1,2^. However, saRNAs induce a strong innate immune response that enhances their adjuvanticity, yet also hampers their efficiency by activating cell-intrinsic innate antiviral mechanisms detrimental for saRNA stability, replication, transcription and/or translation. During self-amplification, the vRC generates double-stranded RNA (dsRNA) replication intermediates that trigger innate immune responses such as the interferon (IFN) system^5,6^. In vertebrates, cytosolic viral RNAs are sensed by the RIG-I-like receptors (RLRs) including RIG-I (retinoic acid-inducible gene I), MDA5 (melanoma differentiation factor 5) and LGP2 (laboratory of genetics and physiology 2), which trigger a signalling cascade via the adaptor protein MAVS (mitochondrial antiviral signalling protein)^5,6^. This leads to the activation of the transcription factors IRF3 and IRF7 (interferon regulatory factor 3 and 7) and NF-κB (nuclear factor kappa-light chain enhancer of activated B cells) resulting in the induction of type I IFN (IFN-I) and proinflammatory cytokines, respectively^5,6^. Both RIG-I and MDA5-mediated innate immune responses are modulated by LGP2^6–8^. Proinflammatory cytokines initiate inflammation in activated tissues, while IFN-I binds to IFNAR (IFN-α/β receptor) and signals through the JAK/STAT (Janus Kinase/signal transducer and activator of transcription) pathway to induce hundreds of interferon-stimulated genes (ISGs)^9^. These include cytokines, chemokines and co-stimulatory molecules that promote adaptive immunity^10^. The ability of saRNA to generate dsRNA confers strong intrinsic adjuvanticity and enhances immunogenicity compared to non-replicative mRNAs^11–13^. However, many ISGs have antiviral activities that can impair saRNA efficiency^9^. For instance, the ISG-encoded serine/threonine kinase PKR (protein kinase R) plays a central role in the antiviral effects mediated by IFN-I^14^. PKR is constitutively expressed at steady-state but is further upregulated by IFN-I. It becomes activated upon binding to dsRNA of viral or endogenous origin. Upon recognition of dsRNA, PKR phosphorylates the alpha subunit of eukaryotic translation initiation factor 2 (eIF2α), thus inhibiting both cellular and viral protein synthesis^14–16^. Supporting the negative impact of the IFN-I response on saRNA efficacy, saRNA-mediated protein expression increases when type I IFN signalling is inhibited or in MAVS-deficient mice unable to signal through the RLRs^13^. Similarly, co-expression of some viral IFN antagonists enhances saRNA immunogenicity^17^. In addition to the IFN system, cytosolic dsRNA also triggers a distinct antiviral pathway based on RNA interference (RNAi), which we hypothesised might similarly limit saRNA function.

The RNAi pathway acts as the main antiviral defence mechanism in plants and invertebrates, and recent work shows that it is also active in mammals^18,19^. In antiviral RNAi, the endoribonuclease Dicer cleaves viral dsRNAs into viral small interfering RNAs (vsiRNAs) of 21 to 23 nucleotides (nt) in length. These vsiRNAs are then loaded into the RNA-induced silencing complex (RISC) composed of Argonaute (Ago) proteins and mediate the sequence-specific cleavage of complementary target viral RNAs^18,19^. Mammalian antiviral RNAi was shown in embryonic and tissue stem cells, which naturally have an attenuated IFN response^20–22^ and express a specific isoform of Dicer called aviD (antiviral Dicer) with increased ability to cleave dsRNA in vitro and a heightened antiviral activity^23–25^. In differentiated cells, aviD is expressed at low levels and antiviral RNAi activity is further dampened by IFN-dependent mechanisms^26–29^.

Many viruses encode viral suppressors of RNAi (VSRs) that inhibit the RNAi pathway and are necessary for efficient virus multiplication and spread^18,30^. Among the best characterised VSRs are the B2 proteins of the *Nodaviridae* family, which includes flock house virus (FHV) and Nodamura virus (NoV)^18,30^. While FHV infects only insects^31,32^, NoV also causes lethality in suckling mice and pigs^33–35^. B2 proteins homodimerize to bind synthetic or viral dsRNAs and siRNAs and exert their VSR activity by sequestering them from the RNAi machinery^32,36–39^. Accordingly, NoV mutants lacking B2 or expressing a B2 dsRNA-binding-deficient version (NoV B2^R59Q^) accumulate to lower levels in both mouse embryonic stem cells (mESCs), somatic cells (e.g., mouse embryonic fibroblasts) and newborn mice^23,40,41^. These infections are accompanied by the accumulation of canonical vsiRNAs and, in cells, NoV mutants’ replication is rescued upon inactivation of the RNAi pathway. The ability of B2 to suppress antiviral RNAi in stem cells, and to a lesser extend in somatic cells where RNAi is dampened but not absent, prompted us to test whether its *cis*-expression from saRNA could enhance its efficacy in both cell types.

Here, we show that *cis*-expression of NoV B2 protein enhances saRNA-driven protein expression from CHIKV and VEEV-based saRNA vectors in both mESCs and various somatic cell lines. In mESCs, NoV B2 protects saRNA from Dicer-mediated cleavage, while in somatic cells, it prevents PKR-mediated translation block without affecting IFN induction and signalling. Notably, B2 expression limits the cytoplasmic re-localisation of the saRNA-derived dsRNA and is associated with its accumulation at the cell periphery.

## Results

### NoV B2 increases saRNA-mediated protein expression in mESCs

**Fig. 1:**
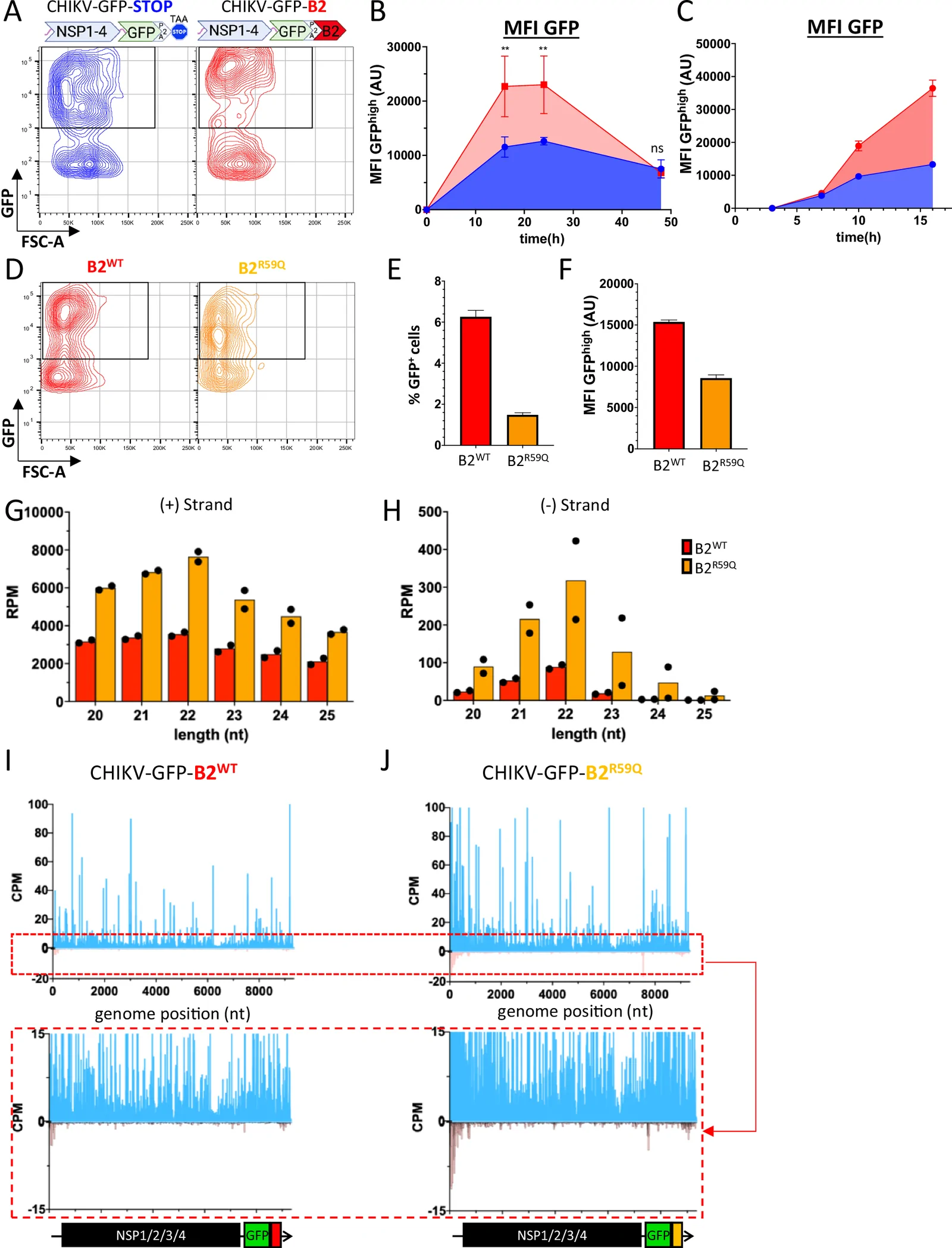
NoV B2 enhances CHIKV-based saRNA in mESCs and inhibits the accumulation of ∼22-nt viral small RNAs. **A** Representative contour plots of GFP-positive (GFP^+^) E14 mouse embryonic stem cells (mESCs) 24 h post-transfection with CHIKV-GFP-STOP (blue) or -B2^WT^ (red) saRNA. Gate indicates the GFP^high^ population selected for Mean Fluorescence Intensity (MFI) analysis in (**B**, **C**). Unless otherwise stated, 0.1 µg saRNA was used throughout the study. **B** GFP^high^ MFI by flow cytometry in E14 mESCs transfected with CHIKV-GFP-STOP (blue) or -B2^WT^ (red) at 16, 24, and 48 h post-transfection. Mean ± standard deviation (s.d.), *n* = 3 independent experiments. **C** GFP^high^ MFI as in (**B**) at earlier time points (3, 7, 10, and 16 h post-transfection). Mean ± s.d., *n* = 3 three technical replicates. Data representative of two independent experiments. **D** Representative contour plots of GFP^+^ E14 mESCs 24 h post-transfection with CHIKV-GFP-B2^WT^ (Red) or B2^R59Q^ mutant (orange). Gate indicates the GFP^high^ population used for MFI analysis in (**F**). **E**, **F** Percentage of GFP^+^ cells (**E**) and GFP^high^ MFI (**F**) after transfection with indicated saRNA. Mean ± s.d., *n* = 3 independent experiments. **G**, **H** Frequency distribution of small RNAs of indicated lengths mapping to the (**G**) (+) and (**H**) (−) strands of CHIKV-GFP-B2^WT^ (red) or -B2^R59Q^ (orange) vector genome. Each dot represents one biological replicate (*n* = 2). RPM: reads per million, normalised to total 15-35 nt reads. **I**, **J** Distribution of 21- and 23-nt reads along the (+) and (–) strands of CHIKV^ERR^ saRNA genome from small RNA deep sequencing of GFP^+^-sorted cells transfected with saRNA encoding (**I**) B2^WT^ or (**J**) B2^R59Q^. CPM, counts per million of total 15-35-nt reads mapping to the vector genome. Statistical analyses were performed using two-way ANOVA with Šídák’s multiple comparisons test (**B**). **P < 0.01. arb. units: arbitrary units. Exact *p* values and source data are provided as a Source Data file.

We engineered a CHIKV-based saRNA encoding nsP1-4 and green fluorescent protein (GFP) under the control of a subgenomic promoter. To reduce cytopathic effects and transcriptional shutoff, hallmarks of alphaviruses, we used a mutated version of nsP2 protein (ATL to ERR substitutions at positions 674-676) known to prevent these effects while preserving replication^42,43^. To assess the impact of antiviral RNAi on saRNA, we replaced the GFP stop codon with a P2A self-cleaving site and the NoV B2 gene (CHIKV-GFP-B2^WT^), a known VSR in mammalian cells^23,40,41^. As a control, we used a version with a stop codon instead of the B2 gene (CHIKV-GFP-STOP) (Fig. 1A).

Mouse embryonic stem cells (mESCs) exhibit a potent RNAi response to synthetic dsRNA and viral infections^23,24,44–46^. To assess the impact of NoV B2 expression, we transfected mESCs with CHIKV-GFP-B2^WT^ or CHIKV-GFP-STOP saRNA and measured GFP levels over time by flow cytometry. The ability of the vector to amplify the reporter was defined by gating cells with mean fluorescent intensity (MFI) of 10^3^ or higher (GFP^high^, Fig. 1A and Fig. S1A). The percentage of GFP-positive (GFP^+^) cells reached a maximum of ∼15% GFP^+^ cells 16 h post-transfection and was similar between both saRNAs (Fig. S1B). Compared to CHIKV-GFP-STOP, the B2^WT^-expressing vector exhibited significantly higher MFI values at 16 and 24 h post-transfection, while by 48 h, the difference was no longer detectable (Fig. 1B). Earlier time points showed that GFP expression levels were similar between the two vectors at 3 and 7 h and CHIKV-GFP-B2^WT^ exhibited higher GFP expression only after 10 h (Fig. 1C). To determine whether B2’s dsRNA-binding activity is necessary for enhanced GFP expression, we introduced an R59Q mutation in NoV B2 (CHIKV-GFP-B2^R59Q^), which impairs dsRNA binding and VSR function^32,38,41,47^. Compared to B2^WT^, transfection with CHIKV-GFP-B2^R59Q^ led to a lower percentage of GFP^+^ cells (Fig. 1E) and reduced MFI within GFP^high^ cells (Fig. 1D-1F). These results show that B2, via its dsRNA-binding activity, enhances saRNA-mediated gene expression in mESCs.

To assess whether the *cis*-encoded NoV B2 exhibits VSR activity and inhibits Dicer-mediated processing of saRNAs, we performed small RNA deep sequencing in GFP^+^ mESCs transfected with CHIKV-GFP-B2^WT^ or CHIKV-GFP-B2^R59Q^. In both cases, 15–35 nucleotide (nt) reads comprised ∼16% of total reads. With B2^WT^, ∼28% mapped to the vector with ∼6% in the 20–25 nt range (Fig. S1C). In contrast, B2^R59^^Q^ increased 20–25 nt reads to ∼13–17%. Cells with B2^R59Q^-expressing saRNA also showed an accumulation of 22-nt reads from both the viral positive (+) and negative strands (-) (reaching 7644  reads per million (RPM) and 319 RPM, respectively), characteristic of Dicer activity, whereas B2^WT^ reduced these to 3555 and 89 RPM, consistent with its VSR function (Fig. 1G, H). We next mapped 21-23-nt reads along the saRNA and found that most were derived from the (+)-strand, consistent with the strong (+)-strand bias during alphavirus replication (Fig. 1I, 1J). However, cells with NoV B2^R59Q^-expressing saRNA exhibited increased (-)-strand 21-23-nt reads with ∼36% originating from the first 500 nucleotides of the vector genome corresponding to regions of dsRNA synthesis known to generate Dicer substrates during alphavirus infections (Fig. 1J)^48^. These findings suggest that NoV B2 enhances saRNA-mediated gene expression and concomitantly reduces vsiRNA accumulation and that Dicer targeting of saRNAs may limit their efficiency in mESCs.

### Enhancement of saRNA-mediated protein expression by NoV B2 in somatic cells

**Fig. 2:**
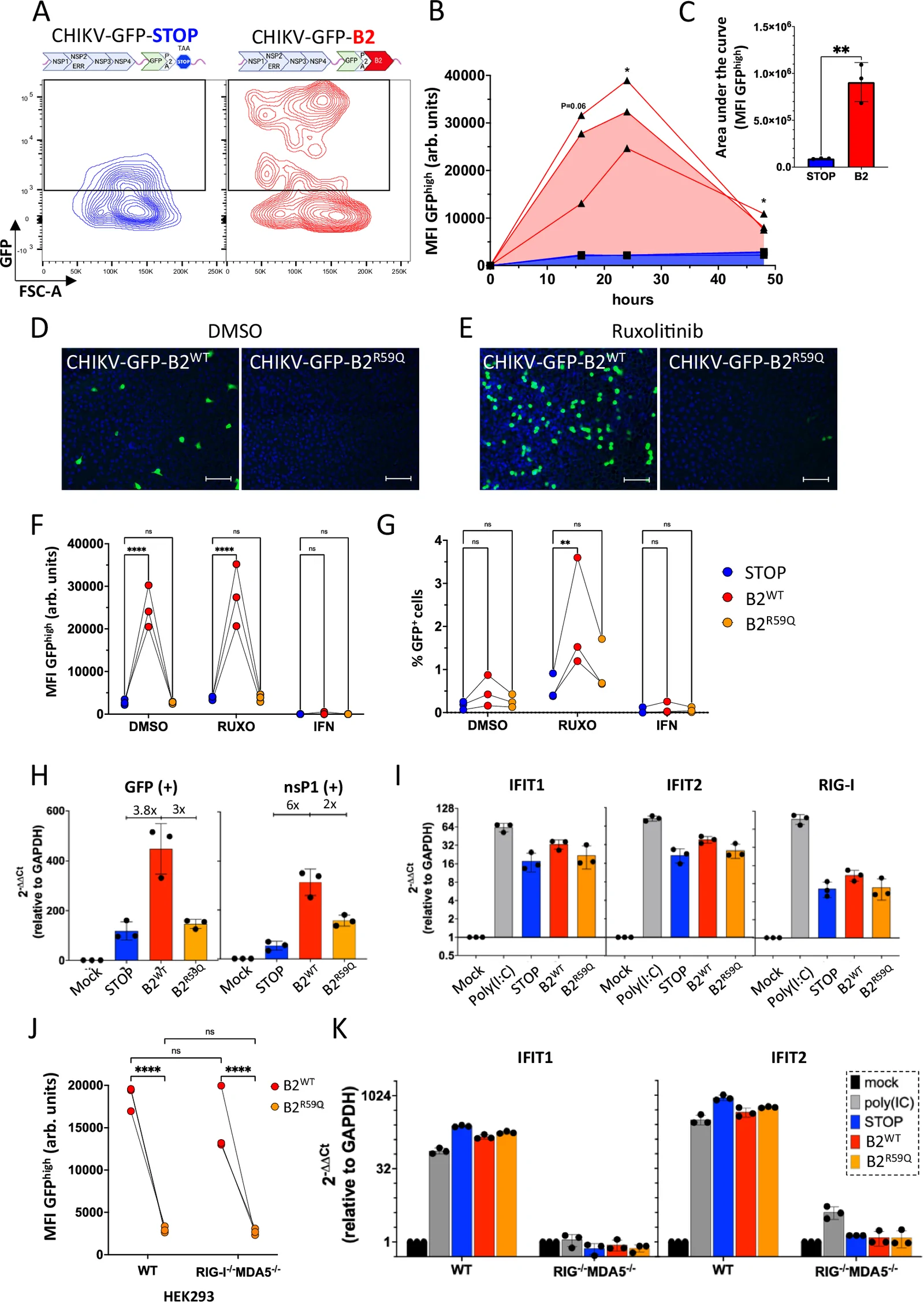
NoV B2 boosts saRNA-mediated gene expression in somatic cells independently of type I IFN response. **A** Representative contour plots of GFP^+^ HeLa cells 24 h post-transfection with CHIKV-GFP-STOP (blue) or -B2^WT^ (red) saRNA. Gate indicates the GFP^high^ population used for MFI analysis in (**B**, **C**). **B** GFP^high^ MFI monitored by flow cytometry in HeLa cells transfected with CHIKV-GFP-STOP (blue) or -B2^WT^ (red) at 16, 24 and 48 h post-transfection. **C** Area under the curve for GFP^high^ MFI values in (**B**). Mean ± s.d., *n* = 3 independent experiments with each point the mean of technical replicates. **D**, **E** Representative images of GFP signal in HeLa cells 24 h post-transfection of CHIKV-GFP-B2^WT^ or -B2^R59Q^ in the presence of DMSO (**D**) or ruxolitinib (**E**). GFP (green), DAPI (blue), scale bar: 100 μm. **F**, **G** GFP^high^ MFI (**F**) or percentage of GFP^+^ cells (**G**) in HeLa cells transfected with CHIKV-GFP-STOP (blue), -B2^WT^ (red) or -B2^R59Q^ (yellow) saRNA in the presence of DMSO, ruxolitinib (RUXO) or recombinant universal IFN-I (IFNα-A/D) 24 h post-transfection. **H, I** HeLa cells transfected with CHIKV-GFP-STOP (blue), -B2^WT^ (red), -B2^R59Q^ (yellow) saRNA or poly(I:C) (100 ng for 24 h). Mean ± s.d., *n* = 3 independent experiments with each point representing the mean of technical replicates. **H** RT-qPCR analysis of mRNA specific to GFP (left) or nsP1 (right) mRNA 24 h post-transfection. **I** RT-qPCR of ISGs IFIT1 (left), IFIT2 (middle) or RIG-I (right) mRNAs. **J** GFP^high^ MFI of WT HEK293 cells or RIG-I^-/-^MDA5^-/-^ cells 24 h post-transfection with of CHIKV-GFP-B2^WT^ (red) or -B2^R59Q^ (yellow). **K** WT and RIG-I^-/-^MDA5^-/-^ HEK293 cells were treated as in (**J**) and IFIT1 (left) and IFIT2 (right) mRNAs were analysed by RT-qPCR. Data are mean ± s.d. from a representative of two biological replicate experiments. Connected dots correspond to values from the same biological replicate. Statistical analyses were performed using a two-tailed ratio paired t-test (**C**) and a two-way ANOVA with Šídák’s multiple comparisons test (**B**), Dunnett’s multiple comparisons test (**F**, **G**) or Fisher’s LSD test (**J**). *P < 0.05; **P < 0.01; ***P < 0.001; ****P < 0.0001; ns not significant. arb. units: arbitrary units. Exact *p* values for (**B**, **C**, **F**, **G**, **J**) and source data are provided the Source Data file.

Antiviral RNAi activity has also been observed in differentiated mammalian cells infected with wild-type^48,49^ or VSR-deficient viruses^40,41^. To test NoV B2’s impact on saRNA efficiency in cell lines, we compared CHIKV-GFP-STOP and CHIKV-GFP-B2^WT^ in HeLa cells, a widely used somatic cell line. In CHIKV-GFP-B2^WT^-transfected cells, GFP expression was robustly enhanced (MFI of 38,000 AU vs 3000 for the control) and this enhancement persisted over time (Fig. 2A–C). We also tested whether NoV B2 could enhance saRNA-mediated gene expression in *trans*. Co-transfection of HeLa cells with CHIKV-GFP-STOP saRNA and a plasmid expressing Strep-tagged NoV-B2 (1xSTreP-NoV B2) increased GFP levels compared to cells transfected with an empty plasmid (Fig. S2A), indicating that NoV B2 boosts saRNA-mediated gene expression both in *cis* and *trans*. Importantly, this enhancement required NoV B2’s dsRNA-binding ability as GFP expression was markedly reduced with saRNAs encoding NoV B2^R59Q^ (Fig. 2D, F). Cells transfected with CHIKV-GFP-B2^WT^ exhibited higher levels of (+)-sense RNA containing *GFP* or *nsP1* (Fig. 2H), while no increase of (-)-sense RNA was observed (Fig. S2B). Similar results were obtained when GFP^high^ cells were isolated by FACS prior to RNA extraction confirming that the increase in RNA levels is not driven by potential differences in the proportion of transfected cells (Fig. S2C). Thus, NoV B2 enhances (+)-strand RNA synthesis and mRNA production through its dsRNA-binding ability.

Unlike mESCs, somatic cells possess a potent type I IFN system. To determine if NoV B2 enhances saRNA-mediated gene expression by inhibiting IFN signalling, we used ruxolitinib, a JAK1/2 inhibitor blocking IFNAR. GFP levels from CHIKV-GFP-B2^R59Q^ were unchanged by ruxolitinib and NoV B2 still enhanced GFP expression with or without treatment (Fig. 2E, F). However, the overall percentage of GFP+ cells was modulated by the IFN signalling: ruxolitinib treatment increased, while pre-treatment with recombinant IFN-I decreased the percentage of GFP^+^ cells following saRNA transfection regardless of NoV B2 (Fig. 2G). These results suggest that while NoV B2 enhances saRNA-mediated gene expression independently of IFN-I signalling, IFN-I influences the overall permissivity of the cell population. Accordingly, no differences in ISGs or cytokine expression (IFIT1, IFIT2, RIG-I and CCL5) were observed following transfection with saRNAs encoding either NoV B2^WT^, B2^R59Q^ or without B2, indicating that the IFN signalling pathway is not impacted (Fig. 2I and Fig. S2D). Furthermore, in RIG-I^-/-^ MDA-5^-/-^ cells, which lack RLR-mediated IFN induction (Fig. S2E), NoV B2 still enhanced GFP expression (Fig. 2J) without affecting RLR-dependent ISG upregulation (Fig. 2K) and resulted in a higher percentage of GFP^+^ cells (Fig. S2F), mirroring the effect of ruxolitinib in HeLa cells (Fig. 2G). Furthermore, to test whether NoV B2 enhancement of saRNA-mediated gene expression persists under conditions of high transfection efficiency and limited innate immunity, we used BHK-21 cells, which are highly permissive to both saRNA transfection and delivery via virus replicon particles (VRPs). VRPs allow saRNAs to be introduced in a particle-based infection context, rather than by direct RNA transfection, providing an alternative delivery route similar to that used by an authentic virus. Both transfection (Fig. S2G-S2I) and VRP-mediated delivery (Fig. S2J, S2K) of CHIKV-GFP-B2^WT^ increased GFP expression compared to CHIKV-GFP-STOP and CHIKV-GFP-B2^R59Q^, suggesting that NoV B2 enhances saRNA-mediated gene expression independent of delivery method and IFN signalling.

**Fig. 3:**
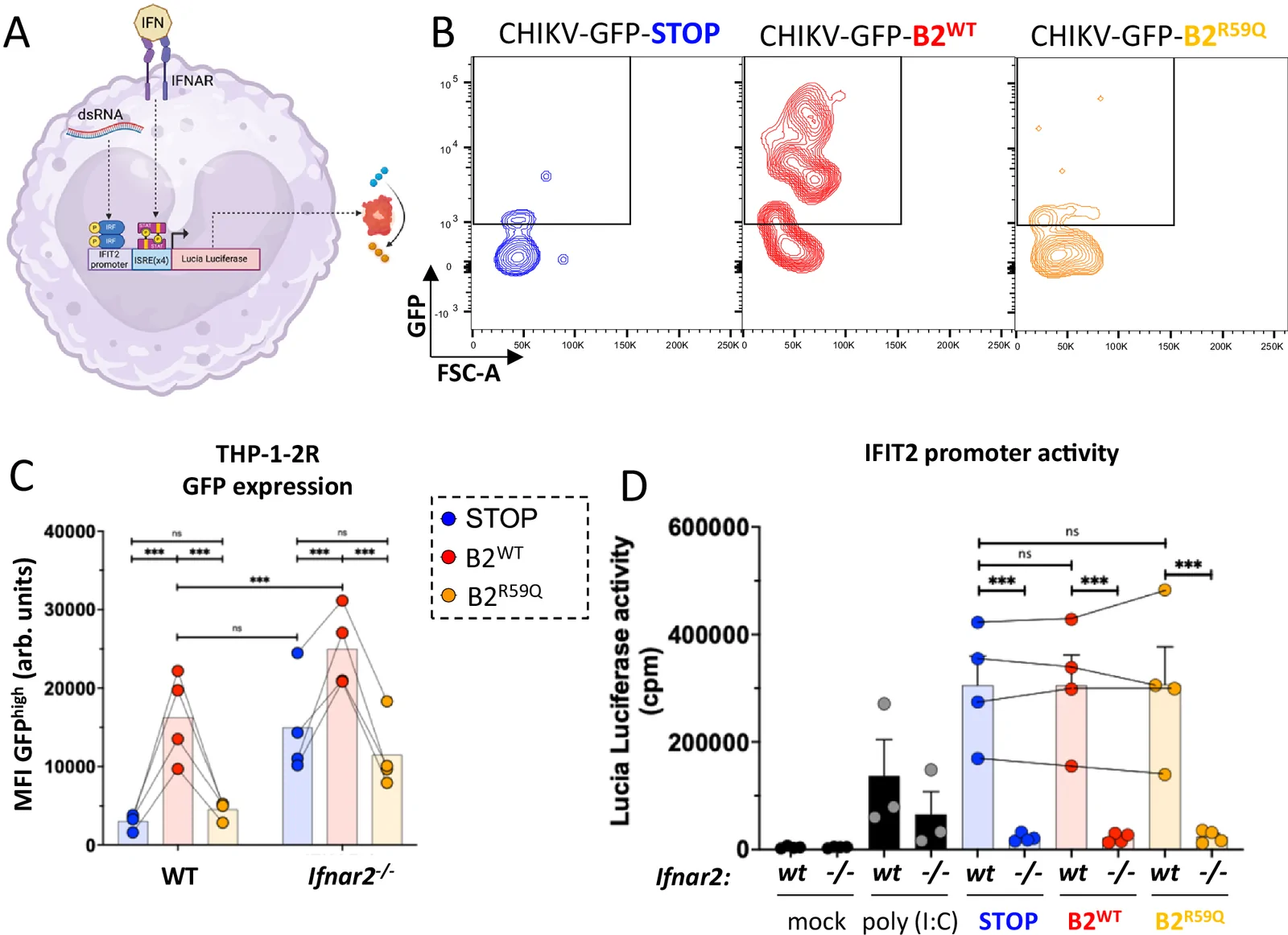
NoV B2 enhances saRNA-mediated transgene expression in monocytic cells while preserving IFN-I signalling. **A** Schematic representation of the THP-1-2R reporter cell line. Upon activation of the IRF and/or the IFN-I signalling pathway, Lucia Luciferase is secreted. Created in BioRender. Maillard, P. (https://BioRender.com/r2k7d4d) is licenced under CC BY 4.0. **B** Representative contour plots of GFP^+^ THP-1-2R reporter cells 24 h post-transfection with CHIKV-GFP-STOP (blue), -B2^WT^ (red) or B2^R59Q^ (orange) saRNA. The indicated gate represents GFP^high^ population selected for MFI analysis in (**C**). **C** THP-1-2R-wt or IFNAR^-/-^ cells were transfected with CHIKV-GFP-STOP (blue), -B2^WT^ (red) or B2^R59Q^ (orange). Flow cytometry was used to monitor the MFI of GFP^high^ population 24 h post-transfection. Mean of independent experiments (*n* = 4), each point represents the average of technical replicates. **D** Immunostimulatory activity of different saRNA vectors in THP-1-2R-wt or IFNAR^-/-^ cells by measuring Lucia Luciferase in the supernatant. For each experiment in (**C**), Lucia luciferase activity was measured. Mean of independent experiments (*n* = 4), each point represents the average of technical replicates. Connected dots correspond to values obtained from the same biological replicate. Statistical analyses were performed using a two-way ANOVA with Tukey’s multiple comparisons test (**B**, **C**). ***P < 0.001; ns not significant. arb. units: arbitrary units. Exact *p* values for (**B**, **C**) and source data are in the Source Data file.

To further characterise NoV B2’s effect on saRNA in an immune-relevant cellular context, we used THP-1-Dual cells (THP-1-2R), a human monocytic cell line that represents a relevant model for innate immune sensing and which reports IFN-I and NF-κB activation via a secreted luciferase and a secreted embryonic alkaline phosphatase (SEAP), respectively (Fig. 3A). CHIKV-GFP-B2^WT^ increased GFP expression compared to CHIKV-GFP-STOP or CHIKV-GFP-B2^R59Q^ (Figs. 3B, C), but didn’t alter IFN-I and NF-κB reporter activity (Fig. 3D and Fig. S3A). In IFNAR-deficient (*Ifnar2*^-/-^) THP-1-2R cells, all three saRNAs exhibited a higher percentage of GFP^+^ cells and CHIKV-GFP-B2^WT^ further increased GFP levels compared to controls (Fig. 3C).

These results show that while type I IFN limits saRNA efficiency, NoV B2 enhances saRNA-mediated gene expression via an IFN-independent mechanism without affecting ISG expression.

### NoV B2 enhances saRNA-mediated gene expression by inhibiting PKR activation in somatic cells

**Fig. 4:**
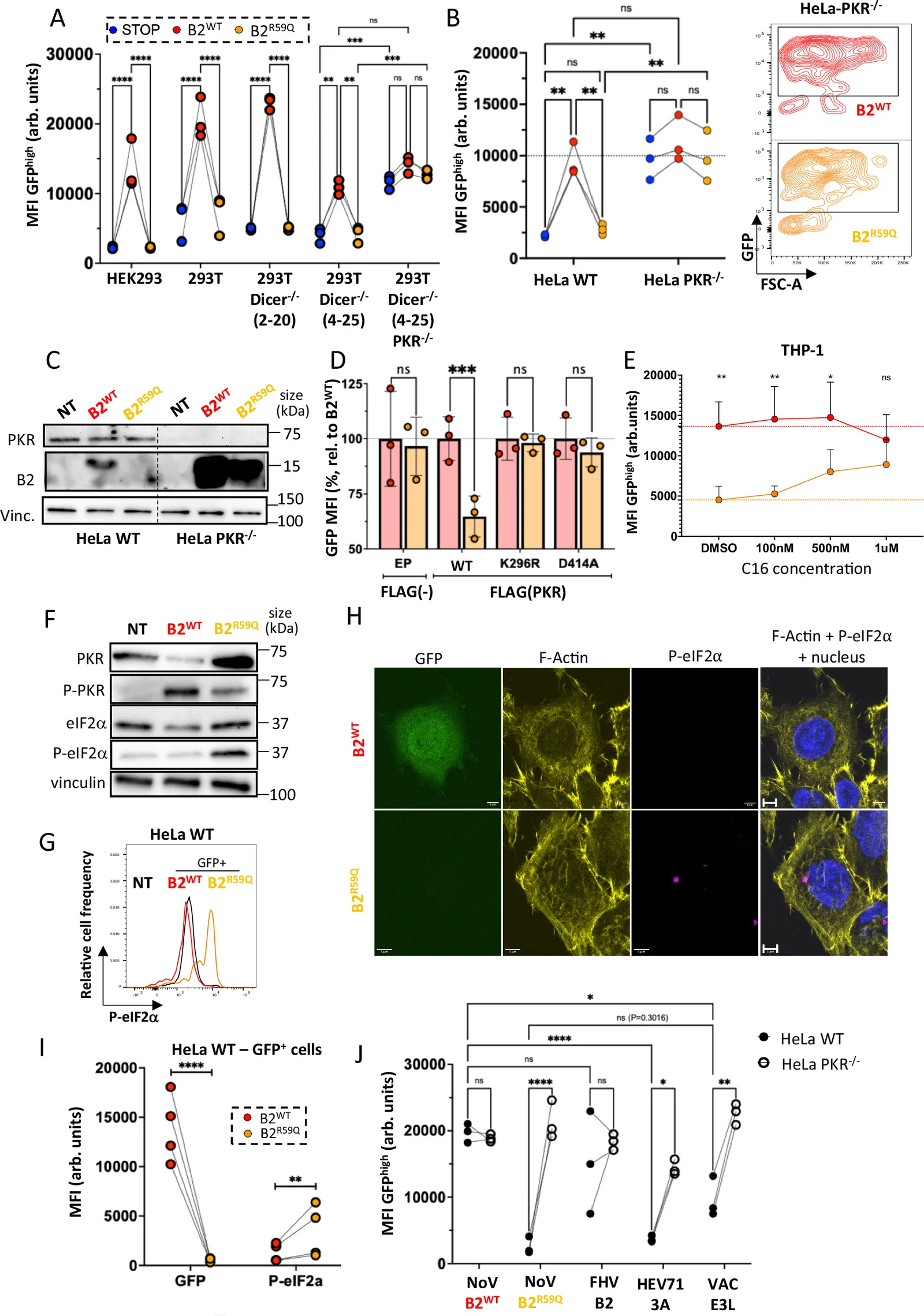
NoV B2 alleviates PKR-mediated restriction of saRNA expression by preventing eIF2α phosphorylation. **A** HEK293, HEK293T, HEK293T *Dicer*^-/-^ (clones 2-20 and 4-25) and HEK293T *Dicer*^-/-^ *PKR*^-/-^ cells (derived from *Dicer*^-/-^ clone 4-25^50,51^) were transfected with CHIKV-GFP-STOP (Blue), -B2^WT^ (red) or -B2^R59Q^ (orange). GFP^high^ MFI measured by flow cytometry 24 h post-transfection. Mean ± s.d, *n* = 3 independent experiments. **B** HeLa WT and *PKR*^-/-^ cells were transfected with CHIKV-GFP-STOP (Blue), -B2^WT^ (red) or -B2^R59Q^ (orange) saRNA (1 µg; 0.1 µg used elsewhere). Mean ± s.d, *n* = 3 independent experiments. Representative contour plots of GFP^ +^ *PKR*^-/-^ cells are shown to the right of the histogram. **C** Western blot showing PKR and B2 expression in HeLa WT and *PKR*^-/-^ cells 24 h post-transfection with CHIKV-GFP-B2^WT^ (red) or -B2^R59Q^ (orange) saRNA. NT, non-transfected control;Vinculin, loading control. **D** HEK293T-*Dicer*^-/-^*PKR*^-/-^ cells were transfected with empty plasmid (EP) or Flag-PKR alongside CHIKV-GFP-B2^WT^ (red) or -B2^R59Q^ (orange) saRNA. GFP MFI in Flag^+^ cells was plotted as percentage relative to B2^WT^, 24 h post-transfection. Mean ± s.d, *n* = 3 independent experiments. **E** THP-1 cells were transfected with CHIKV-GFP-B2^WT^ (red) or -B2^R59Q^ (orange) with increasing C16 concentrations, and analysed by flow cytometry at 24 h post transfection. Mean ± s.d, *n* = 4 independent experiments. **F** Western blot of THP-1 cells 24 h post-transfection with CHIKV-GFP-saRNA expressing indicated B2 proteins. Vinculin, loading control. **G** Histogram of P-eIF2α intracellular staining in GFP^+^ Hela cells 24 h post-transfection with CHIKV-GFP-B2^WT^ or -B2^R59Q^. **H** Representative images of HeLa cells transfected as in (**G**), analysed by confocal microscopy. P-eIF2α (magenta), F-actin (yellow), nuclei (blue), GFP (green); scale bars 5 μm. **I** GFP and P-eIF2α MFI measured by flow cytometry in GFP^+^ Hela cells transfected with CHIKV-GFP-B2^WT^ (red) or -B2^R59Q^ (orange). *n* = 4 independent experiments, each point the mean of technical replicates. **J** GFP^high^ MFI in HeLa WT and *PKR*^-/-^ cells 24 h post-transfection with CHIKV-GFP-saRNA encoding NoV-B2^WT^, B2^R59Q^, FHV-B2, HEV71-3A or vaccinia virus E3L. *n* = 3 independent experiments, each point the mean of technical replicates. Statistical analyses were performed using two-way ANOVA with Šídák’s multiple comparisons test (**A**, **B**, **D**, **E**, **J**) or two-tailed ratio paired *t* test (**I**). *P < 0.05, **P < 0.01, ***P < 0.001; ***P < 0.0001; ns, not significant. arb. units, arbitrary units. Exact *p* values (**A**, **B**, **D**, **E**, **J**) and source data are provided in the Source Data file.

To uncover how NoV B2 enhances saRNA-mediated gene expression, we examined whether it protects saRNAs from Dicer-mediated cleavage since NoV B2 has known VSR activity not only in stem cells but also in somatic cells^41,47^. To explore this, we used human embryonic kidney (HEK) 293 T cells, which are well-suited for this genetic dissection due to the availability of previously described knockout cell lines. We transfected saRNAs expressing either NoV B2^WT^, B2^R59Q^ or no B2 into HEK293T cells lacking Dicer (*Dicer*^-/-^)^50^ or both Dicer and PKR (*Dicer*^-/-^ *PKR*^-/-^) (Fig. 4A and Fig. S4A)^50,51^. The latter was included in our analysis based on previous evidence that Dicer’s antiviral activity against alphaviruses such as Sindbis virus (SINV) and Semliki Forest virus (SFV) depends on the presence of PKR^52,53^. The *Dicer*^-/-^ *PKR*^-/-^ line was originally generated by knocking out *PKR* in a previously described *Dicer*^-/-^ clone (clone 4-25)^50,51^. In addition to this parental clone, we also included a second independent *Dicer*^-/-^ clone (clone 2-20) in our analysis. As in HeLa and THP-1 cells, NoV B2^WT^ expression resulted in a higher MFI in the GFP^high^ population in HEK293 and HEK293T cells used as controls (Fig. 4A). However, GFP expression from CHIKV-GFP-STOP and CHIKV-GFP-B2^R59Q^ remained low in *Dicer*^-/-^ cells and NoV B2^WT^ -mediated enhancement remained unaffected (Fig. 4A). Strikingly, this restriction was lost in *Dicer*^-/-^ *PKR*^-/-^ cells where GFP levels were similar across all saRNAs independently of their expression of a functional NoV B2 (Fig. 4A). Of note, GFP expression in *Dicer*^-/-^ *PKR*^-/-^ cells transfected with CHIKV-GFP-B2^WT^ was lower than in WT or *Dicer*^-/-^ (clone 2-20) cells, yet was comparable to that observed in the parental *Dicer*^-/-^ clone 4-25. Consistent with these findings, analysis in HeLa WT and HeLa *PKR*^-/-^ cells showed enhanced GFP expression from CHIKV-GFP-B2^WT^ in WT cells compared to CHIKV-GFP-STOP and CHIKV-GFP-B2^R59Q^ (Fig. 4B). In contrast, in HeLa *PKR*^-/-^ cells, the loss of PKR alone was sufficient to relieve restriction and, consequently, enhancement conferred by NoV B2 was markedly reduced with GFP levels similar to those observed with CHIKV-GFP-STOP and CHIKV-GFP-B2^R59Q^ (Fig. 4B). Furthermore, while saRNA-mediated expression of NoV B2^R59Q^ was undetectable in HeLa cells, both wild-type and mutant versions of NoV B2 were readily detected in HeLa *PKR*^-/-^ cells, demonstrating the loss of restriction (Fig. 4C). Thus, PKR restricts saRNAs and the *cis*-encoded NoV B2 alleviates this inhibition.

To further characterise PKR restriction of saRNA, we complemented *Dicer*^-/-^ *PKR*^-/-^ HEK293T cells with FLAG-tagged constructs encoding either wild-type or mutant versions of PKR. We included PKR^K296R^ (defective in ATP binding, dimerisation and phosphorylation)^54^ and PKR^D414A^ (kinase catalytic mutant)^55^. One day after PKR construct transfection, cells were transfected with CHIKV-GFP-B2^WT^ or CHIKV-GFP-B2^R59Q^ and GFP levels were assessed in FLAG^+^ cells by flow cytometry (Fig. S4B). PKR^WT^ reduced GFP expression from the NoV B2^R59Q^-expressing saRNA relative to the B2^WT^-expressing vector, while both PKR mutants failed to restore this restriction indicating that PKR’s kinase activity is essential (Fig. 4D). PKR^WT^ expression was lower than that of PKR^K296R^ and PKR^D414A^ (Fig. S4C). Yet, phosphorylated PKR (P-PKR) was detected uniquely in PKR^WT^-expressing cells, whereas both PKR mutants were either completely (PKR^K296R^) or partially (PKR^D414A^) lacking phosphorylation (Fig. S4C).

To confirm that PKR restricts saRNAs and that NoV B2 counteracts this effect, we transfected THP-1 cells with CHIKV-GFP-B2^WT^ or CHIKV-GFP -B2^R59Q^ in the presence of increasing concentrations of the PKR inhibitor C16, which blocks PKR autophosphorylation^56,57^. In cells transfected with CHIKV-GFP-B2^R59Q^, GFP expression increased in a dose-dependent manner with C16, while no enhancement was observed with CHIKV-GFP-B2^WT^ and GFP levels equalised between the two vectors at 1 µM of C16 (Fig. 4E, Fig. S4D). Similar results were observed in *Ifnar2*^-/-^ THP-1 cells, where 500 nM C16 abolished the GFP expression difference between the two saRNAs (Fig. S4E); the fact that the effect was observed at a lower dose of inhibitor likely reflects lower PKR expression in the absence of IFN-I signalling. Notably, C16 treatment also increased the percentage of GFP^+^ cells up to eightfold for both saRNAs suggesting that blocking steady-state level of PKR activity improves cellular permissivity to saRNAs (Fig. S4F). These results demonstrate that catalytically active PKR restricts saRNA activity and that NoV B2 alleviates this restriction.

We next examined at which step NoV B2 inhibits the PKR pathway. Upon binding to dsRNA, PKR is activated through auto-phosphorylation (P-PKR), which subsequently leads to phosphorylation of eIF2α (P-eIF2α) resulting in translational shutdown. We therefore assessed P-PKR and P-eIF2α levels in cells transfected with saRNAs encoding either functional or dsRNA-binding defective NoV B2. While P-PKR levels were similar in THP-1 cells transfected with either CHIKV-GFP-B2^WT^ or CHIKV-GFP-B2^R59Q^ (Fig. 4F), no P-eIF2α was detected in CHIKV-GFP-B2-transfected cells where NoV B2 protein was readily expressed (Fig. 4F). Flow cytometry and confocal microscopy confirmed that P-eIF2α levels were detectable in CHIKV-GFP-B2^R59Q^- transfected cells but absent in cells transfected with CHIKV-GFP-B2^WT^ (Fig. 4G, H). These levels of P-eIF2α inversely correlated with GFP expression from the saRNAs (Fig. 4I). Thus, NoV B2 expressed from saRNA doesn’t prevent PKR autophosphorylation but blocks eIF2α phosphorylation, bypassing translation inhibition.

We next compared NoV B2 with other VSRs. While Flock house virus B2 (FHV B2) and human enterovirus 71 3A (HEV71 3A) have been shown to suppress Dicer activity^32,58,59^, vaccinia virus E3L (VACV E3L) has been shown to inhibit both Dicer and PKR activation^60,61^. Furthermore, VACV E3L protein expression in *trans* has been shown to enhance vector gene delivery by inhibiting PKR activation and the IFN response^62^. We replaced the NoV B2 in CHIKV-GFP-B2 with FHV B2, HEV71 3A or VACV E3L and tested saRNA efficiency in both wild-type HeLa and *PKR*^-/-^ cells (Fig. 4J). FHV B2 enhanced saRNA activity similarly to NoV B2, while HEV71 3A did not counteract this restriction. However, VACV E3L only modestly improved GFP levels compared to NoV B2^R59Q^ and VACV E3L-expressing saRNAs exhibited lower activity in wild-type relative to *PKR*^-/-^ cells, suggesting only partial inhibition of PKR. Thus, among the tested VSRs, NoV B2 and FHV B2 most effectively alleviate PKR-mediated restriction of saRNAs.

### *Cis*-expression of NoV B2 results in a change of dsRNA spatial distribution within the cells

**Fig. 5:**
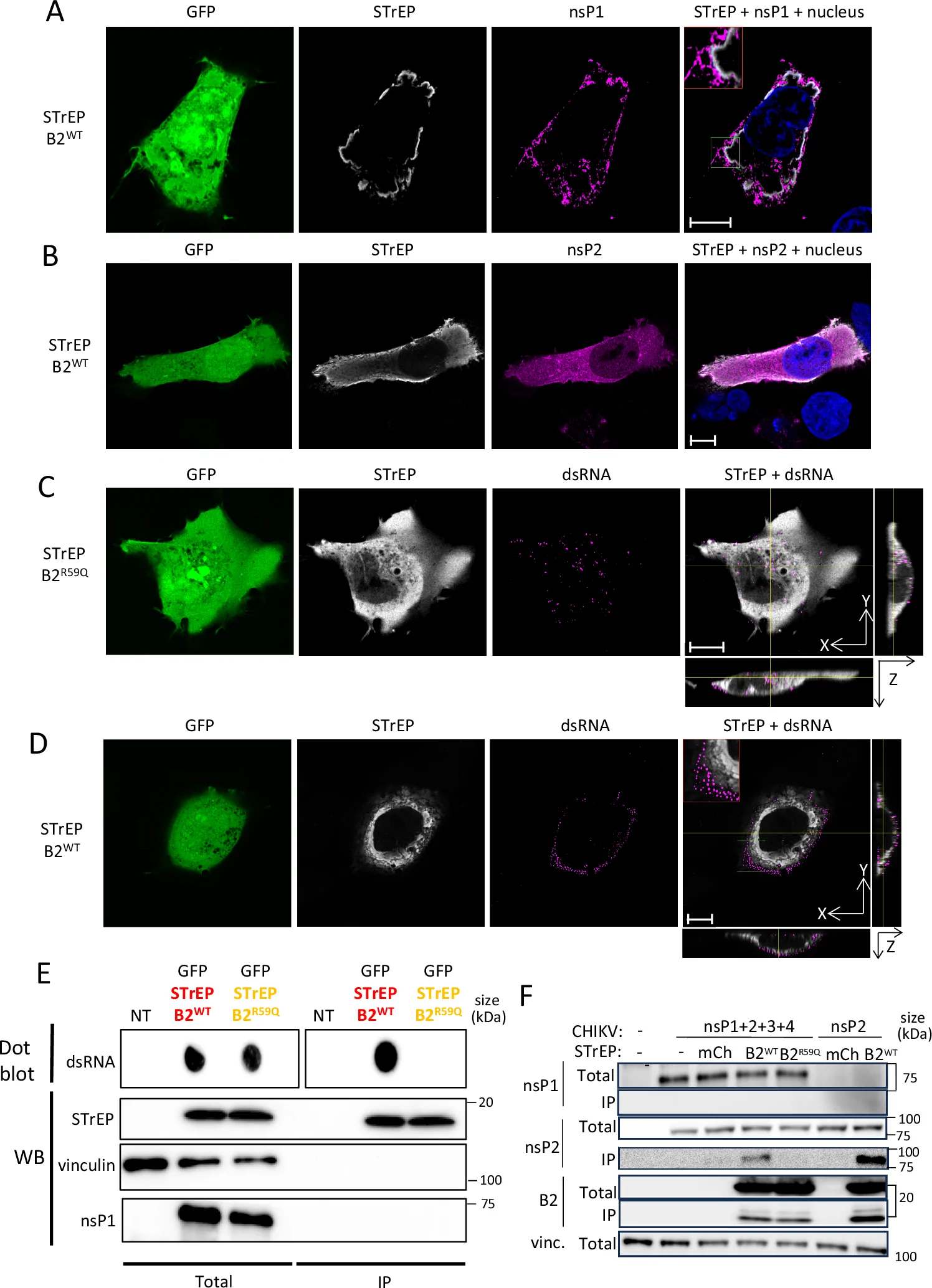
NoV B2 redistributes viral dsRNA to the cell periphery and interacts with nsP2 in a dsRNA-binding-dependent manner. **A**, **B** Representative images of HeLa cells transfected with CHIKV-GFP-STrEP-B2^WT^ saRNA, stained at 20 h post-transfection for nucleus (blue), STrEP-tag (white) and nsP1 (**A**) or nsP2 (**B**) (magenta). Red square in the merged panel shows a magnification of the green square in the same image. Scale bar is 10 µm. Data are representative of two independent experiments. **C**, **D** Representative images of HeLa *PKR*^-/-^ cells transfected with CHIKV-GFP-STrEP-B2^R59Q^ (**C**) or CHIKV-GFP-STrEP-B2^WT^ (**D**) saRNA. Cells were stained at 20 h post-transfection for STrEP-tag (white) and dsRNA (magenta). Red square in merged panel shows a magnification of the green square in the same image. Panels lining the merged images represent the orthogonal views of the distribution of STrEP and dsRNA staining. Scale bars is 10 μm. Data are representative of two independent experiments. **E** HeLa *PKR*^-/-^ cells were transfected with CHIKV GFP-STrEP-B2^WT^ or -B2^R59Q^ saRNA. At 24 h post-transfection STrEP-mediated immunoprecipitation was performed, followed by dot blot analysis and western blotting. dsRNA levels were assessed by dot blot analysis in total lysate (input) and immunoprecipitated fractions (IP) (top panels). Expression levels of STrEP-tagged B2 proteins and nsP2 were assessed in total and IP fractions by western blotting (WB, bottom panels). Vinculin served as a loading control. Data are representative of three independent experiments. **F** HEK293T cells were co-transfected with the indicated STrEP-tagged constructs (STrEP-mCherry, -NoV-B2^WT^ or -NoV-B2^R59Q^) and a combination of plasmids encoding nsP1, nsP2, nsP3 and nsP4 or a plasmid encoding nsP2 alone. After 24 h, STrEP-mediated immunoprecipitation was performed. nsP1, nsP2 and B2 levels were assessed by western blot. Vinculin served as a loading control. Source data are provided as a Source Data file.

To further investigate the mechanism by which B2 enhances saRNA-mediated gene expression, we analysed its subcellular localisation by confocal microscopy. A functional N-terminal STrEP-tagged NoV B2 protein was expressed *in cis* from a CHIKV-GFP saRNA and was detected predominantly at the cell periphery (Fig. 5A). Since the alphavirus RNA replication complex (vRC) forms at the plasma membrane, we compared B2 localisation to that of nsP1, a membrane-anchored vRC component that facilitates spherule formation, invaginations that harbour viral dsRNA intermediates^63–67^. While nsP1 localised as expected at the cell periphery, B2 was found adjacent to, but not overlapping with, nsP1 (Fig. 5A).

We next analysed nsP2, a vRC component located on the cytoplasmic side of the spherules^65^. nsP2 exhibited a diffused cytoplasmic distribution with partial colocalisation with B2 at the cell periphery (Fig. 5B). We then examined the localisation of STrEP-tagged B2^R59Q^. As CHIKV saRNAs encoding a non-functional NoV B2 are restricted in HeLa cells, we assessed the mutant distribution in HeLa *PKR*^-/-^ cells. While NoV B2^WT^ still localised to the periphery, NoV B2^R59Q^ was diffusely distributed throughout the cytoplasm (Figs. 5C, 5D). A time-course experiment at 8, 12 and 24 h post-transfection, including Phalloidin staining to label F-actin and delineate the cell morphology, revealed that NoV B2^WT^ is progressively excluded from the perinuclear region and accumulates at the cell periphery. In contrast, NoV B2^R59Q^ remained distributed throughout the cytoplasm at all time points examined (Fig. S5A). Similar results were obtained in *Ifnar2*^-/-^ THP-1 cells where NoV B2^WT^ localised predominantly at the cell periphery and showed limited overlap with GFP, whereas NoV B2^R59Q^ exhibited greater co-localisation with cytoplasmic GFP (Fig. S5B, S5C). Given the importance of NoV B2’s dsRNA-binding activity for its localisation, we assessed dsRNA distribution in HeLa *PKR*^-/-^ cells transfected with CHIKV saRNA encoding either STrEP-B2^WT^ or -B2^R59Q^. At 20 h post-transfection, in the absence of functional NoV B2, dsRNA appeared broadly dispersed throughout the cytoplasm (Fig. 5C, Fig. S6A). In contrast, in B2^WT^-expressing cells, dsRNA showed a greater enrichment at the cell periphery with comparatively less signal distributed throughout the cytoplasm (Fig. 5D, Fig. S6A). Orthogonal views showed NoV B2 localised just beneath dsRNA foci, without direct overlap (Fig. 5D). A similar dsRNA distribution was observed at 36 h post-transfection, suggesting that this pattern persists at later time points (Fig. S6B). To further examine the subcellular localisation of the cytoplasmic dsRNA observed in cells transfected with CHIKV-GFP-STrEP-B2^R59Q^, we co-stained cells for nsP1 and dsRNA. In these cells, dsRNA was detected within nsP1-enriched cytoplasmic structures consistent with alphavirus replication compartments such as type I cytopathic vacuoles (CPV-I). However, dsRNA signal was also observed in regions lacking nsP1, indicating that dsRNA is not exclusively confined to these structures and can also be detected in the surrounding cytoplasm (Fig. S6C).

Given NoV B2’s localisation beneath the vRC, we first sought to biochemically confirm that B2 binds dsRNA in saRNA-transfected cells. Immunoprecipitation of STrEP-tagged B2 proteins from CHIKV saRNA-transfected HeLa *PKR*^-/-^ cells followed by dot-blot analysis revealed that dsRNA co-immunoprecipitated with NoV B2^WT^ but not with NoV B2^R59Q^, validating B2’s dsRNA binding activity in this context (Fig. 5E). We next tested whether NoV B2 interacts with CHIKV replication proteins. Both NoV protein A, the viral RNA replicase, and alphavirus nsP1 have been shown to multimerise into 12-mer ring-like structures anchored at cellular membranes and share homologous MTase/GTase domains^64,68^. Furthermore, the B2 protein from FHV, another member of the Nodaviridae family closely related to NoV, was previously shown to co-immunoprecipitate with Protein A in FHV-infected insect cells, supporting an association between nodaviral B2 proteins and the viral replication machinery^32,69^. Based on these parallels, we hypothesised that NoV B2 might similarly associate with components of the alphavirus replication complex when expressed from CHIKV saRNAs. We therefore co-expressed STrEP-tagged B2^WT^ or B2^R59Q^ with either a combination of expression plasmids encoding for CHIKV nsP1, nsP2, nsP3 and nsP4 or a plasmid encoding nsP2 alone and assessed interactions by STrEP immunoprecipitation. NoV B2^WT^ specifically co-precipitated with nsP2, and not nsP1, and this interaction was independent of other vRC components (Fig. 5F). In contrast, NoV B2^R59Q^ was no longer able to interact with nsP2 (Fig. 5F). Deletion of the C-terminal methyltransferase (MTase)-like domain of nsP2 also abolished binding to NoV B2 (Fig. S6D). The MTase-like domain of nsP2 is distally located from the spherule, faces the cytosol and is exposed enough to be able to functionally interact with different proteins, including host factors of CHIKV replication^70^. Together, these results indicate that NoV B2’s dsRNA-binding activity is required for its peripheral localisation, its interaction with nsP2 and its ability to alleviate PKR restriction, consistent with a model in which these features are mechanistically linked.

### NoV B2 enhances gene expression from VEEV-based saRNAs

To assess whether NoV B2 also boosts gene expression from saRNAs derived from other alphavirus genomes, we assessed its impact on Semliki Forest virus (SFV)-based saRNAs encoding GFP in the presence or absence of NoV B2. Transfection of SFV-GFP-STOP, SFV-GFP-B2^WT^ and SFV-GFP-B2^R59Q^ in HEK293, BHK-21, THP-1 and HeLa WT cells consistently showed enhanced GFP expression in the presence of functional NoV B2 compared to SFV-GFP-STOP and SFV-GFP-B2^R59Q^ (Fig. S7A). Furthermore, this restriction was markedly reduced in HeLa *PKR*^-/-^ cells, confirming that the effect is PKR-dependent.

**Fig. 6:**
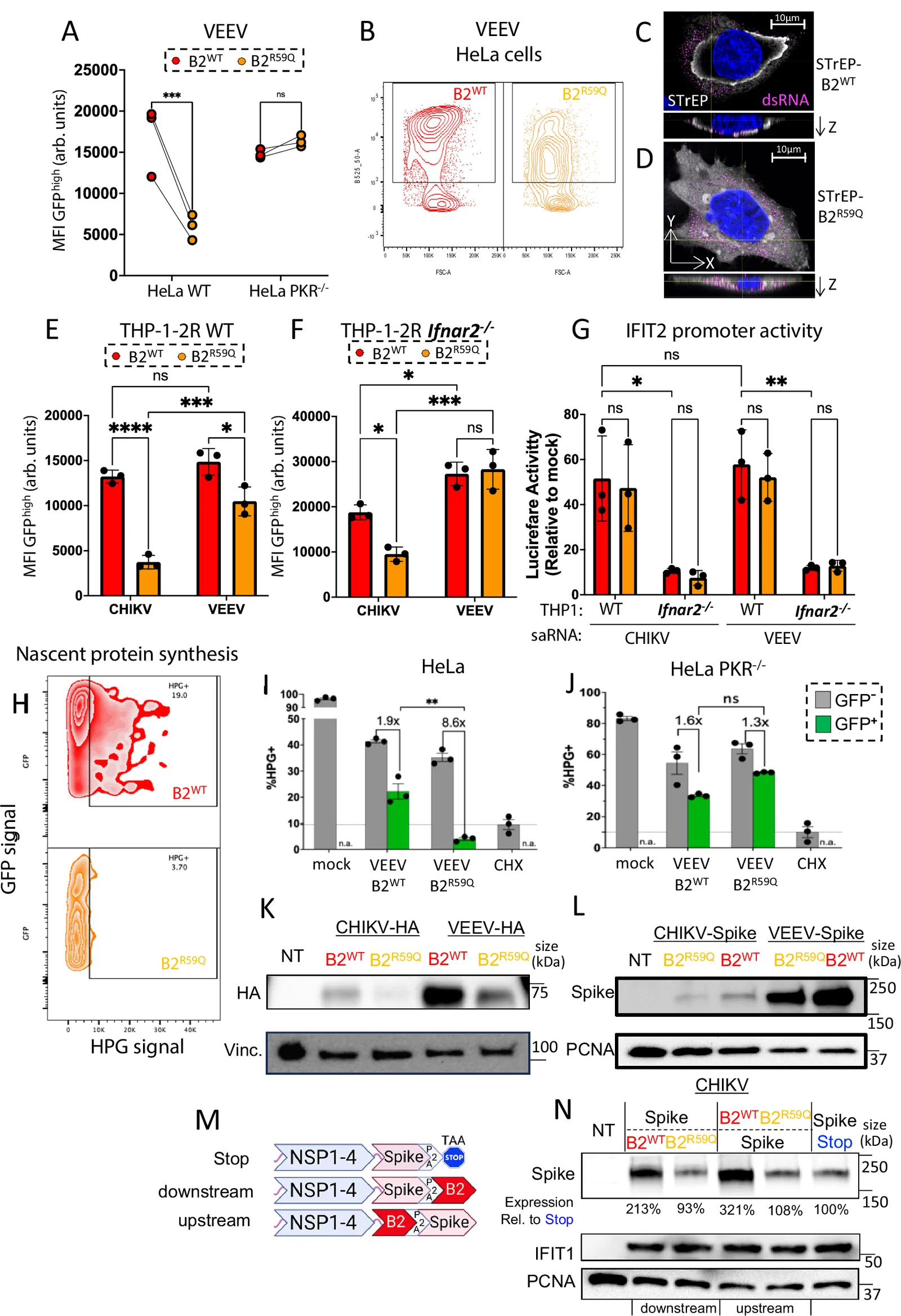
NoV B2 boosts expression from VEEV-based saRNA by counteracting PKR-dependent translational inhibition without altering IFN responses. **A** HeLa WT and *PKR*^-/-^ cells were transfected with VEEV-GFP-B2^WT^ (red) or -B2^R59Q^ (orange). GFP^high^ MFI was measured by flow cytometry 24 h post-transfection. *n* = 3 independent experiments, each point the mean of technical replicates. **B** Representative contour plots of GFP^+^ HeLa cells 24 h post-transfection with VEEV-GFP-B2^WT^ or -B2^R59Q^ saRNA. **C**, **D** Confocal microscopy images of HeLa WT cells transfected with VEEV-GFP-STrEP-B2^WT^ (**C**) or -STrEP-B2^R59Q^ (**D**) stained 20 h post-transfection for STrEP (white), dsRNA (magenta) and nuclei (blue). Lower panel: orthogonal projection of STrEP and dsRNA. Scale bars: 10 μm. Representative of four independent experiments. **E**, **F** GFP^high^ MFI was measured by flow cytometry in THP-1-2R-WT (**E**) or THP-1-2R-IFNAR^-/-^(**F**) cells transfected with CHIKV-GFP-B2^WT^ (red), -B2^R59Q^ (orange), VEEV-GFP-B2^WT^ (red) or -B2^R59Q^ (orange). Mean ± s.d., *n* = 3 independent experiments, each point the mean of technical replicates. **G** Immunostimulatory activity of saRNA vectors in THP-1-2R-WT or -IFNAR^-/-^ cells measured by Lucia Luciferase activity in supernatants from (**E**, **F**). Mean ± s.d., *n* = 3 independent experiments, each point the mean of technical replicates. **H–J** Nascent protein synthesis monitored by flow cytometry in HeLa WT (**H**, I) or *PKR*^-/-^ (**J**) cells transfected with VEEV-GFP-B2^WT^ or -B2^R59Q^ saRNA. **H** Representative contour plot of GFP^+^ HeLa cells after HPG labelling. **I, J** HPG incorporation in GFP^+^ and GFP^-^ cells. Mean ± s.d., *n* = 3 independent experiments, each point the mean of technical replicates. **K** Western blot of IAV HA expression in HEK293T cells 24 h post-transfection with CHIKV-HA- B2^WT^, -B2^R59Q^ or VEEV-HA-B2^WT^ or -B2^R59Q^ saRNA. **L** Western blot of SARS-CoV-2 Spike prefusion protein in HEK293T.17 cells 24 h post-transfection with CHIKV-Spike- B2^WT^, -B2^R59Q^, VEEV-Spike-B2^WT^ or -B2^R59Q^ saRNA. **M** Schematic of CHIKV-Spike saRNA with B2^WT^ or B2^R59Q^ inserted upstream or downstream of Spike. **N** Western blot of SARS-CoV-2 Spike in HeLa WT cells 24 h post-transfection with saRNAs depicted in (**M**) encoding NoV-B2^WT^ or NoV-B2^R59Q^ upstream or downstream of Spike. Statistical analyses were performed using two-way ANOVA with Šídák’s multiple comparisons test (**A**, **E**, **F**, **G**, **I**, **J**). *P < 0.05, **P < 0.01, ***P < 0.001, ****P < 0.0001; ns, not significant. arb. units, arbitrary units. Exact *p* values (**A**, **E**, **F**, **G**, **I**, **J**) and source data are provided in the Source Data file.

We next examined Venezuelan equine encephalitis virus (VEEV)-based saRNAs that are currently used for vaccine studies^2^. VEEV-based vectors encoding GFP and either NoV B2^WT^ or B2^R59Q^ were tested in various cell types. VEEV-based saRNAs were more efficient and displayed a higher percentage of GFP^+^ cells in HEK293, HEK293T and THP-1 cells compared to CHIKV-based saRNAs (Fig. S7B). HeLa cells remained the most restrictive cells for both vectors. In HeLa cells, the *cis*-expression of NoV B2 from VEEV saRNA increased the MFI of the GFP^high^ population, an effect that was lost in *PKR*^-/-^ cells, confirming PKR as the main restriction factor (Fig. 6A, B). Confocal microscopy showed that NoV B2^WT^ localised to the cell periphery and dsRNA foci formed an outer layer (Fig. 6C and Fig. S7C), whereas in NoV B2^R59Q^-expressing cells both B2^R59Q^ and dsRNA remained diffuse in the cytoplasm, similar to observations with CHIKV saRNAs (Fig. 6D and Fig. S7C). Thus, VEEV-derived vectors, like CHIKV-based saRNAs, are restricted by PKR, and NoV B2 limits the redistribution of the dsRNA to the cytoplasm.

In THP-1 cells, VEEV-GFP-B2^R59Q^ saRNA displayed significantly higher activity than CHIKV-GFP-B2^R59Q^, suggesting that VEEV is naturally less sensitive to PKR activity in THP-1 cells (Fig. 6E). Nonetheless, NoV B2 further significantly enhanced gene expression from VEEV-based saRNAs and its expression abolished the differences in GFP expression observed between CHIKV and VEEV-based vectors (Fig. 6E). This suggests that PKR inhibition by NoV B2 overcomes inherent differences in vector sensitivity. Importantly, as previously observed with CHIKV-based saRNA, there was a similar activation of the IFN-I response in cells transfected with VEEV saRNA expressing NoV B2^WT^ or B2^R59Q^, but the B2-mediated enhancement of GFP expression was lost in *Ifnar2*^-/-^ THP-1-2R cells (Fig. 6F, G), suggesting that the boosted expression of PKR upon IFN-I signalling amplification is responsible for the restriction in the VEEV system.

To explore the broader impact of NoV B2 on the global host translation, we measured nascent protein synthesis using the incorporation of L-homopropargylglycine (HPG), a methionine analogue which can stain cells by click chemistry^71^. VEEV saRNA transfection induced a strong reduction in protein synthesis across the entire cell population (Fig. S7D). HPG incorporation was reduced by more than 50% in the bulk population, reaching levels comparable to cells treated with cycloheximide (CHX), a widely used translation inhibitor (Fig. S7D). To differentiate between saRNA-active and inactive cells, we measured HPG incorporation specifically in the GFP⁺ population (Fig. S7E). In GFP^+^ cells, VEEV saRNA harbouring NoV B2^R59Q^ caused near-complete translational shutdown, showing an 8.6-fold reduction compared to GFP⁻ cells, while the presence of NoV B2^WT^ partially restored protein synthesis (Fig. 6H, I). This rescue was PKR-dependent as it was absent in HeLa *PKR*⁻/⁻ cells (Fig. 6J). Thus, the inclusion of B2^WT^ not only enhances transgene expression but also inhibits the global translational shutdown of host mRNAs, promoting a more permissive environment for protein synthesis. Finally, NoV B2^WT^ enhanced expression of clinically relevant antigens such as Influenza A virus (IAV) hemagglutinin (HA) or the prefusion form of the SARS-CoV-2 Spike protein from both CHIKV and VEEV vectors (Figs. 6K, L). In addition, we examined how the position of the B2 gene affects antigen expression in CHIKV-based vectors in HeLa cells. Placing NoV B2^WT^ downstream of the Spike gene resulted in a 2-fold increase in protein expression, while positioning it upstream yielded a 3-fold increase (Figs. 6M, N). This positional effect was confirmed in HEK293T cells where upstream positioning of NoV B2^WT^ similarly resulted in greater Spike expression compared to downstream positioning (Fig. S7F). In contrast, B2^R59Q^ had no effect on antigen expression regardless of position or cell line (Fig. 6N, S7F). Consistent with our previous observations, NoV B2^WT^ did not alter the type I IFN response since the expression of IFIT1, a well-established ISG, remained unchanged across all five tested vectors (Fig. 6N).

## Discussion

saRNA boosts antigen expression and acts as a self-adjuvant, but cytosolic dsRNA generated during replication also activates innate antiviral responses that restrict its full efficacy. Here we show that *cis*-expression of NoV B2 protein enhances saRNA-mediated protein expression from CHIKV, SFV or VEEV-based vectors, three of the most promising saRNA backbones for vaccination^72^, in both stem cells and somatic cells.

In mESCs, CHIKV saRNAs triggered accumulation of 21-23-nt small RNAs primarily mapping to the 5’ end of the vector’s genome and the transcription start site of the subgenomic RNA. These reads derived from both strands, indicative of Dicer processing of dsRNA replication intermediates, consistent with antiviral RNAi previously shown to be active in stem cells^18,23–25^. Similar patterns of vsiRNA accumulation have been reported in infections with viruses restricted by antiviral RNAi, such as SFV, SINV, HEV-71, Zika virus and NoV^25,32,41,48,58,73^. The abundance of vsiRNA detected in cells transfected with CHIKV-based saRNAs might be dampened by CHIKV nsP2 and nsP3, which have been shown to suppress shRNA-mediated RNAi in overexpression assays^74^, although whether they function as bona fide VSRs during infection remains to be established. *Cis*-expression of NoV B2, a well-established VSR^21,30,38,39^, reduced 21-23-nt vsiRNA accumulation and enhanced saRNA-mediated protein expression, suggesting that Dicer-mediated processing limits saRNA efficiency in mESCs and that this can be overcome by a VSR. However, we have not directly tested this using genetic depletion of Dicer or Ago2 and we cannot exclude a contribution from other B2-sensitive pathways. Genetic loss-of-function approaches will be needed to formally establish the functional contribution of antiviral RNAi in this context. These findings may extend to other stem cell types, including human induced pluripotent stem cells (hiPSCs) and adult stem cells that express aviD, a Dicer isoform with enhanced dsRNA processing activity^24^.

While antiviral RNAi activity declines upon cellular differentiation, some activity persists in somatic cells infected with VSR-deficient viruses^41,48,49,58,75^. We found that NoV B2 enhanced saRNA-driven gene expression in three IFN-competent somatic cell lines (i.e., HeLa, THP-1 and HEK293 cells) without suppressing ISG induction. While the exact ligand of saRNA recognised by RLRs needs to be identified, parental CHIKV infections would suggest that the 3’UTR of the genomic and subgenomic RNA would still harbour immunostimulatory activity^76^. Our findings align with previous studies showing that NoV B2 selectively suppresses RNAi without impairing IFN-I responses^40,41,77^. Indeed, B2 expression from a NoV-derived replicon had minimal impact on ISGs or IFN-β and NoV infection of mouse embryonic fibroblasts still triggered a robust RIG-I-mediated IFN response^41,77^. Furthermore, a comprehensive RT-qPCR analysis of 84 key innate immune genes in NoV-infected suckling mice detected no major differences in innate immune gene expression between WT NoV, which encodes NoV B2, and a B2-deficient mutant^40^. This is notably distinct from previous studies showing that *cis*-expression of the V protein from PIV-5 (parainfluenza virus-5) or ORF4a from MERS-CoV (Middle East respiratory syndrome coronavirus) enhanced saRNA-mediated protein expression by suppressing the IFN-I pathway^17^. Thus, NoV B2 uniquely boosts saRNA-gene expression without impairing the induction and signalling of IFN-I, potentially preserving the saRNA’s self-adjuvant properties, a key advantage over strategies that suppress IFN signalling.

Our analysis further shows that in somatic cells, NoV B2 enhances saRNA-mediated protein expression through a mechanism independent of Dicer. Instead, PKR emerges as a primary restriction factor that restricts CHIKV, SFV and VEEV saRNAs and NoV B2 confers resistance to this inhibition. Complementation experiments confirmed that this restriction was dependent on PKR activity and that *cis*-expression of NoV B2 prevented the phosphorylation of eIF2α, a key PKR substrate, thereby preventing translational shutdown. This function requires NoV B2’s dsRNA-binding activity as NoV B2^R59Q^ failed to inhibit eIF2α phosphorylation. While NoV B2’s ability to bind and shield dsRNA from Dicer’s activity is well-documented, our findings reveal an additional role in inhibiting the PKR pathway when expressed from alphavirus saRNAs. Whether this PKR-suppressive function of B2 also operates during NoV infections in mammalian cells remains to be determined. Interestingly, PKR and Dicer are functionally interconnected. PKR interacts with Dicer’s helicase domain to prevent aberrant NF-κB pathway activation^52,53^. Dicer has been proposed to regulate PKR activation in mESCs^78^ and both share cofactors, including TRBP and PACT, which modulate their activity^79^. Whether these interactions influence NoV B2’s suppressive functions warrants further investigation.

The comparison with other VSRs provides additional mechanistic insights. VACV-E3L also enhanced saRNA activity in our system, consistent with a previous report^62^, yet less effectively than NoV B2. This suggests that, beyond its direct dsRNA-binding activity that shields dsRNA from PKR recognition, the ability of NoV B2 to associate with the alphavirus replication complex contributes to its enhanced efficacy. Alphaviruses form replication organelles (spherules) at the plasma membranes, where nsP1, assembling into a 12-mer ring, anchors the vRC and nsP4 synthesises the (–)-strand viral antigenome, the primary source of viral dsRNA^65,80,81^. The N-terminal domain of nsP1 harbours methyltransferase (MTase) and guanylyltransferase (GTase) activities required for viral RNA capping. Nodaviruses encode a multifunctional viral replicase, protein A, whose N-terminal domains share striking structural homology with alphavirus nsP1 in its N-terminal MTase/GTase and central channel domains^64,68^ and, like nsP1, multimerises into 12-mer ring-like structures, albeit at the outer mitochondrial membrane (OMM) rather than the plasma membrane^82,83^. This conservation of crown architecture across these distinct virus families, together with the previously reported association between FHV B2 and protein A^32^ led us to hypothesise that NoV B2 might similarly interact with components of the alphavirus vRC. Consistent with this, confocal microscopy showed that NoV B2 localises at the cell periphery adjacent but not overlapping with nsP1 and beneath dsRNA foci. Our data further show that NoV B2 specifically interacts with nsP2 likely via its MTase-like domain, a structurally defined but functionally poorly characterised domain found across alphaviruses^84^. Since nsP2 is located on the cytoplasmic side of the spherules, this interaction may facilitate the positioning of NoV B2 in close proximity to the alphavirus replication machinery, potentially enabling it to shield replication-derived dsRNA from the PKR pathway. We note that the interaction between NoV B2 and nsP2 was demonstrated specifically for CHIKV nsP2. While NoV B2 similarly enhanced VEEV-based saRNA expression in a PKR-dependent manner and altered VEEV dsRNA distribution, we did not test whether NoV B2 interacts with VEEV nsP2, and whether this interaction generalises across alphavirus backbones remains to be established. Similarly, the precise identity of the dsRNA species bound by B2, and whether this interaction directly drives inhibition of PKR-mediated eIF2α phosphorylation, remain to be determined. The enrichment of dsRNA to the cell periphery, observed in NoV B2^WT^-expressing cells, may stabilise dsRNA within spherules and limit its accessibility to PKR and its main substrates, eIF2α (Fig. S8). Whether analogous mechanisms operate during natural NoV or FHV infections remains to be addressed, although spatial redistribution of dsRNA at the mitochondrial membrane may be technically challenging to detect given the dispersed cytoplasmic distribution of this organelle.

Given the promise of saRNA vaccines as a low-dose vaccination platform in humans, our findings that NoV B2 enhances saRNA expression in somatic cells without disrupting innate immune signalling reveal a strategy to boost saRNA efficacy by co-opting a viral dsRNA-binding protein to alleviate PKR-mediated translational shutdown while preserving self-adjuvanticity. These mechanistic insights provide a framework for optimising saRNAs with improved transgene expression. Evaluating whether this strategy translates into enhanced vaccine immunogenicity in vivo and assessing whether NoV B2 expressed from alphavirus saRNAs causes any overt toxicity in animal models will be the next critical steps. As a foreign viral protein, NoV B2 may itself be immunogenic in vivo. Whether anti-B2 immune responses develop upon vaccination and whether these affect antigen expression kinetics or limit repeat dosing remains to be determined. Our characterisation of innate immune activation was also limited to a defined panel of ISGs and cytokines in vitro. Broader effects of NoV B2 on innate immune signalling in vivo, as well as any consequences of sustained antigen expression, remain to be evaluated. In this regard, our findings that saRNAs induce a global translation shutdown and that *cis*-expression of NoV B2 mitigates this response are particularly relevant. Notably, transgene expression from saRNA delivered via lipid nanoparticles in vivo can persist for 4–8 weeks^72,85^, potentially leading to sustained PKR activation and a prolonged integrated stress response that could promote cell death^86^. The *cis*-expression of NoV B2 from saRNA may therefore not only enhance antigen expression but also preserve cell viability by reducing this global translational shutdown. While prolonged cell viability and sustained transgene expression could, in principle, influence immune activation kinetics in vivo, such effects are likely to be context- and dose-dependent. Importantly, we did not observe increased ISG induction upon B2-mediated enhancement of transgene expression under our experimental conditions, suggesting that enhanced saRNA expression does not exacerbate the IFN-I response. Moreover, since saRNA delivery of reprogramming factors was shown to efficiently generate human iPSCs, NoV B2 may also improve the performance of saRNA-based regenerative therapies^87^. Altogether, our findings define an engineering strategy to enhance saRNA-driven protein expression while preserving self-adjuvanticity, providing a foundation for future in vivo evaluation in vaccine and therapeutic contexts. Future studies should assess whether saRNA potency can be further enhanced by combining the *cis*-expression of NoV B2 with other reported optimisations such as complete substitution of saRNA with modified nucleotides^88^ and/or the co-expression of other viral proteins known to enhance saRNA activity^17^.

## Methods

### Cloning

CHIKV^ERR^ saRNA plasmid was based on saRNA vector of CHIKV LR2006OPY1 strain^89^, a mutation _674_ALT_676_ to _674_ERR_676_ eliminating cytotoxic properties of nsP2 protein^43^was introduced using site-directed mutagenesis. VEEV saRNA plasmid was generated using SP6-VEE-IRES-Puro (Addgene #58971) by introduction of two point mutations in the non-structural region and replacement of IRES-Puro region a multiple cloning site downstream of the subgenomic promoter. Different constructs were generated using standard molecular cloning techniques. Transgene inserts were amplified via PCR/overlap PCR using Q5® High-Fidelity DNA Polymerase (M0491, New England Biolabs), and cloned into CHIKV^ERR^ or VEEV vector using ApaI/PacI restriction sites (New England Biolabs). The GFP sequence present in the engineered constructs is from mGreenLantern (Addgene #161912)^90^. Codon optimized prefusion SARS-CoV-2 Spike-encoding sequence was obtained as synthetic DNA [Genewiz]. Hemaglutinin (HA)-encoding sequence from Influenza virus A PR8 strain was amplified by PCR from a plasmid encoding the wt HA sequence kindly provided by Professor Wendy Barclay (Imperial College London, London, UK)^91^. Backbone plasmids were dephosphorylated after digestion [Antarctic Phosphatase, M0289, New England Biolabs]. Ligation reactions were performed using T4 DNA Ligase [M0202, New England Biolabs] for 16 h at 16 °C, and bacterial transformation was carried out using NEB 5 alpha competent *E. coli* cells, a derivative of DH5α [C2987U, New England Biolabs] by heat-shock. Clones were screened by Sanger sequencing and the whole plasmid sequences were verified by Nanopore sequencing technology (Source BioScience). Sequences encoding PKR, B2, and CHIKV-nsPs were amplified by classical PCR/overlap PCR with the corresponding tags added within the forward primer, cloned into pCAGG vector and verified by Sanger sequencing and whole plasmid sequencing. Sequences of all plasmid and primers used for cloning are available upon request.

### saRNA in vitro transcription

saRNAs were synthesized using mMACHINE™ SP6 Transcription Kit (AM1340, ThermoFisher Scientific) from linearized plasmid DNA template. CHIKV^ERR^ and VEEV plasmids were linearised using NotI (R0189S, New England Biolabs), and MluI (R3198S, New England Biolabs), respectively. Linearised plasmids were run on agarose electrophoresis and gel purified (Monarch DNA Gel extraction kit, New England Biolabs). The in vitro transcription reaction was carried out at 37 °C for 2 h with 1 µg of template DNA followed by DNase I treatment to remove residual DNA. RNA was purified using silica column-based purification (74106, RNeasy Kit, Qiagen) and quantified using a Nanodrop spectrophotometer (DS-11 DeNovix). RNA integrity was assessed by Bioanalyzer (Agilent 2100).

### Cell culture and cell lines

HeLa (ATCC CCL-2), HeLa *PKR*⁻/⁻, HEK293 (ATCC CRL-1573), and HEK293T (ATCC CRL-3216) and BHK-21 (ATCC CCL-10) cells were maintained in Dulbecco’s Modified Eagle’s Medium (DMEM; 31966, Gibco) supplemented with 10% heat-inactivated fetal bovine serum (FBS, FB-1001, Biosera) and 100 U ml^−1^ penicillin/streptomycin (15140, Gibco) at 37 °C in 5% CO₂. THP-1-Dual™ (thpd-nfis, InvivoGen) and THP-1-Dual™ KO-IFNAR2 cells (InvivoGen) were cultured in Roswell Park Memorial Institute 1640 Medium (RPMI, 21875, Gibco) containing 10% FBS, and100 U ml^−1^ penicillin/streptomycin. E14 mESCs (CRL-1821) were cultured in DMEM supplemented with 10% embryonic stem cell qualified fetal calf serum (FCS, Gibco), 2 mM non-essential amino acids (25-025-CIR, Gibco), 2 mM sodium pyruvate (S8636, Sigma-Aldrich), 2mM L-glutamine (25030, Gibco), 100 U ml^−1^ penicillin/streptomycin, 0.2% β-mercaptoethanol (31350, Gibco), and 1000 U ml^−1^ ESGRO recombinant mouse leukaemia inhibitory factor (ESG1107, Sigma-Aldrich), at 37 °C with 5% CO₂ in gelatine-coated plates. Cells were passaged using trypsin upon reaching 60% confluency. HEK293T *Dicer*^-/-^ and HEK293T *Dicer*^-/-^*PKR*^-/-^ cells were kindly provided by Prof. Bryan R. Cullen (Duke University Medical Center, Durham, USA)^50,51^. HeLa *PKR*^-/-^ cells were kindly provided by Prof. Adam P. Geballe (Fred Hutchinson Cancer Center, Seattle, USA)^92^. HEK293 RIG-I^-/-^ MDA5^-/-^ (double KO, DKO) were a kind gift from Dr Annemarthe Van der Veen (Leiden University Medical Center, Netherlands)^93^.

### IFN-I and NF-κB reporter assay with THP-1-Dual™ cells

THP-1 cells were transfected with saRNAs and, 24 h later, supernatants (20 μL) were transferred to a white (opaque) 96-well plate (3922, Corning). Secreted Lucia luciferase activity was then measured using the *Renilla* Luciferase Assay System (E2820, Promega) following the manufacturer’s instructions. Luminescence was recorded using a FLUOstar Omega plate reader (BMG LABTECH). Secreted embryonic alkaline phosphatase (SEAP) activity in culture supernatants was quantified using the QUANTI-Blue™ Solution (rep-qbs, InvivoGen). Supernatants were incubated with QUANTI-Blue™ at 37 °C for 1 h, and absorbance was measured at 620–655 nm using a spectrophotometer.

### saRNA transfection and drug treatment

Cells were collected, counted, and resuspended in serum-free Opti-MEM™ before transfection. Transfection complexes were prepared by incubating 200 ng of saRNA per 100,000 cells or 1 µg per 500,000 cells with Lipofectamine™ MessengerMAX™ (LMRNA001, Invitrogen) in Opti-MEM™ (31985070, Gibco) for 10–15 min at room temperature. Complexes were added directly to the cell suspension, followed by 30 min incubation at 37 °C, 5% CO₂. After transfection, cells were transferred to the appropriate well format with complete medium or, where indicated, complete medium containing PKR inhibitor C16 (527450, Sigma-Aldrich), or 500 nM of JAK inhibitor Ruxolitinib (tlrl-rux, Invivogen), or 500 UI mL^−1^ of universal type I IFN (human IFN alpah A/D) (11200, PBL assay Science).

### Virus-replicon particle (VRP) production

saRNAs of CHIKV^ERR^-GFP-B2^WT^, CHIKV^ERR^-GFP-B2^R59Q^, and CHIKV^ERR^-GFP-STOP were generated using the CHIKV split-helper system as previously described^94^. Briefly, linearized templates of saRNA and helper RNAs were subjected to in vitro transcription. For recovery of VRPs, 1 µg of each saRNA and helper RNA was co-electroporated into 1 × 10⁶ BHK-21 cells using a Gene Pulser Xcell system (Bio-Rad). Cells were then seeded into 25 cm² flasks and incubated at 28 °C for 48 h. Supernatants were collected and clarified by centrifugation at 4000 × g for 30 min at 4 °C to remove detached cells and debris. The clarified supernatants were subsequently passed through a 0.45 µm filter and stored at −80 °C. For titration, BHK-21 cells were infected with serial dilutions of VRP stocks. Infected cells were identified based on GFP fluorescence and quantified by cell counting.

### Fluorescence-activated cell sorting (FACS) and flow cytometry analysis

Cells were harvested by trypsinisation, washed with PBS containing 2% FCS and resuspended in PBS-4%-PFA (40-7401-05, Severn Biotech Ltd.), washed and analysed using an BD LSR II Flow Cytometer acquiring a sample size of 10,000 cells. For samples that required intracellular staining, cells were permeabilized (BD Perm/Wash™, 554723, BD Biosciences), blocked and stained with either a) anti-Flag, b) anti-STrEP, c) anti phospho-eIF2α antibodies for 2 h at room temperature. Samples were then incubated with secondary antibody anti-mouse AF647 (A-21239, Invitrogen) for 2 h at room temperature in the dark. Cells were washed 3 times with PBS and resuspended in 200 µL of FACS buffer (PBS, 2% FCS, 2 mM EDTA). Data analysis was performed with FlowJo (TreeStar).

### RNA extraction and quantification

Total RNA was extracted using the RNeasy Mini Kit (Qiagen) or TRIzol reagent (Invitrogen) following manufacturer’s protocols. Column purification was followed by DNase-treatment according to the manufacturer’s instructions (Qiagen, 79254). RNA concentration and purity were assessed using a Nanodrop spectrophotometer (DS-11 DeNovix) and integrity was confirmed using a Bioanalyzer (Agilent 2100) and agarose gel electrophoresis. Reverse transcription was performed using the SuperScript™ III First-Strand Synthesis System for RT-PCR (18080-051, Invitrogen) with random hexamers or oligo-dT primers. qPCR reactions were set up using SYBR Green Master Mix (4309155, Applied Biosystems) and amplification was performed on a LightCycler 480 System (Roche). Relative expression levels were determined using the ΔΔCt method, normalizing to GAPDH. In a subset of experiments, HEK293T cells were transfected with CHIKV-GFP-B2^WT^, CHIKV-GFP-B2^R59Q^ and CHIKV-GFP-STOP saRNAs and then GFP-positive cells were isolated by FACS prior to RNA extraction and qRT-PCR analysis. To control for variability in total RNA delivery per well, the GFP-negative population from the same wells was also sorted and both fractions were incorporated into the ΔΔCt calculation performed as follows:

$${2}^{-{\Delta} {\Delta} {{\rm{Ct}}}}={2}^{-[\left.\left({{\rm{Ct}}}\left({{\rm{saRNA}}}\right)-{{\rm{Ct}}}\left(GAPDH\right)\right)\, {{\rm{in}}}{{\bf{GFP}}}\, {{\bf{positive}}} \,{{\bf{cells}}}\right)]-[\left.\left({{\rm{Ct}}}\left({{\rm{saRNA}}}\right)-{{\rm{Ct}}}\left(GAPDH\right)\right)\,{{\rm{in}}}\, {{\bf{GFP}}}\, {{\bf{negative}}}\,{{\bf{cells}}}\right)]}$$

Primer sequences are listed in Supplementary Table S2.

### Small RNA deep sequencing after cell sorting

50,000 GFP positive E14 cells were sorted using a BD FACSAria (BD Biosciences) and collected in FCS-containing medium. Sorted cells were centrifuged and lysed in TRIzol (10296010, Ambion). RNA was extracted using the phenol/chloroform protocol. Experiments were done on biological duplicates. Small RNA deep sequencing was carried out by Fasteris (Genesupport SA), Geneva, Switzerland. Briefly, RNA processing and library preparation were done in parallel. Library preparation for small RNA sequencing was performed using the QiaSeq Small RNA Library Prep Kit, following the manufacturer’s protocol. Sequencing was performed on an Illumina NextSeq sequencer at 1 × 75 bp read length The adapter sequences were trimmed from the raw reads using the UMI-tools. Inserts of 15 to 35 bases were selected and mapped with BWA to mouse miRNAs (miRbase-r22_1). Mapped reads counts were used to quantify miRNA expression. Differential expression analysis was conducted using DESeq2.

### Confocal microscopy and image analysis

Cells were seeded onto an ibidi 8-well (80826, ibidi GmbH) chambered coverslip and fixed with 4% paraformaldehyde for 30 min at room temperature. Cells were washed with PBS and then permeabilized using 0.5% Triton X-100 in PBS for 30 min. The coverslip was then blocked for 2 h at room temperature with Maxblock (15252, Active Motif) and subsequently incubated with primary antibodies in 0.1% Triton X-100 in PBS overnight at 4 °C. Antibody references are provided in Supplementary Table S1. Primary antibodies include mouse anti-strep (1/500), rabbit anti-strep (1/500), rabbit anti-CHIKV-nsP1 (1/200), rabbit anti-CHIKV-nsP2 (1/200), mouse anti-dsRNA J2 (1/200) and rabbit anti-dsRNA J2 (1/200) and rabbit anti-Phospho-EiF2alpha (1/200). Before incubation with secondary antibodies (goat anti-rabbit IgG Alexa Fluor™ 405 (1/500), goat anti-mouse IgG Alexa Fluor™ 405 (1/500), goat anti-mouse IgG1 Alexa Fluor™ 568 (1/500), goat Anti-mouse IgG, Alexa Fluor™ 647 (1/500) or goat Anti-rabbit IgG, Alexa Fluor™ 647 (1/500) for 2 h at room temperature, samples were washed 3 times for 5 min with 0.1% Triton X-100 in PBS. F-actin was labelled using Alexa Fluor™ 594 Phalloidin (1/5000). The nuclei were stained with either DAPI (1/1000, 564907, BD Pharmingen) or NucSpot 568/580 (1/5000, 41036, Biotium). In cases where the nuclei were stained with DAPI, the dye was included in the secondary antibody mix. When an Alexa Fluor 405-conjugated secondary antibody was used, the staining protocol was adapted to include the NucSpot 568/580 which was applied after secondary antibody staining followed by incubation for 5 min at room temperature. Next, samples were washed 3 times for 5 min with PBS and then preserved in excess of PBS until imaging. Images were acquired using a Zeiss LSM 880 Airyscan confocal microscope or Nikon AXR super-resolution confocal microscope. Images taken with the LMS880 were processed using the ‘Airyscan processing’ package in the Zen black (Zeiss) software before further image analysis. Analysis was done using Arivis (Zeiss) and Image J (Fiji). Nikon AXR images were acquired with the resonant scanner and denoised using the denoise.ai platform provided in the Nikon Elements software.

Image analysis of dsRNA aggregates was done in Arivis (Zeiss). To that end all images were converted using the plug in the Arivis software (Arivis SIS converter). The analysis consisted of 4 steps: identifying the cells, identifying the dsRNA aggregates, allocating dsRNA to the cells and measuring distance to its centre. Transfected cells were identified using the GFP signal. This signal was first denoised (method; median, diameter; 0.5 μm), and subsequently filtered using intensity (method; simple, object type; bright, threshold depending on the experiment) and size (10-5000 μm^2^). The dsRNA aggregates were identified using the Blob finder (diameter; 0.49 μm, Probability threshold; 14%, Split sensitivity 90%) and then filtered on size (voxel size of 35). Allocating the dsRNA to the cells was done using the compartmentalization tool. All dsRNA aggregates within the GFP, on the border of the GFP or within 0.49 μm of the border were included. Finally, distances of the dsRNA aggregates to the centre of the GFP were calculated (Distance tool; parent, centre of geometry).

### Nascent protein synthesis assay

HeLa cells were transfected with VEEV-GFP-saRNA and, after 20 h, were washed with PBS and incubated 1 h in methionine-free media (21013, Gibco) supplemented with L-cystein (C6852, Sigma) to starve the cells prior to priming. Cells were then incubated with L-homopropargylglycine (HPG) to label newly synthesized proteins using the Click-iT™ HPG Alexa Fluor™ 594 Protein Synthesis Assay Kit (C10429, Invitrogen) according to the manufacturer’s instructions. Cells were then trypsinised, fixed with PBS containing 4% paraformaldehyde and HPG incorporation was analysed by flow cytometry.

### Protein co-immunoprecipitation

4 × 10⁶ HEK293T cells were plated in 100 mm dishes and transfected the following day using Lipofectamine 2000 (11668-019, ThermoFisher) with plasmids encoding N-terminal 1xSTrEP -B2, -mCherry, or -B2-R59Q, alongside plasmids encoding CHIKV replication complex components (i.e., nsP1, nsP2^ERR^, nsP3, nsP4) together or independently. Transfections were performed according to the manufacturer’s recommendations and transfected cells were subsequently incubated for 24 h at 37 °C, 5% CO₂. Cells were then lysed in 1 mL of MOPS lysis buffer (20 mM MOPS-KOH pH 7.4, 150 mM KCl, 0.5% NP-40, 2 mM β-mercaptoethanol) supplemented with Complete Mini EDTA-free protease inhibitor cocktail (11836170001, Roche) for 30 min on ice, followed by centrifugation (12,000 × *g*, 15 min, 4 °C). 50 µL of lysate was collected for total protein samples, remaining lysate was incubated with Strep-Tactin beads (2-1613-002, IBA Lifesciences) overnight at 4 °C in a spinning wheel. Beads were washed 4 times with lysis buffer and eluted in 2 × 50 µl of 1 × Buffer E (2-1000-025, IBA Lifesciences). Total and eluted protein samples were resolved on a Tris-Glycin SDS-polyacrylamide gel and analysed by western blotting.

### Western blotting

Cells were lysed in RIPA buffer (89900, Thermo Scientific) supplemented with protease (11836170001, Roche) and phosphatase inhibitors (78446, Thermo Scientific). Proteins were resolved on 7.5% or 4-15% or 4-20% Mini-PROTEAN TGX gels (4561023, 4561095, 4561086, BIORAD) and transferred by electroblotting to PVDF membranes (Millipore). Membranes were blocked in either TBS with 0.1% Tween-20 and 5% non-fat milk or 5% milk/TBST (except for detection of phosphorylated residues, at this case 5% BSA was used), incubated with primary antibodies (Supplementary Table S2) overnight at 4 °C, followed by incubation with HRP-conjugated secondary antibodies (1:10000). Detection was performed using ECL Western blotting reagents (RPN2232, Cytiva) or SuperSignal West Femto maximum sensitivity substrate (34095, ThermoFisher Scientific). Anti-B2 antibody was kindly provided by Prof. Shou-Wei Ding (University of California, Riverside, USA).

### Dot-blot

HEK293T cells (3.5 × 10⁶) were seeded in 100 mm dishes and transfected the following day with 10 µg of saRNA encoding GFP-STrEP-B2wt or GFP-STrEP-B2-R59Q. Cells were harvested 24 h post-transfection and lysed in MOPS-KOH lysis buffer (20 mM MOPS-KOH pH 7.4, 150 mM KCl, 0.5% NP-40, 2 mM β-mercaptoethanol), supplemented with RNase inhibitor (RNAsin, 12.5 U ml⁻¹, N2511, Promega).

Strep-Tactin beads (2-1613-002, IBA Lifesciences) were washed, pre-cleared with lysates from non-transfected HEK293T cells (1 h, 4 °C), and incubated with clarified lysates for 2 h at 4 °C. Beads were washed three times and bound complexes were eluted in 100 µl of biotin-containing elution buffer (1 × Buffer E, 2-1000-025, IBA Lifesciences). Eluates were split for protein analysis by Western blot and RNA extraction by phenol–chloroform.

Purified RNA was adjusted to 200 µl with RNase-free water, supplemented with RNA loading dye, and directly applied onto a positively charged nylon membrane (BrightStar-Plus, Ambion) using a Bio-Dot Microfiltration Apparatus (1706545, Bio-Rad). RNA was UV-crosslinked (120,000 µJ cm⁻², 254 nm), and membranes were blocked in TBS-T containing 5% milk, incubated with anti-dsRNA antibody (J2, 1:1000) overnight at 4 °C, followed by HRP-conjugated secondary antibody (1:10,000). Signal was detected by ECL.

### Statistics and reproducibility

No statistical method was used to predetermine sample size. Sample sizes were chosen based on standard practice in the field and are indicated in the figure legends. No data were excluded from the analyses. The experiments were not randomised. The investigators were not blinded to allocation during experiments and outcome assessment. Statistical analyses were performed using GraphPad Prism. Unless otherwise stated, data are presented as mean ± standard deviation of at least three independent biological replicates with each data point representing the mean of technical replicates. Statistical comparisons were performed using two-way ANOVA with Šídák’s, Dunnett’s or Tukey’s multiple comparisons test, two-way ANOVA with Fisher’s LSD test or two-tailed ratio paired *t* test, as indicated in the figure legends. A *p* value of less than 0.05 was considered statistically significant (*P < 0.05; **P < 0.01; ***P < 0.001; ****P < 0.0001). Exact *p* values are provided in the Source Data file. The absence of a significance indicator in a figure panel indicates that the comparison was not statistically significant or that statistical testing was not appropriate for that data type. Representative data are shown for western blots, confocal microscopy images, flow cytometry contour plots and histograms. The number of independent experiments from which representative data are shown is indicated in the figure legends.

### Reporting summary

Further information on research design is available in the Nature Portfolio Reporting Summary linked to this article.

## Supplementary information

Supplementary Information

Reporting Summary

Transparent Peer Review file

## Source data

Source Data

## Data Availability

The small RNA deep sequencing data generated in this study have been deposited in NCBI’s Gene Expression Omnibus (GEO) and are accessible through GEO Series accession GSE341822. Source data are provided with this paper.
