# Supplementary material for: Tuning intracellular immunity by Nodamura virus B2 protein enhances self-amplifying RNA activity
Source: Nat Commun. 2026 Sep 28;17:10028. doi: 10.1038/s41467-026-77816-2 (PMC13620136; doi:10.1038/s41467-026-77816-2)
Supplement: Supplementary file 1 [file 41467_2026_77816_MOESM1_ESM.pdf]

A

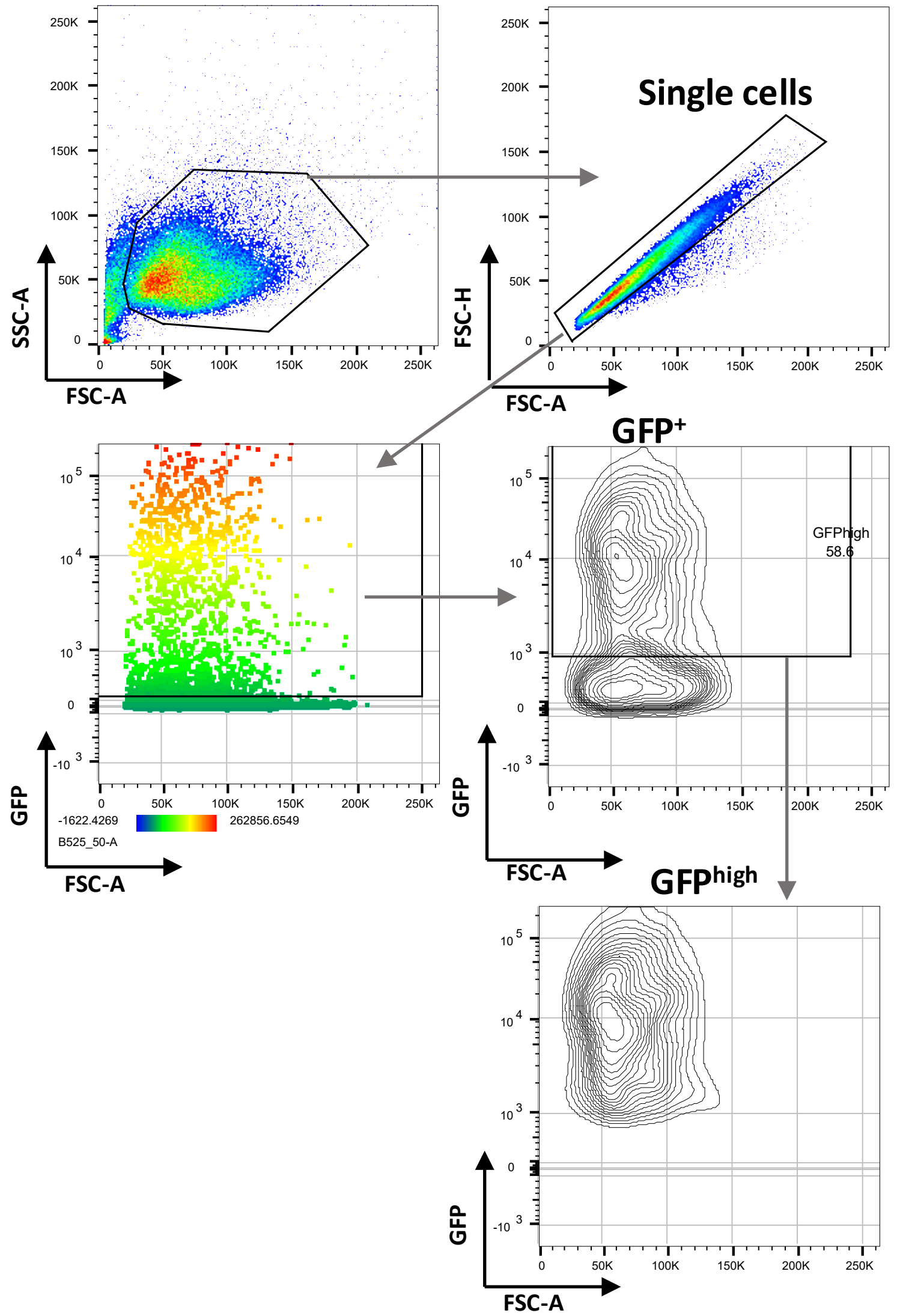

B

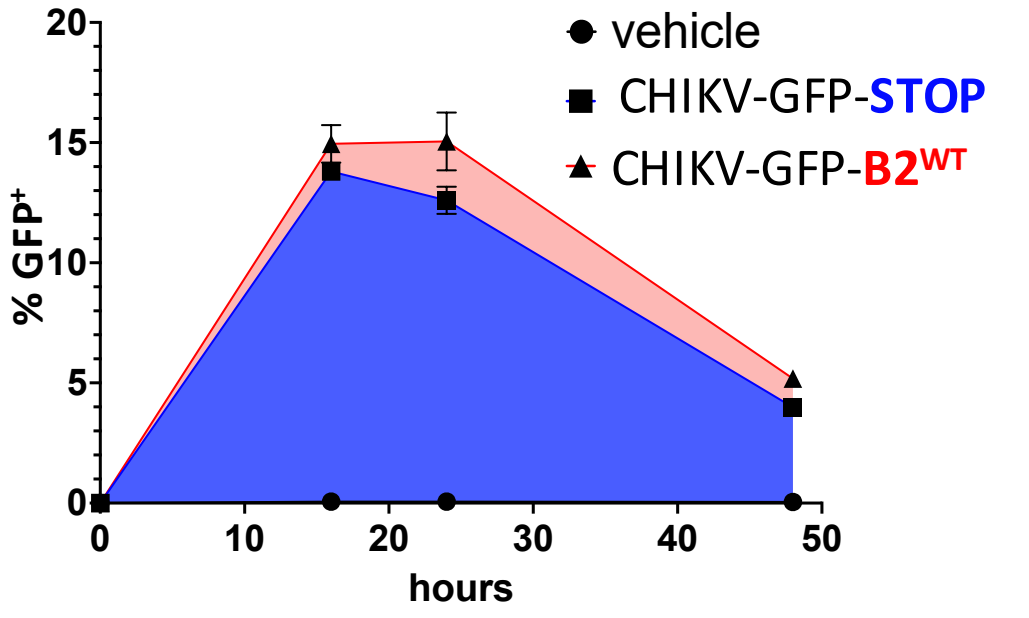

C

experiment	total #reads	inserts 15-35 (N)	inserts 15-35nt (%)	Reads 15-35nt aligning with vector (N)	Reads 15-35nt aligning with vector (%)	Reads 20-25nt aligned with vector (N)	Reads 20-25nt aligned with vector (%)
$B2^{WT}$-(1)	77861494	12176247	15.6	3251876.00	26.7%	225462	6.9%
$B2^{WT}$-(2)	73566566	12121040	16.5	3479414.00	28.7%	204685	5.9%
$B2^{R59Q}$-(1)	82773919	15105448	18.2	3925262.00	25.9%	499796	12.7%
$B2^{R59Q}$-(2)	84291020	15088127	17.9	3260437.00	21.6%	551692	16.9%

Figure S1

Figure S1. Flow cytometry gating strategy for saRNA experiments and small RNA sequencing summary in E14 mESCs.

(A) Gating strategy for the flow cytometry assay defining GFP^{high} cells. E14 mESCs were gated based on side scatter area (SSC-A) versus forward scatter area (FSC-A). Singlets were identified using FSC-Height (FSC-H) versus FSC-A to exclude doublets. Single cells were then analysed for GFP expression. Cells with MFI greater than 10^3 were classified as GFP^{high}. **(B)** Percentage of GFP-positive (GFP^+) cells in E14 mESCs transfected with CHIKV-GFP-STOP (blue) , CHIKV-GFP-$B2^{WT}$ (red) saRNA or transfection reagent alone (vehicle) at 16, 24 and 48 hours post-transfection. Data are mean ± s.d. from a representative of two independent experiments. **(C)** Summary of small RNA deep sequencing reads showing the number of raw and mapped reads obtained for each sample. Mapping was performed against each corresponding CHIKV-based saRNA genome.

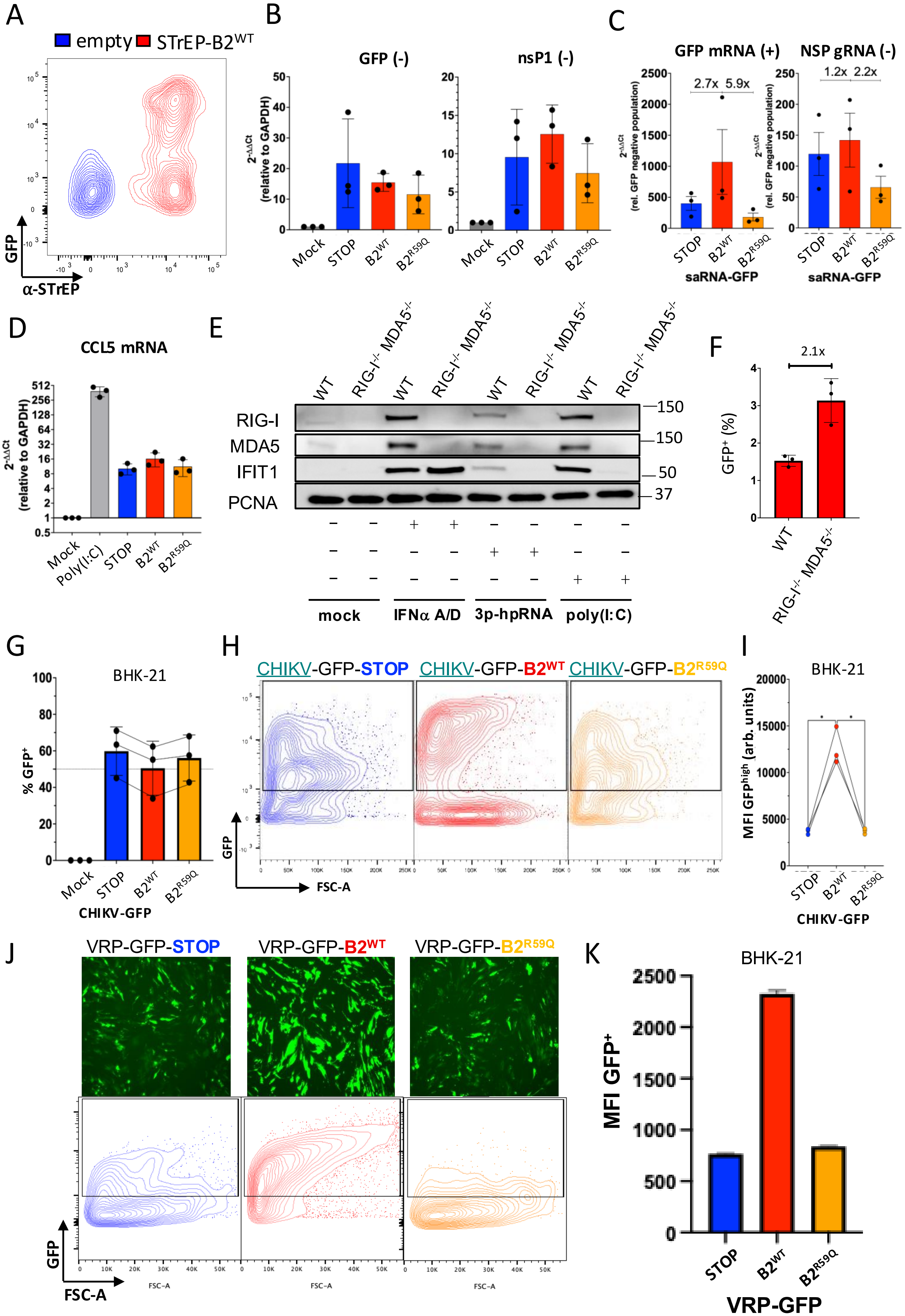

A
empty STrEP-B2WT
GFP
α-STrEP
B
GFP (-)
nsP1 (-)
2-ΔΔCt (relative to GAPDH)
Mock STOP B2WT B2R59Q
C
GFP mRNA (+)
NSP gRNA (-)
2.7x 5.9x
1.2x 2.2x
2-ΔΔCt (rel. GFP negative population)
STOP B2WT B2R59Q
saRNA-GFP
D
CCL5 mRNA
2-ΔΔCt (relative to GAPDH)
Mock Poly(I:C) STOP B2WT B2R59Q
E
WT RIG-I-/- MDA5-/-
RIG-I
MDA5
IFIT1
PCNA
150
150
50
37
mock
IFNα A/D
3p-hpRNA
poly(I:C)
F
2.1x
GFP+ (%)
WT RIG-I-/- MDA5-/-
G
BHK-21
% GFP+
Mock STOP B2WT B2R59Q
CHIKV-GFP
H
CHIKV-GFP-STOP
CHIKV-GFP-B2WT
CHIKV-GFP-B2R59Q
GFP
FSC-A
I
BHK-21
MFI GFPhigh (arb. units)
STOP B2WT B2R59Q
CHIKV-GFP
J
VRP-GFP-STOP
VRP-GFP-B2WT
VRP-GFP-B2R59Q
GFP
FSC-A
K
BHK-21
MFI GFP+
STOP B2WT B2R59Q
VRP-GFP

Figure S2

Figure S2. Nov B2 enhances saRNA expression and innate immune activation across multiple cell types and delivery systems.

(A) HeLa cells were co-transfected with CHIKV-GFP-STOP saRNA and either a control plasmid (blue) or a pCAGG construct encoding 1xSTrEP-tagged $B2^{WT}$ (red). 24 hours post-transfection, intracellular B2 expression was measured by flow cytometry using anti-STrEP antibody. Cells were gated based on STrEP signal and GFP fluorescence. **(B)** HeLa cells were transfected with CHIKV-GFP-STOP (blue), CHIKV-GFP-$B2^{WT}$ (red) or CHIKV-GFP-$B2^{R59Q}$ (yellow) saRNA, analysed 24 hours post-transfection by RT-qPCR for CHIKV saRNA negative (-)- strand RNA using primers targeting GFP (left) or nsP1 (right). **(C)** HeLa cells transfected as in (B) and, 24 hours post-transfection, GFP^{high} cells were isolated by FACS prior to RNA extraction and RT-qPCR analysis to assess the levels of positive (+)- strand GFP mRNA (left) or CHIKV negative (-)-strand RNA levels using primers targeting nsP1 region (right). **(D)** HeLa cells transfected with CHIKV-GFP-STOP (blue), -$B2^{WT}$ (red), -$B2^{R59Q}$ (yellow) saRNA or poly(I:C) (100ng, 24 hours). CCL5 mRNA analysed by RT-qPCR **(E)** Western blot of HEK293 WT or RIG-$I^{-/-}$ $MDA5^{-/-}$ cells treated with recombinant universal IFN-I (IFNα-A/D) or transfected with 5′-triphosphate hairpin RNA (3p-hpRNA) or poly (I:C). **(F)** Percentage of GFP^{+} HEK293 WT or RIG-$I^{-/-}$ $MDA5^{-/-}$ cells transfected with CHIKV-GFP-$B2^{WT}$ and analysed by flow cytometry. **(G)** Percentage of GFP^{+} BHK-21 cells 24 hours post-transfection with CHIKV-GFP-STOP (Blue), CHIKV-GFP-STOP-$B2^{WT}$ (red) or CHIKV-GFP-STOP-$B2^{R59Q}$ (orange) analysed by flow cytometry. Data are mean ± s.d., n=3 independent experiments **(H)** Representative contour plots of GFP^{+} BHK-21 cells transfected with the CHIKV-GFP-STOP (Blue), -$B2^{WT}$ (red) or -$B2^{R59Q}$ (orange) saRNA. **(I)** GFP^{+} MFI in BHK-21 cells transfected with the indicated saRNAs. Data are means ± s.d., n=3 independent experiments**.** **(J)** CHIKV-GFP-STOP, -$B2^{WT}$ or -$B2^{R59Q}$ saRNAs packaged into viral replicon particles (VRPs) and used to transduce BHK-21 cells at a multiplicity of infection (MOI) of 10 (shown in the panels as VRP-GFP-STOP, VRP-GFP-$B2^{WT}$, VRP-GFP $B2^{R59Q}$). Representative fluorescence microscopy images 24 hours post-transduction with corresponding contour plots of GFP+ cells displayed below each image. (K) GFP+ MFI by flow cytometry in BHK-21 cells following VRP-mediated delivery of the indicated saRNA 24 hours post-transduction. Data is a representative of 3 independent experiments.

Statistical analysis was performed using two-way ANOVA followed by Holm-Šídák 's multiple comparisons test (I). *$P < 0.05$; ns, not significant. arb. units, arbitrary units. Exact p-values and source data are provided as a Source Data file.

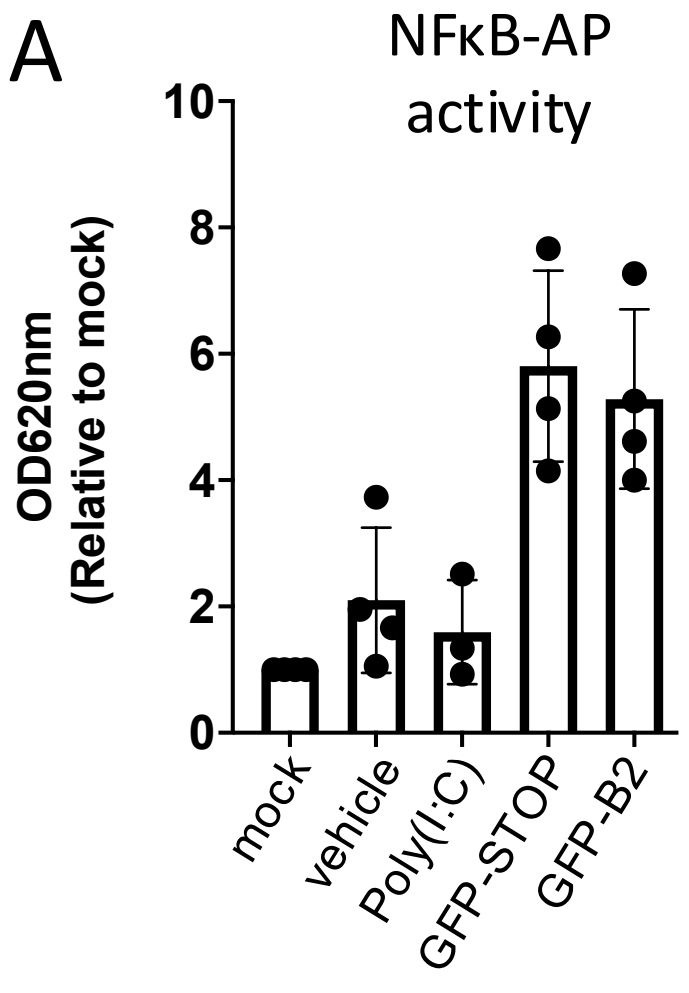

Figure S3. NF-κB activation by saRNA in THP-1-2R cells.

(A) THP-1-2R WT cells were transfected with CHIKV-GFP-STOP or -B2^{WT} saRNA or poly(I:C) or with the transfection reagent alone (vehicle). NF-κB activation was quantified by measuring secreted alkaline phosphatase (SEAP) activity in the supernatant. Data are mean ± s.d., n=4 independent experiments, each point represents the average of technical replicates. Source data are provided as a Source Data file.

Figure S3

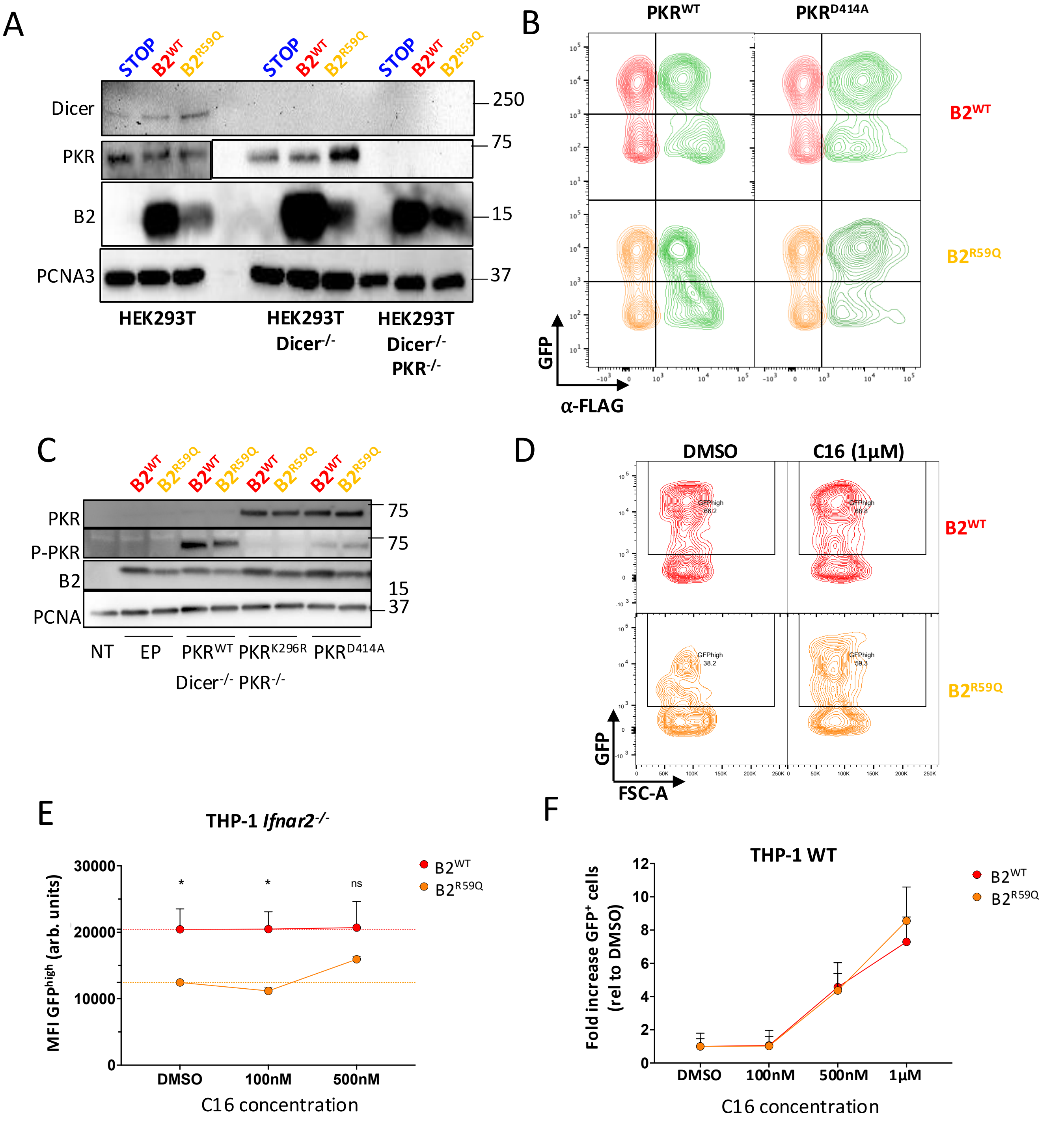

A
STOP B2WT B2R59Q STOP B2WT B2R59Q STOP B2WT B2R59Q
Dicer
PKR
B2
PCNA3
250
75
15
37
HEK293T
HEK293T Dicer-/-
HEK293T Dicer-/- PKR-/-
B
PKRWT
PKRD414A
B2WT
B2R59Q
GFP
α-FLAG
C
B2WT B2R59Q B2WT B2R59Q B2WT B2R59Q B2WT B2R59Q
PKR
P-PKR
B2
PCNA
75
75
15
37
NT
EP
PKRWT
PKRK296R
PKRD414A
Dicer-/- PKR-/-
D
DMSO
C16 (1µM)
B2WT
B2R59Q
GFP
FSC-A
E
THP-1 Ifnar2-/-
MFI GFPhigh (arb. units)
30000
20000
10000
0
*
*
ns
DMSO
100nM
500nM
C16 concentration
B2WT
B2R59Q
F
THP-1 WT
Fold increase GFP+ cells (rel to DMSO)
12
10
8
6
4
2
0
DMSO
100nM
500nM
1µM
C16 concentration
B2WT
B2R59Q

Figure S4

Figure S4. PKR expression and inhibition experiments supporting the role of NoV B2 in counteracting PKR-mediated saRNA restriction.

(A) Western blot analysis of HEK293T-WT, -*Dicer*$^{-/-}$ and *Dicer*$^{-/-}$*PKR*$^{-/-}$ cells 24 hours post-transfection with corresponding CHIKV-GFP-STOP (blue), -B2^{WT} (red) or -B2^{R59Q} (orange) saRNAs. **(B)** Representative contour plots of HEK293T-*Dicer*$^{-/-}$*PKR*$^{-/-}$ cells transfected first with empty plasmid (EP, red/ orange) or FLAG-PKR^{WT} or FLAG-PKR^{D414A} (both shown in green). After 24 hours, cells were transfected with CHIKV-GFP-B2^{WT} (top row, red/green) or -B2^{R59Q} (bottom row, orange/green) saRNA. Each plot shows two overlaid populations distinguished by anti-FLAG staining (x-axis) and GFP expression (y-axis) analysed 24 hours post-saRNA transfection. **(C)** Western blot analysis of experiment described in (B). **(D)** Representative contour plots of THP-1 cells 24 hours post-transfection with CHIKV-GFP-B2^{WT} or -B2^{R59Q} saRNA and treated with PKR inhibitor C16 (1µM) or DMSO. **(E)** GFP^{high} MFI in THP-1-*Ifnar2*$^{-/-}$ cells transfected with CHIKV-GFP-B2^{WT} (red) or -B2^{R59Q} (orange) with increasing concentration of C16 or DMSO as vehicle control. Data are mean + s.d., n=4 independent experiments. (F) Fold increase of GFP^{+} THP-1-wt cells transfected with CHIKV-GFP-B2^{WT} (red) or -B2^{R59Q} (orange) with increasing concentrations of PKR inhibitor C16 relative to DMSO control. Fold increase was calculated relative to the average percentage GFP^{+} cells calculated for the DMSO control. Data are mean + s.d., n=4 independent experiments.

Statistical analysis was performed using two-way ANOVA followed by Tukey's multiple comparisons test. *$P < 0.05$; ns, not significant. arb. units, arbitrary units. Exact p-values and source data are provided as a Source Data file.

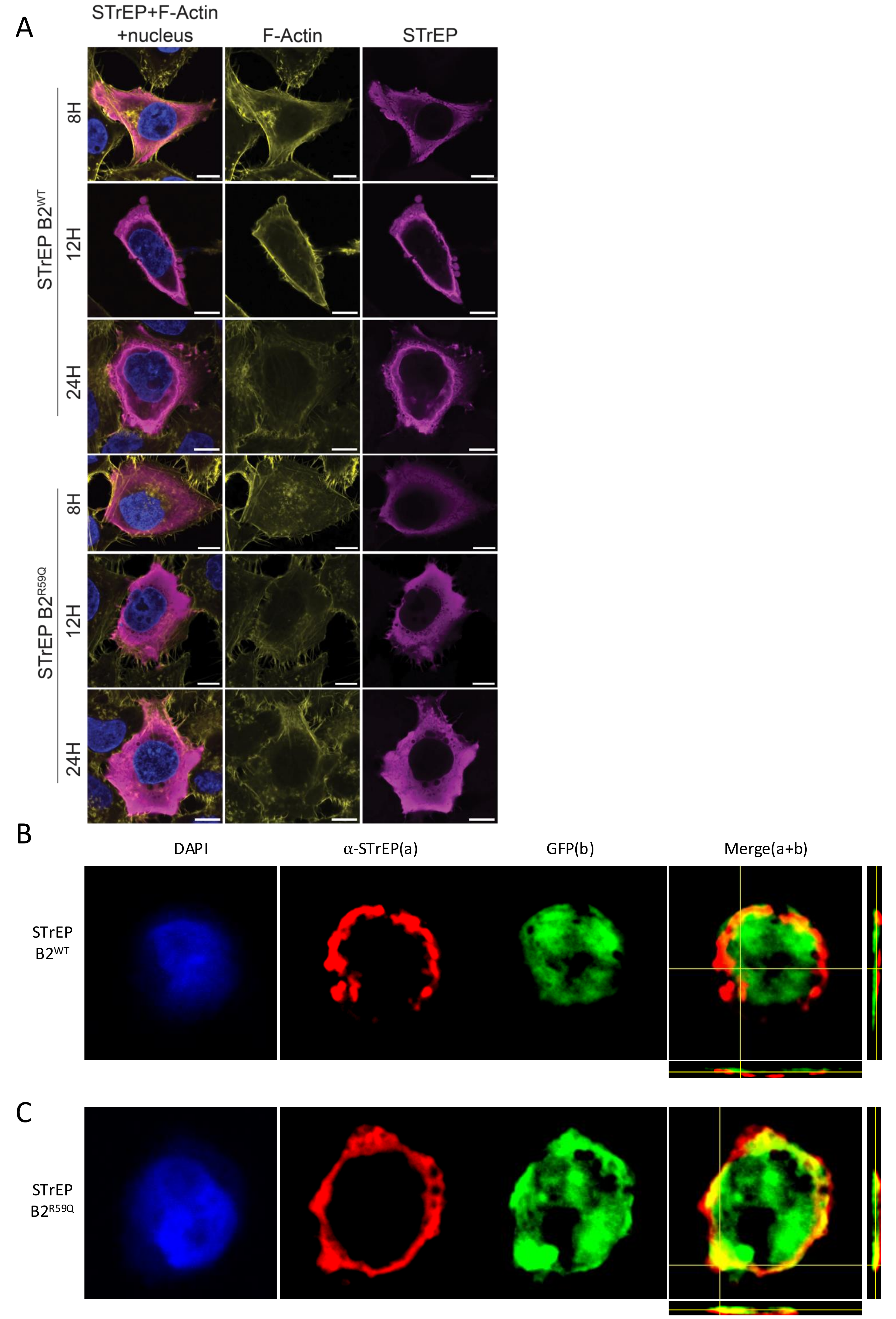
A
STrEP+F-Actin +nucleus
F-Actin
STrEP
STrEP B2WT
8H
12H
24H
STrEP B2R59Q
8H
12H
24H
B
DAPI
α-STrEP(a)
GFP(b)
Merge(a+b)
STrEP B2WT
C
STrEP B2R59Q

Figure S5

Figure S5. Subcellular localisation of NoV B2^{WT} and B2^{R59Q} by confocal microscopy in HeLa *PKR*$^{-/-}$ and THP-1 *Ifnar2*$^{-/-}$ cells.

(**A**) HeLa *PKR*$^{-/-}$ cells were transfected with CHIKV-GFP saRNA encoding STrEP-B2^{WT} or STrEP-B2^{R59Q} and analysed by confocal microscopy at 8, 12 and 24 hours post-transfection. Cells were stained for STrEP tag (magenta), nuclei (blue) and F-actin (yellow). **(B, C)** Confocal microscopy image of THP-1 *Ifnar2*$^{-/-}$ cells transfected with CHIKV-GFP saRNA encoding STrEP-B2^{WT} (B) or STrEP-B2^{R59Q} (C) stained for nuclei (blue) and STrEP-tag (red). GFP (green) is encoded by the saRNA. Images lining the merged image represent the orthogonal views.

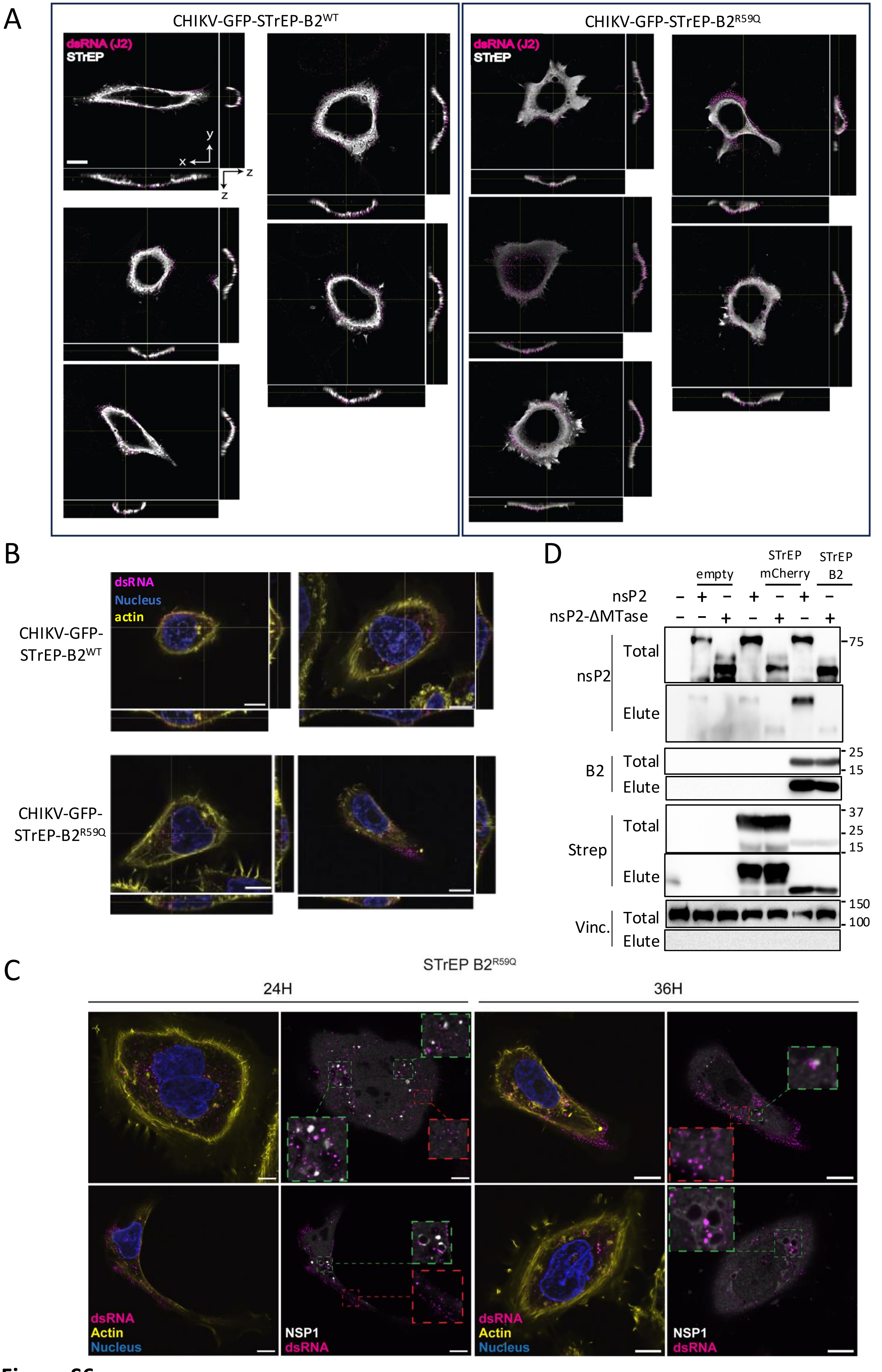
A
CHIKV-GFP-STrEP-B2WT
CHIKV-GFP-STrEP-B2R59Q
dsRNA (J2)
STrEP
B
dsRNA
Nucleus
actin
CHIKV-GFP-STrEP-B2WT
CHIKV-GFP-STrEP-B2R59Q
D
empty
STrEP mCherry
STrEP B2
nsP2
nsP2-ΔMTase
Total
Elute
B2
Strep
Vinc.
75
25
15
37
150
100
C
STrEP B2R59Q
24H
36H
dsRNA
Actin
Nucleus
NSP1

Figure S6

Figure S6. dsRNA distribution in saRNA-transfected cells expressing NoV B2^{WT} or B2^{R59Q} and interaction of NoV B2 with the nsP2 MTase-like domain.

(A) HeLa *PKR*$^{-/-}$ cells were transfected with CHIKV-GFP saRNA encoding STrEP-B2^{WT} (left) or CHIKV-GFP-STrEP-B2^{R59Q} (right) saRNA and analysed by confocal microscopy 20 hours post-transfection. Cells were stained for STrEP tag (white) and dsRNA (magenta). Scale bar is 10 µm. All images are lined with orthogonal views showing distribution of STrEP signal and dsRNA. **(B)** Representative images of HeLa *PKR*$^{-/-}$ cells transfected with CHIKV-GFP-STrEP-B2^{WT} or -STrEP-B2^{R59Q} for 36 hours. Cells were stained for dsRNA (magenta), nuclei (blue) and F-actin (yellow). Scale bar is 10 µm**.** All images are lined with orthogonal views showing distribution of STrEP signal, F-actin and dsRNA. **(C)** Representative images of HeLa *PKR*$^{-/-}$ cells transfected with CHIKV-GFP-STrEP-B2 ^{R59Q} for 24 hours or 36 hours. Cells were co-stained for nsP1 (white), dsRNA (magenta), F-actin (yellow) and nuclei (blue). Insets show magnified views with green dashed squares indicating dsRNA in nsP1-enriched cytoplasmic structures and red dashed squares indicating dsRNA in regions lacking nsP1. Scale bars: 10 µm. **(D)** HEK293T cells were transfected with plasmids expressing STrEP-tagged mCherry (STrEP-mCherry), STrEP-tagged NoV-B2^{WT} (STrEP B2) or empty plasmid (empty) together with constructs expressing either full-length nsP2 or nsP2 lacking the C-terminal MTase-like domain (nsP2-ΔMTase). Western blotting using the indicated antibodies was performed following STrEP-mediated immunoprecipitation. Source data are provided as a Source Data file.

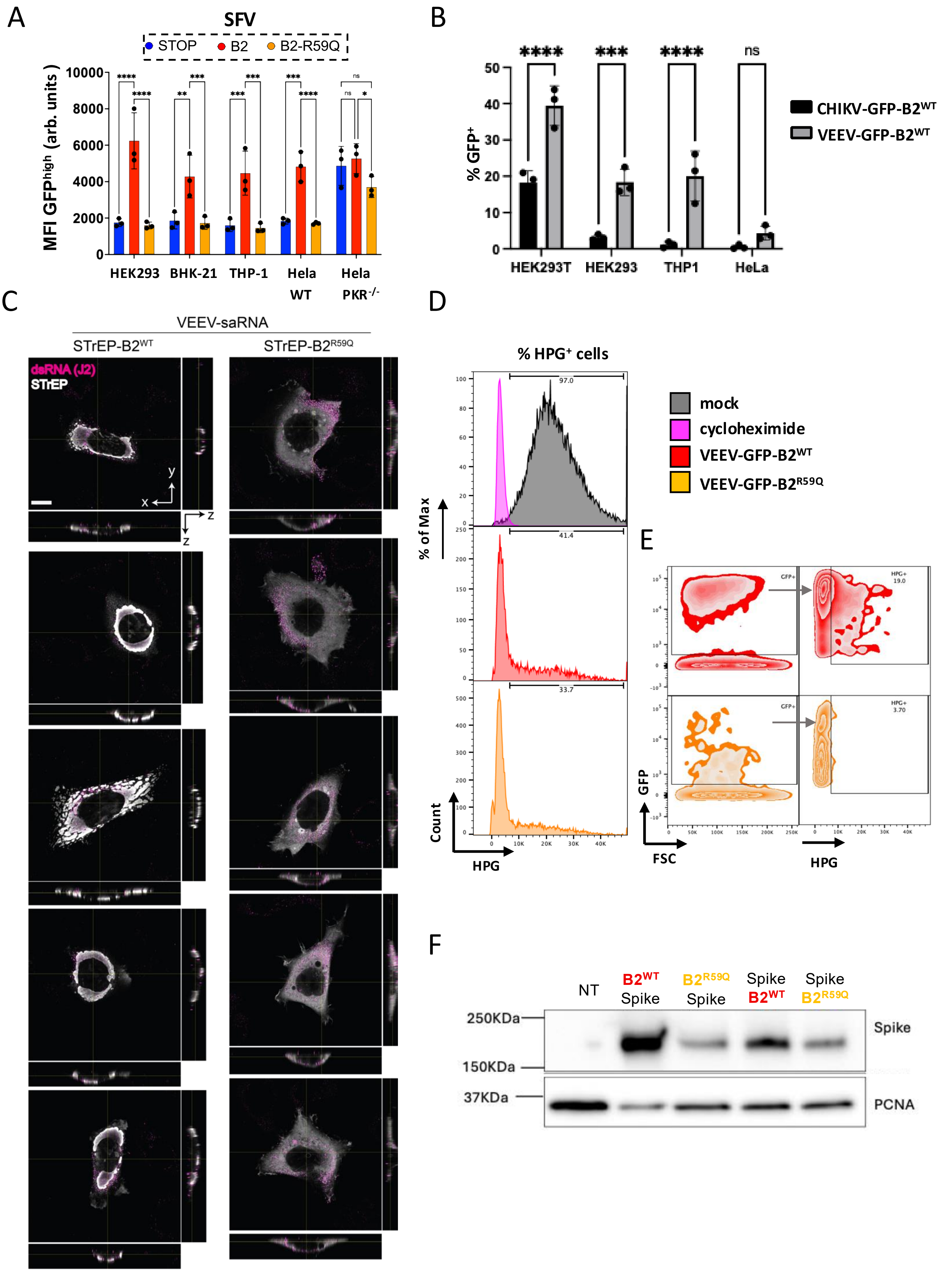
A
SFV
STOP
B2
B2-R59Q
MFI GFPhigh (arb. units)
HEK293
BHK-21
THP-1
Hela WT
Hela PKR-/-
B
% GFP+
CHIKV-GFP-B2WT
VEEV-GFP-B2WT
HEK293T
HEK293
THP1
HeLa
C
VEEV-saRNA
STrEP-B2WT
STrEP-B2R59Q
dsRNA (J2)
STrEP
D
% HPG+ cells
mock
cycloheximide
VEEV-GFP-B2WT
VEEV-GFP-B2R59Q
% of Max
Count
HPG
E
GFP
FSC
HPG
F
NT
B2WT Spike
B2R59Q Spike
Spike B2WT
Spike B2R59Q
250KDa
150KDa
37KDa
Spike
PCNA

Figure S7

Figure S7. NoV B2 enhances saRNA expression across SFV and VEEV vectors by counteracting PKR restriction and restores global translation.

(A) HEK293T, BHK-21, THP-1, and HeLa WT and Hela *PKR*$^{-/-}$ cells were transfected with SFV-GFP-STOP (blue), SFV-GFP-B2^{WT} (red) or SFV-GFP-B2^{R59Q} (orange) saRNA. Flow cytometry was used to monitor MFI of GFP^{high} population 24 hours post-transfection. **(B)** Percentage of GFP^{+} cells in HEK293T, HEK293, THP-1, and HeLa cells transfected with CHIKV-GFP-B2^{WT} and VEEV-GFP-B2^{WT} saRNAs. (A, B) Data are mean ± s.d., n=3 independent experiments where each point represents the average of technical replicates. **(C)** HeLa *PKR*$^{-/-}$ cells were transfected with VEEV-GFP saRNA encoding STrEP-B2^{WT} (left) or STrEP-B2^{R59Q} (right) saRNA. Representative images show NoV B2 (STrEP, white) and dsRNA (J2, magenta). Scale bar is 10 µm. **(D)** Representative histogram plots of HeLa cells 24 hours post-transfection with VEEV-GFP-B2^{WT} (red) or -B2^{R59Q} (orange) saRNA, followed by HPG incorporation assay in order to measure translation initiation. Samples were compared to mock or cycloheximide-treated cells. **(E)** Gating strategy used for assessment of translation initiation by HPG click chemistry. Single cells were analysed for GFP expression and HPG incorporation measured using the Alexa Fluor 594 channel. **(F)** HEK293T cells were transfected with CHIKV saRNAs expressing NoV-B2^{WT} (B2, red) or NoV-B2^{R59Q} (R59Q, orange) upstream or downstream of SARS-CoV-2 Spike prefusion protein. Protein lysates were prepared 24 hours post-transfection and analysed by western blotting using the indicated antibodies. PCNA served as a loading control.

Statistical analyses were performed using two-way ANOVA with Tukey's (A) or Šídák 's (B) multiple comparisons test. *P < 0.05, **P < 0.01, ***P < 0.001, ****P < 0.0001, ns, not significant. arb. units, arbitrary units. Exact p-values and source data are provided as a Source Data file.

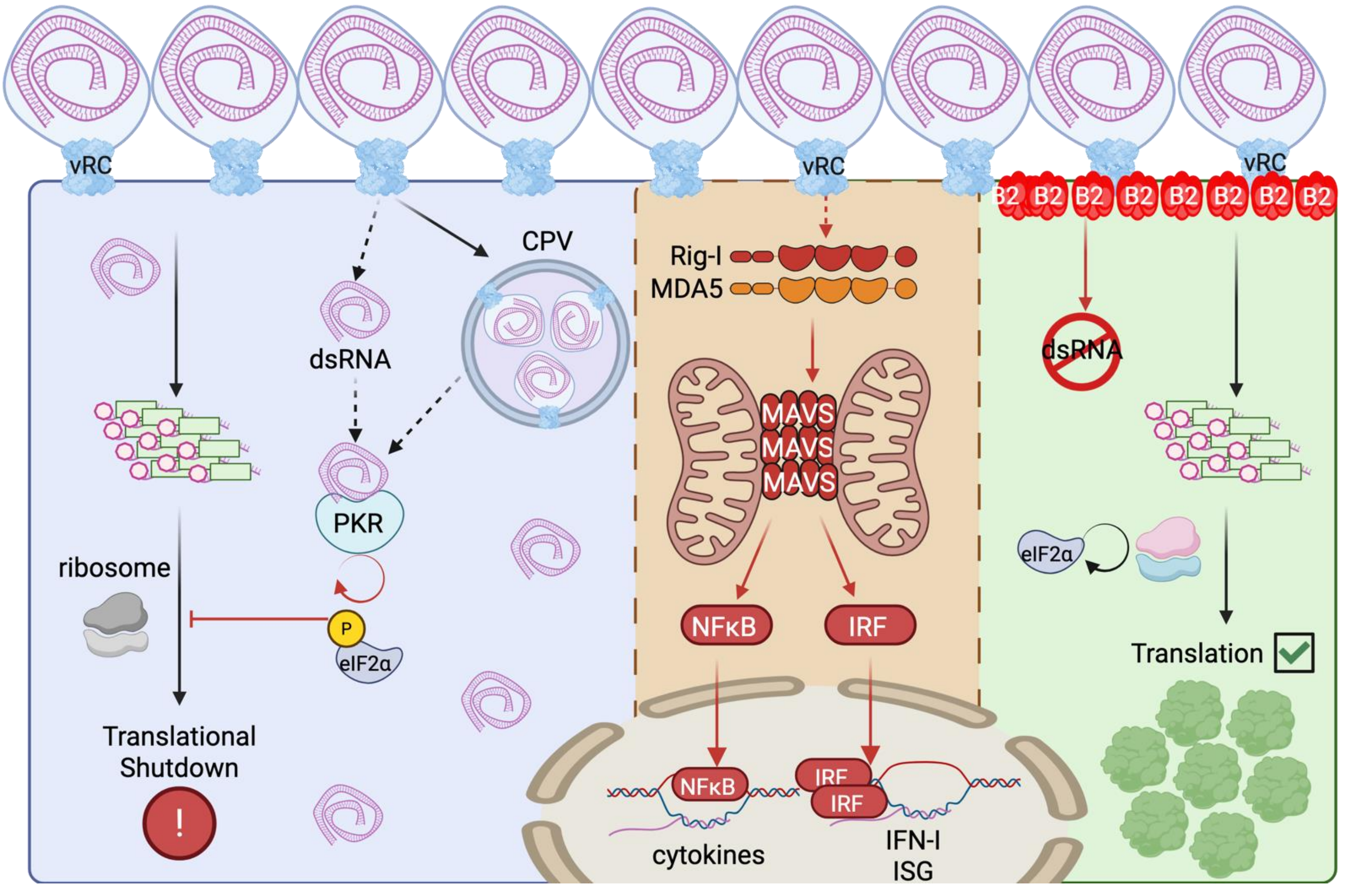

Figure S8. Proposed model of NoV B2 limiting dsRNA accessibility to PKR to rescue saRNA translation while preserving IFN-I responses.

saRNA amplification occurs within membrane-associated viral replication complexes (vRCs) at the plasma membrane and/or within type I cytopathic vesicles (CPVs). This process includes generation of double-stranded RNA (dsRNA) intermediates which can be released into the cytoplasm. Cytosolic dsRNA is sensed by PKR, leading to its activation and subsequent phosphorylation of eIF2α, which results in global translational shutdown. In parallel, RIG-I–like receptors RIG-I and MDA5 trigger MAVS-dependent signalling at mitochondria and downstream activation of NF-κB and IRF transcription factors, ultimately inducing pro-inflammatory cytokines and IFN-I responses. In the presence of NoV B2, dsRNA is sequestered and its release into the cytoplasm is prevented, thereby inhibiting PKR activation and avoiding eIF2α phosphorylation. This preserves cellular translation while maintaining equivalent activation of the RIG-I/MDA5–MAVS signalling axis and downstream IFN-I and ISG induction. "Figure S8." created in BioRender. Maillard, P. (https://BioRender.com/y7q3q1p) is licensed under CC BY 4.0.

Figure S8

Supplementary Table S1 (antibodies).

antibody	company	Cat#
**mouse-anti-Flag**	Sigma-Aldrich	F1804
**mouse-anti-STrEP**	GeneTex	GTX628899
**rabbit-anti-STrEP**	Novus	NBP2-41075
**RIG-I**	Cell Signaling	3747S
**MDA5**	Cell Signaling	5321S
**IFIT1**	Cell Signaling	14769S
**PKR**	Abcam	AB184257
**Phospho-PKR**	Abcam	AB32036
**eiF2α**	Thermo Scientific	AHO0802
**Phospho-eiF2α**	Abcam	AB32157
**vinculin**	Abcam	AB129002
**CHIKV-NSP1**	Novus	NBP313441
**CHIKV-NSP2**	Life Technologies	MA547042
**HA-PR8**	Thermo FIscher	PA5-34929
**Spike-SARS**	Thermo FIscher	PA1-41165
**mouse anti-dsRNA J2**	exalpha/nordic	10010200
**rabbit anti-dsRNA J2**	Absolute antibodies	Ab01299-23.0
**PCNA**	Cell Signaling	13110S
**anti-rabbit-HRP**	Cell Signaling	7074S
**anti-mouse-HRP**	Cell Signaling	7076S
**Goat anti-Rabbit IgG Alexa Fluor™ 405**	Invitrogen	A48258
**Goat anti-mouse IgG Alexa Fluor™ 405**	Invitrogen	A31553
**Goat anti-mouse IgG1 Alexa Fluor™ 568**	Invitrogen	A21124
**Goat Anti-mouse IgG, Alexa Fluor™ 647**	Invitrogen	A32728
**Goat Anti-rabbit IgG, Alexa Fluor™ 647**	Invitrogen	A32733

Supplementary Table S2 (primers).

Gene	purpose	Forward	Reverse
**CCL5**	qPCR	CCCAGCAGTGGTCTTTGTC	TCCCGGAACCCATTCTTCTCT
**CHIKV-NSP**	qPCR	ATTCTGCTTACACACAGACGTCTCATG	TTTGTCGAGTATGAGGGGTAGGCACC
**DDX58 (RIG-I)**	qPCR	GGACGTGGCAAAACAAATCAG	GCAATGTCAATGCCTTCATCA
**GFP**	qPCR	CTGCTGCCCGACAACCAC	ACCATGTGATCGCGCTTCTC
**IFIT1**	qPCR	CCTGAAAGGCCAGAATGAGG	TCCACCTTGTCAGGTAAGT
**IFIT2**	qPCR	ACTATGCCTGGGTCTACTATCA	ACTATGCCTGGGTCTACTATCA
**CHIKV (-) Strand**	Reverse Transcription	TTTTTGAAGGCCCTGCAACG	

Supplementary Tables
