# Supplementary material for: Tuning intracellular immunity by Nodamura virus B2 protein enhances self-amplifying RNA activity
Source: Nat Commun. 2026 Sep 28;17:10028. doi: 10.1038/s41467-026-77816-2 (PMC13620136; doi:10.1038/s41467-026-77816-2)
Supplement: Supplementary file 2 [file 41467_2026_77816_MOESM2_ESM.pdf]

nature portfolio

Corresponding author(s): Dr Pierre Maillard

Last updated by author(s): 290726

Reporting Summary

Nature Portfolio wishes to improve the reproducibility of the work that we publish. This form provides structure for consistency and transparency in reporting. For further information on Nature Portfolio policies, see our Editorial Policies and the Editorial Policy Checklist.

Statistics

For all statistical analyses, confirm that the following items are present in the figure legend, table legend, main text, or Methods section.

n/a	Confirmed	
☐	☒	The exact sample size (n) for each experimental group/condition, given as a discrete number and unit of measurement
☐	☒	A statement on whether measurements were taken from distinct samples or whether the same sample was measured repeatedly
☐	☒	The statistical test(s) used AND whether they are one- or two-sided<br>*Only common tests should be described solely by name; describe more complex techniques in the Methods section.*
☒	☐	A description of all covariates tested
☐	☒	A description of any assumptions or corrections, such as tests of normality and adjustment for multiple comparisons
☐	☒	A full description of the statistical parameters including central tendency (e.g. means) or other basic estimates (e.g. regression coefficient) AND variation (e.g. standard deviation) or associated estimates of uncertainty (e.g. confidence intervals)
☐	☒	For null hypothesis testing, the test statistic (e.g. F, t, r) with confidence intervals, effect sizes, degrees of freedom and P value noted<br>*Give P values as exact values whenever suitable.*
☒	☐	For Bayesian analysis, information on the choice of priors and Markov chain Monte Carlo settings
☒	☐	For hierarchical and complex designs, identification of the appropriate level for tests and full reporting of outcomes
☒	☐	Estimates of effect sizes (e.g. Cohen's d, Pearson's r), indicating how they were calculated

Our web collection on statistics for biologists contains articles on many of the points above.

Software and code

Policy information about availability of computer code

Data collection	FlowJo Software BD Biosciences 10.10.0<br>Roche LIGHTCYCLER Software Roche Unknown anaconda 3 5.2.0Sequencing was<br>small RNA sequencing was aperformed on an Illumina NextSeq sequencer at 1x75 bp read length
Data analysis	GraphPad Prism Software Graph Pad Software 10.4.0<br>ImageJ Software NIH 1.53<br>Airyscan processing' package in the Zen black (Zeiss) software<br>Papers (ReadCube) 5.4.14<br>Microsoft Office Microsoft 365<br>Microsoft Windows Microsoft 10.0.19045<br>Arivis (Zeiss)<br>UMI-tools<br>Burrows-Wheeler Aligner (BWA)<br>DESeq2<br>BioRender

For manuscripts utilizing custom algorithms or software that are central to the research but not yet described in published literature, software must be made available to editors and reviewers. We strongly encourage code deposition in a community repository (e.g. GitHub). See the Nature Portfolio guidelines for submitting code & software for further information.

Data

Policy information about availability of data

All manuscripts must include a data availability statement. This statement should provide the following information, where applicable:

- Accession codes, unique identifiers, or web links for publicly available datasets
- A description of any restrictions on data availability
- For clinical datasets or third party data, please ensure that the statement adheres to our policy

The small RNA deep sequencing data generated in this study have been deposited in NCBI's Gene Expression Omnibus (GEO) and are accessible through GEO Series accession GSE341822 (https://www.ncbi.nlm.nih.gov/geo/query/acc.cgi?acc=GSE341822).and Source data are provided with this study. All this information an be found within the data availability section.

Research involving human participants, their data, or biological material

Policy information about studies with human participants or human data. See also policy information about sex, gender (identity/presentation), and sexual orientation and race, ethnicity and racism.

Reporting on sex and gender	N/A
Reporting on race, ethnicity, or other socially relevant groupings	N/A
Population characteristics	N/A
Recruitment	N/A
Ethics oversight	N/A

Note that full information on the approval of the study protocol must also be provided in the manuscript.

Field-specific reporting

Please select the one below that is the best fit for your research. If you are not sure, read the appropriate sections before making your selection.

[x] Life sciences [ ] Behavioural & social sciences [ ] Ecological, evolutionary & environmental sciences

For a reference copy of the document with all sections, see nature.com/documents/nr-reporting-summary-flat.pdf

Life sciences study design

All studies must disclose on these points even when the disclosure is negative.

Sample size	Sample sizes were chosen to achieve a minimum of three independent biological replicates to ensure reproducibility and are stated in figure legends.
Data exclusions	Data were not excluded from the analyses.
Replication	All experiments were repeated at least three times to ensure reproducibility, with the exception of small RNA deep sequencing that was repeated twice.
Randomization	Randomisation was not necessary for the type of most experiments in this study. For microscopy, random cells were selected based on GFP positivity to then analysed the localisation of dsRNA, StreP-tagged B2, NsP1 and/or nsP2.
Blinding	Blinding was not necessary for most experiments in this study as most data are qualitative or semi-qualitative and measured by machines.

Reporting for specific materials, systems and methods

We require information from authors about some types of materials, experimental systems and methods used in many studies. Here, indicate whether each material, system or method listed is relevant to your study. If you are not sure if a list item applies to your research, read the appropriate section before selecting a response.

Materials & experimental systems

n/a	Involved in the study
☐	☒ Antibodies
☐	☒ Eukaryotic cell lines
☒	☐ Palaeontology and archaeology
☒	☐ Animals and other organisms
☒	☐ Clinical data
☒	☐ Dual use research of concern
☒	☐ Plants

Methods

n/a	Involved in the study
☒	☐ ChIP-seq
☐	☒ Flow cytometry
☒	☐ MRI-based neuroimaging

Antibodies

Antibodies used

mouse-anti-Flag Sigma-Aldrich F1804
mouse-anti-STrEP GeneTex GTX628899
rabbit-anti-STrEP Novus NBP2-41075
RIG-I Cell Signaling 3747S
MDA5 Cell Signaling 5321S
IFIT1 Cell Signaling 14769S
PKR Abcam AB184257
Phospho-PKR Abcam AB32036
eiF2 Thermo Scientific AHO0802
Phospho-eiF2 Abcam AB32157
vinculin Abcam AB129002
CHIKV-NSP1 Novus NBP313441
CHIKV-NSP2 Life Technologies MA547042
HA-PR8 Thermo FIscher PA5-34929
Spike-SARS Thermo FIscher PA1-41165
mouse anti-dsRNA J2 exalpha/nordic 10010200
rabbit anti-dsRNA J2 Absolute antibodies Ab01299-23.0
PCNA Cell Signaling 13110S
anti-rabbit-HRP Cell Signaling 7074S
anti-mouse-HRP Cell Signaling 7076S
Goat anti-Rabbit IgG Alexa Fluor™ 405 Invitrogen A48258
Goat anti-mouse IgG Alexa Fluor™ 405 Invitrogen A31553
Goat anti-mouse IgG1 Alexa Fluor™ 568 Invitrogen A21124
Goat Anti-mouse IgG, Alexa Fluor™ 647 Invitrogen A32728
Goat Anti-rabbit IgG, Alexa Fluor™ 647 Invitrogen A32733

Validation

All antibodies were validated by the manufacturer to react with their target protein. Primary antibodies used for western blots were further validated in-house by confirming a signal at the expected molecular weight. When possible, specificity was additionally confirmed by the absence of signal in extracts from knock out cell lines or from samples not expected to express the protein of interest (i.e. untreated, untransfected or untransduced controls). For immunofluorescence microscopy, primary antibodies were validated by confirming the absence of signal in cells known not to express the protein of interest and/or by using secondary antibody-only controls.

Eukaryotic cell lines

Policy information about cell lines and Sex and Gender in Research

Cell line source(s)

HEK293T, ATCC, ATCC CRL-3216
HEK293, ATCC, CRL-1573
HeLa PKR KO cells were a kind gift from Prof. Adam P. Geballe
HeLa WT, ATCC, CCL-2
THP-1-Dual™ , InvivoGen, thpd-nfis,
THP-1-Dual™ KO-IFNAR. InvivoGen, thpd-koifnar2
BHK-21, ATCC, ATCC CCL-10
E14 mESC, ATCC, CRL-1821

Authentication

Cell lines were commercially purchased and further validated by assessment of their morphology and/or functional characterisation.

Mycoplasma contamination

All cell lines were intially commercially purchased and their morphology, growth were regularly checked.

Commonly misidentified lines
(See ICLAC register)

N/A

Plants

Seed stocks	N/A
Novel plant genotypes	N/A
Authentication	N/A

Flow Cytometry

Plots

Confirm that:

- [x] The axis labels state the marker and fluorochrome used (e.g. CD4-FITC).
- [x] The axis scales are clearly visible. Include numbers along axes only for bottom left plot of group (a 'group' is an analysis of identical markers).
- [x] All plots are contour plots with outliers or pseudocolor plots.
- [x] A numerical value for number of cells or percentage (with statistics) is provided.

Methodology

Sample preparation	Cells were harvested by trypsinisation, washed with PBS containing 2% FCS and resuspended in PBS-4%-PFA (40-7401-05, Severn Biotech Ltd.), washed and analysed. For samples that required intracellular staining, cells were permeabilised (BD Perm/Wash™, 554723, BD Biosciences), blocked and stained with either a) anti-Flag, b) anti-STrEP, c) anti phospho-eIF2 antibodies for 2 hours at room temperature. Samples were then incubated with secondary antibody anti-mouse AF647 (A-21239, Invitrogen) for 2 hours at room temperature in the dark. Cells were washed 3 times with PBS and resuspended in 200 µL of FACS buffer (PBS, 2% FCS, 2 mM EDTA).
Instrument	BD LSR II Flow Cytometer
Software	Data analysis was performed with FlowJo (TreeStar).
Cell population abundance	10,000 cells with the gate of interest were acquired .
Gating strategy	Cells were first gated based on side scatter area (SSC-A) versus forward scatter area (FSC-A). Singlets were characterised using FSC-Height (FSC-H) versus FSC-A to exclude doublets. Single cells were then analysed for GFP expression. Cells with an MFI greater than 10^3 were classified as GFPhigh.

- [x] Tick this box to confirm that a figure exemplifying the gating strategy is provided in the Supplementary Information.
