# Supplementary material for: Tuning intracellular immunity by Nodamura virus B2 protein enhances self-amplifying RNA activity
Source: Nat Commun. 2026 Sep 28;17:10028. doi: 10.1038/s41467-026-77816-2 (PMC13620136; doi:10.1038/s41467-026-77816-2)
Supplement: Supplementary file 3 [file 41467_2026_77816_MOESM3_ESM.pdf]

nature portfolio

Peer Review File

Tuning intracellular immunity by Nodamura virus B2 protein enhances self-amplifying RNA activity.

Corresponding Author: Dr Pierre V Maillard

This file contains all reviewer reports in order by version, followed by all author rebuttals in order by version.

Version 0:

Reviewer comments:

Reviewer #1

(Remarks to the Author)
This study investigates how to improve vaccine development based on self-amplifying RNA (sa-RNA, also known as virus replicon), which offers many advantages over conventional mRNA vaccines. The authors show that simultaneous expression of a viral RNAi suppressor protein B2 from Nodamura virus significantly enhances foreign antigen protein expression from sa-RNA of two different alphaviruses (chikungunya and Venezuelan equine encephalitis viruses) in several cell cultures. They demonstrate an essential role for the dsRNA-binding activity of B2 in the suppression of antiviral immunity in both stem cells and mature somatic cells and suggest an involvement of B2 interaction with the viral replicase in the immune suppression. Interestingly, the authors found that whereas B2 inhibits the Dicer antiviral RNAi activity in stem cells, suppression of PKR-driven translational shutdown plays a key role in B2-dependent enhancement in somatic cells. These findings are novel and timely and will facilitate current vaccine development targeting various human diseases. I have only a few minor suggestions for the authors to incorporate into the revised manuscript.

1. Fig. 2J shows no significant differences in MFI GFPhigh (AU) from either Chikv-gfp-B2 or Chikv-gfp-B2-R59Q between WT and RIG-I-/-MDA5-/- cells. Please perform similar comparisons to show whether Chikv-gfp-stop, Chikv-gfp-B2 or Chikv-gfp-B2-R59Q directs GFP expression at significantly different levels between HEK293T and HEK293T-PKR-/- cells (lines 439-447/Fig. 4A). Similarly, does Chikv-gfp-stop, Chikv-gfp-B2 or Chikv-gfp-B2-R59Q direct GFP expression at significantly different levels between HeLa and HeLa-PKR-/- cells (lines 448-449/Fig. 4B)?
2. It is curious why RIG-I/MDA5 double knockout does not enhance gene expression from the CHIKV replicon (Fig. 2J). In contrast, ref 41 described drastic differences in GFP expression from CHIKV replicons between WT and RIG-I/MDA5 double knockout NIH 3T3 cells. Ref 41 compared GFP expression after the infection of cells with packaged viral particles that contain the replicon RNA. Moreover, a recent study indicates that the productive infection of cultured cells with CHIKV resulted in very low levels of eIF2α phosphorylation as compared to alphaviruses SINV and SFV (Frolova EI et al., J Virol 2023, 97, e0097923-23). Thus, I am wondering whether unlike natural viral infection, direct replicon RNA transfection via Lipofectamine in this work triggers a different innate immune response. This issue can be readily resolved by comparing GFP expression from wildtype and PKR knockout cells infected with the described CHIKV replicons packaged in viral particles.

3. Further proof-reading is necessary. See below for a few examples:
Line 244: change “50’000” to “50,000”;
Line 507: change “form” to “forms”;
Line 546: insert “and” before dsRNA;

Reviewer #2

(Remarks to the Author)
Summary
In ‘Tuning intracellular immunity by Nodamura virus B2 protein enhances self-amplifying RNA activity’, describe R.Y. Sanchez-David et al. a novel self-amplifying RNA design to boost gene of interest expression. This is a highly relevant development as improved gene of interest expression could lower the initial vaccine dose, reduce costs, and increase

vaccine efficiency.

By adding the Nodamura virus B2 gene to two alphavirus-based self-amplifying RNA vectors, the gene of interest expression was increased in a stem cell line and a couple of somatic cell lines. The authors also unraveled insights into the molecular mechanism by showing the involvement of a Dicer-mediated RNA interference and the protein kinase R-driven translational shutdown. Furthermore, confocal images show that the addition of B2 results in retention of the dsRNA in replication spherules at the plasma membrane and binding of B2 to the alphavirus non-structural protein 2. The exact mechanism behind this observation is not discussed in this manuscript.

Overall, this is a valuable study with some limitations in the transfection efficiency of the self-amplifying RNA. Although B2 boosts protein production for the chikungunya virus-based vector, this vector seems less relevant by its low transfection efficiency; only a limited number of cells express the GFP marker. This in contrast to the widely used Venezuelan equine encephalitis virus-based vector which shows a higher percentage of GFP expressing cells. This vector is known for its high gene of interest expression levels, but the addition of the B2 gene shows to increase this expression level further. In addition to a GFP marker, the self-amplifying RNA-induced expression of two antigens is shown as well. I am looking forward to upcoming research in which the efficiency of this novel self-amplifying RNA vector is evaluated in vivo.

Major comments

- The authors use a very systematic transfection approach (RNA quantification and integrity check) to make the comparison between the different sa-RNA vectors as reliable as possible. Nevertheless, RNA is delegate and transfection efficiency can vary greatly. In this manuscript, the transfection efficiency also seems to vary between different repeats based on the variation in the percentage GFP positive cells after CHIKV-GFP-B2 transfection in mESCs in Figure S1B and Figure 1E, respectively 15% and 6%. Furthermore, the percentage of GFP positive cells is low in many transfections, e.g. below 4% in HeLa cells. I therefore wonder how reliable the results in Figure 1 and 2 are?
o In Figure 1, I miss a description of the amount of (independent) repetitions that have been performed for Figure 1B, C, E and F. Are the results consistent over multiple independent repetitions?
o In Figure 2, comparisons are made in HeLa and HEK293 cells, which both show low percentages of GFP positive cells after transfection (based on Figure 2G and Figure S6). I suggest that the authors add another cell line in which a higher percentage of GFP positive cells is reached, or use another transfection method (e.g. electroporation) to increase the percentage of GFP positive cells.

- Line 402: the authors state that GFP expression is absent for CHIKV-GFP-B2R59Q, but in Figure 2F the MFI GFP high levels are not zero and also in Figure 2G a low level of GFP positive cells is described. If these levels are comparable to background levels, could the authors please visualize that in the graphs of Figure 2F and 2G.

- Line 403-406: the authors conclude that the higher levels of GFP and nsP1 (+)-sense RNA, observed after CHIKV-GFP-B2WT transfection, are due to the B2 dsRNA-binding ability. However, the percentage of GFP positive cells is also higher for the CHIKV-GFP-B2WT vector compared to the other two vectors. Could this be another explanation for the increased RNA levels, or was the RNA extracted from only GFP positive cells?

- Line 446-448: Figure 4A shows that the GFP expression levels in HEK293T Dicer-/- PKR-/- were similar for all three constructs. The CHIKV-GFP-STOP and CHIKV-GFP-B2R59Q are higher, while the CHIKV-GFP-B2WT levels are lower than in the other three described cell lines. Is there an explanation why the CHIKV-GFP-B2WT GFP expression drops in this cell line?

- Line 479-481: the authors describe that the P-PKR levels in the THP1 transfected cells are similar for CHIKV-GFP-B2R59Q and CHIKV-GFP-B2WT, but that is not very clear from the western blot (vague bands and shadows). Can the authors provide a clearer western blot for the PKR and P-PKR detection? Perhaps the antibody is just too bad to improve the western blot and the authors can show in another way that the PKR and P-PKR levels are similar.

- Line 481-484: based on Figure 4H, I would not state that P-eIF2α is absent in CHIKV-GFP-B2WT transfected cells, and present in CHIKV-GFP-B2R59Q transfected cells. Both panels show equally vague levels of P-eIF2α, whereas the results from Figure 4F and 4G are more convincing.

- Line 515-516: visually there seems a difference between the B2 distribution of the CHIKV-GFP-B2R59Q and CHIKV-GFP-B2WT in Figure 5 and Figure 5S, however cell markers are lacking to conclude that NoV B2WT localizes to the periphery and B2R59Q throughout the cytoplasm. I suggest that the authors add a panel of stains to show co-localization with the periphery and the cytoplasm, or at least of the nucleus as in Figure 6C and D.

- Line 518: I assume that the yellow color in Figure S5A indicates overlap between B2 and GFP in the cytoplasm. Based on this, I think it is incorrect to state in line 518 that there is 'no overlap with GFP'.

- Alphaviruses are known to internalize their dsRNA-containing spherules in type I cytopathic vacuoles (CPV-I) (Girard et al. Journal of Virology 2024). It is therefore very interesting that the dsRNA does not internalize in the presence of B2, and keeps located at the cell periphery (Figure 5 and 6). Based on this, I have some questions and comments:
o At which time point post transfection were the cells in Figure 5A-D and Figure 6C-D stained, and is there a difference with later time points, e.g. does relocation of the dsRNA take place?
o Is the cytoplasmic dsRNA upon CHIKV-GFP-STrEP-B2R59Q transfection a result of the spherule internalization or do the authors believe that this is free floating dsRNA (as visualized in the graphical abstract)? A staining of dsRNA and nsP1

could create some more clarity.
o Does this lack of internalization alleviate PKR restriction, or creates B2 a sort of protective layer? Do the authors have a theory for this, and can they provide experimental evidence?
o Line 137-138 ‘B2 expression causes sa-RNA-derived dsRNA to localize to the cell periphery’ and line 549 ‘B2 can alleviate this restriction by spatially redistributing the dsRNA’, suggest that it is the B2 protein that causes the localization of the dsRNA at the plasma membrane, whereas I think it would be more correct to say that B2 prevents the re-localization of the dsRNA to the cytoplasm.

- Line 578: it was shown before that transfection of VEEV and CHIKV sa-RNA vectors in HeLa cells results in a low percentage of cells expressing the gene of interest. Did the authors compensate for potential differences in transfection efficiency that could result in higher overall Spike production? And is the same trend observed in other cell lines with higher percentage of gene of interest expressing cells?

- Line 683: the potential reduced cytotoxicity could further boost gene of interest expression as the cell might be able to longer produce the protein. This has an advantage, but could this also cause problems in overproduction of the gene of interest or potential overstimulation of the immune system?

Minor comments
-Line 134: suggestion to clarify in the sentence that this refers to sa-RNA vectors by changing the sentence in ‘CHIKV and VEEV-based sa-RNA vectors’.
-Line 142 and 621: suggestion to change ‘replicon vector’ in ‘sa-RNA vector’ to keep the text consistent and clear.
-Line 247 and 248: replace comma by full stop after ‘duplicates’ and ‘Switzerland’.
-Line 358: the authors use mainly ‘sa-RNA’ but a couple of times ‘saRNA’. For consistency, please keep to one abbreviation.
-Figure S1: In panel B the names of the vectors are different from the names in Figure 1 and the figure description. It would be recommended to adapt the name in Figure S1B. Furthermore, I was wondering what is meant with the vehicle in panel B. Can the authors explain this in the figure description.
-Figure 1: In panel A the schematic representation of the CHIKV sa-RNA vectors is too small to fully understand the design of the vectors. In panel B the x-axis description is missing.
-Line 737: I think that ‘HeLa cells’ should be ‘mESCs’?
-Line 745: the figure description states ’21- and 23-nt reads’, but in the text it states (line 381) ’21-23-nt viral reads’. I assume that the figure indeed shows the 21- to 23-nt reads? If so, please adapt this in the figure description.
-Line 505: in this case sa-RNA-GFP means CHIKV-GFP sa-RNA?
-Figure 5: there is some inconsistency in the names in the figure, e.g. ‘STrEP B2 – wt’ in A vs ‘STrEP B2WT’in D, and ‘αNSP2’ in E vs ‘αNsp2’ in F (in text described as nsP2).
-Line 854: M should be N. Description of M is missing.

Reviewer #3

(Remarks to the Author)
This manuscript investigates whether expression of the Nodamura virus (NoV) B2 protein can improve self-amplifying RNA (sa-RNA) expression by counteracting host antiviral defenses. The authors conclude that B2 suppresses RNAi in embryonic stem cells and alleviates PKR-mediated translational shutdown in somatic cells, thereby enhancing reporter or antigen expression from alphavirus replicons while maintaining interferon induction. They further suggest that this strategy could inform vaccine development.
While the study is technically competent, several issues limit its conceptual impact and overall significance.
Major Concerns
1. Lack of novelty and overstated claims
The central observation—that a dsRNA-binding viral suppressor of RNA silencing can improve replicon expression by blunting host defenses—is predictable. Viral proteins such as vaccinia E3L and influenza NS1 have long been known to block PKR and other sensors through dsRNA sequestration. That NoV B2 would exert a similar effect on synthetic alphavirus replicons is not a surprising advance. The claims of conceptual novelty and translational relevance are overstated.
2. Unclear biological relevance
The physiological role of B2 in mammalian immunity is not convincingly established. Nodamura virus has at best a limited ability to replicate in mammals, raising doubts that its interactions with PKR or RNAi pathways reflect an evolved immune antagonism rather than experimental artifact. Without stronger evidence that NoV naturally manipulates mammalian antiviral defenses, the significance of B2 as a “tool” for vaccine design remains questionable.
3. Insufficient mechanistic insight
The manuscript does not provide rigorous mechanistic explanations for many of the observed effects. The RNAi-related experiments in embryonic stem cells are inconclusive and do not demonstrate a functional antiviral role for Dicer. In somatic cells, the data argue against antiviral RNAi altogether. The dsRNA strand analysis is interpreted inconsistently—if B2 protected replication intermediates by binding dsRNA, both positive and negative strands should be stabilized, not just the positive strand. More direct assays (e.g., RNA immunoprecipitation) would be required to demonstrate B2 binding to replicon dsRNA.
Similarly, the claim that B2 protects replicons by sequestering dsRNA at the cell periphery or by interacting with alphavirus nsP2 is speculative. The interaction with nsP2 is not mechanistically developed, and it remains unclear whether it has any consequence for replicon biology. Overall, the evidence for how B2 alleviates PKR-mediated silencing is correlative rather

than definitive.
4. Overstated translational impact
The suggestion that co-opting B2 provides a viable strategy for sa-RNA vaccine improvement is premature. Introducing a viral immune evasion protein into vaccine platforms raises safety and immunogenicity concerns that are not addressed. The limited interferon data do not exclude broader effects on innate signaling, and the potential for dampening protective immunity remains unresolved.
5. Presentation and rationale
Experimental rationale is often missing. The choice of cell lines is not explained, and some assays (e.g., co-immunoprecipitations, eIF2α phosphorylation) are performed only in specific contexts without justification. Figures are numerous and descriptive, but not all are necessary; the Introduction and Discussion reiterate background material rather than providing synthesis or perspective.

In conclusion, the study shows that NoV B2 can enhance expression from alphavirus sa-RNAs by interfering with host defenses, but this is an expected outcome given prior knowledge of viral dsRNA-binding proteins. The mechanistic evidence is incomplete, the biological relevance in mammalian systems is uncertain, and the translational claims are overstated. While the experiments are competently executed, the work does not provide the level of conceptual advance or physiological insight required for publication in a high-impact journal.

Reviewer #4

(Remarks to the Author)
I co-reviewed this manuscript with one of the reviewers who provided the listed reports. This is part of the Nature Communications initiative to facilitate training in peer review and to provide appropriate recognition for Early Career Researchers who co-review manuscripts.

Version 1:

Reviewer comments:

Reviewer #1

(Remarks to the Author)
The authors have satisfactorily addressed all my questions.

Reviewer #2

(Remarks to the Author)
Thank you for your thorough and careful response to the review comments. The authors have addressed all my concerns in a convincing and systemic manner.

I appreciate the inclusion of an additional cell line in the transfection experiment, as well as the optimization of the transfection conditions, both enhanced the reliability of the results. Furthermore, the additional experiment in which RNA measurement was performed on GFP-sorted cells strengthens the evidence for the effect of B2 on the saRNA replication. The revised immunofluorescence analyses are clear and the additional immunofluorescence data provide a clearer understanding of the saRNA distribution pattern. I also appreciate the author’s insightful discussion on the potential mechanism of B2 localization, dsRNA distribution and PKR restriction.

Overall, the additional results and refined figures strengthen the manuscript and increase confidence in the conclusions. I have no further comments and recommend this manuscript for acceptance.

Reviewer #3

(Remarks to the Author)
The revised manuscript is substantially improved and the authors have addressed many of the major concerns raised in the initial review. In particular, they have added important new controls and experiments showing that the B2-dependent enhancement of saRNA expression is reproducible under high-transfection conditions in BHK-21 cells, is observed after VRP-mediated delivery, and extends to additional alphavirus backbones. They also now provide sorted-cell RT-qPCR data to address whether increased RNA levels simply reflect differences in transfection efficiency, improved PKR/eIF2α analyses, and new biochemical evidence that STrEP-tagged B2WT, but not B2R59Q, co-precipitates dsRNA in saRNA-transfected cells. These additions substantially strengthen the central claim that NoV B2 enhances saRNA-driven gene expression by counteracting PKR-dependent translational restriction in somatic cells while preserving measurable IFN/ISG induction.
The authors have also improved the rationale for several experimental choices, including the use of THP-1 reporter cells, HEK293T knockout lines, HeLa PKR–/– cells, and BHK-21 cells. The revised microscopy and nsP2 co-immunoprecipitation data provide a more coherent model in which B2 binds dsRNA and associates with the alphavirus replication environment, potentially limiting cytoplasmic redistribution of dsRNA and reducing access to PKR-dependent translational control. This model is plausible and interesting, although still not fully proven.
Some concerns remain. First, the evidence for a functional antiviral RNAi restriction of saRNA in mESCs remains

suggestive rather than definitive. The accumulation of 21–23 nt viral small RNAs and their reduction by B2WT are consistent with Dicer-mediated processing, but the manuscript still lacks direct genetic evidence that Dicer/Ago activity functionally suppresses saRNA expression in this system. The authors should therefore temper statements implying that antiviral RNAi is conclusively responsible for saRNA restriction in stem cells.
Second, although the new dsRNA co-IP/dot blot experiment addresses an important previous gap, it does not identify the dsRNA species bound by B2 or prove that this interaction is the causal event preventing PKR-mediated eIF2α phosphorylation. The authors should present this as supportive evidence rather than as a complete mechanistic explanation. Similarly, while the CHIKV nsP2 interaction is intriguing, the corresponding interaction with VEEV nsP2 was not tested, even though the mechanism is generalized to VEEV-based saRNA.
Finally, the translational claims should remain cautious. The data support a potentially useful engineering principle for increasing saRNA expression, but all experiments remain cell-culture based. In vivo immunogenicity, safety, toxicity, antigen overexpression, effects on broader innate signaling, and possible immune responses against B2 remain unresolved. The revised Discussion acknowledges some of these issues, but the vaccine-development framing should be further softened.
Overall, the revised manuscript is much stronger and addresses most of the prior concerns. I would support publication after minor revisions focused on tempering the RNAi and translational claims and clarifying the remaining mechanistic uncertainties.

Open Access This Peer Review File is licensed under a Creative Commons Attribution 4.0 International License, which permits use, sharing, adaptation, distribution and reproduction in any medium or format, as long as you give appropriate credit to the original author(s) and the source, provide a link to the Creative Commons license, and indicate if changes were made.
In cases where reviewers are anonymous, credit should be given to 'Anonymous Referee' and the source.
The images or other third party material in this Peer Review File are included in the article's Creative Commons license, unless indicated otherwise in a credit line to the material. If material is not included in the article's Creative Commons license and your intended use is not permitted by statutory regulation or exceeds the permitted use, you will need to obtain permission directly from the copyright holder.
To view a copy of this license, visit https://creativecommons.org/licenses/by/4.0/

We thank the four reviewers for their thorough evaluation of our work and for their helpful and constructive comments (below in italics). We have carefully addressed all concerns raised in this revised version as detailed in the point-by-point response below. In addition, we have revised the text and figures and performed additional experiments where appropriate in response to the reviewers' suggestions. We believe that these changes have improved the clarity and strength of the manuscript.

<u>Reviewer #1 (Remarks to the Author)</u>

This study investigates how to improve vaccine development based on self-amplifying RNA (sa-RNA, also known as virus replicon), which offers many advantages over conventional mRNA vaccines. The authors show that simultaneous expression of a viral RNAi suppressor protein B2 from Nodamura virus significantly enhances foreign antigen protein expression from sa-RNA of two different alphaviruses (chikungunya and Venezuelan equine encephalitis viruses) in several cell cultures. They demonstrate an essential role for the dsRNA-binding activity of B2 in the suppression of antiviral immunity in both stem cells and mature somatic cells and suggest an involvement of B2 interaction with the viral replicase in the immune suppression. Interestingly, the authors found that whereas B2 inhibits the Dicer antiviral RNAi activity in stem cells, suppression of PKR-driven translational shutdown plays a key role in B2-dependent enhancement in somatic cells. These findings are novel and timely and will facilitate current vaccine development targeting various human diseases. I have only a few minor suggestions for the authors to incorporate into the revised manuscript.

1. *Fig. 2J shows no significant differences in MFI GFPhigh (AU) from either Chikv-gfp-B2 or Chikv-gfp-B2-R59Q between WT and RIG-I-/-MDA5-/- cells. Please perform similar comparisons to show whether Chikv-gfp-stop, Chikv-gfp-B2 or Chikv-gfp-B2-R59Q directs GFP expression at significantly different levels between HEK293T and HEK293T-PKR-/- cells (lines 439-447/Fig. 4A).*

We thank the reviewer for requesting additional statistical comparisons between HEK293T and HEK293T *PKR*$^{-/-}$ *Dicer*$^{-/-}$ transfected with CHIKV-GFP-stop, CHIKV-GFP-B2^{WT} or CHIKV-GFP-B2-^{R59Q}. We have now included the requested statistical analyses in Figure 4A. For clarity, we also show below (for this rebuttal only) a direct side-by-side comparisons between 293T WT and 293T *PKR*$^{-/-}$ *Dicer*$^{-/-}$ transfected with the three saRNAs.

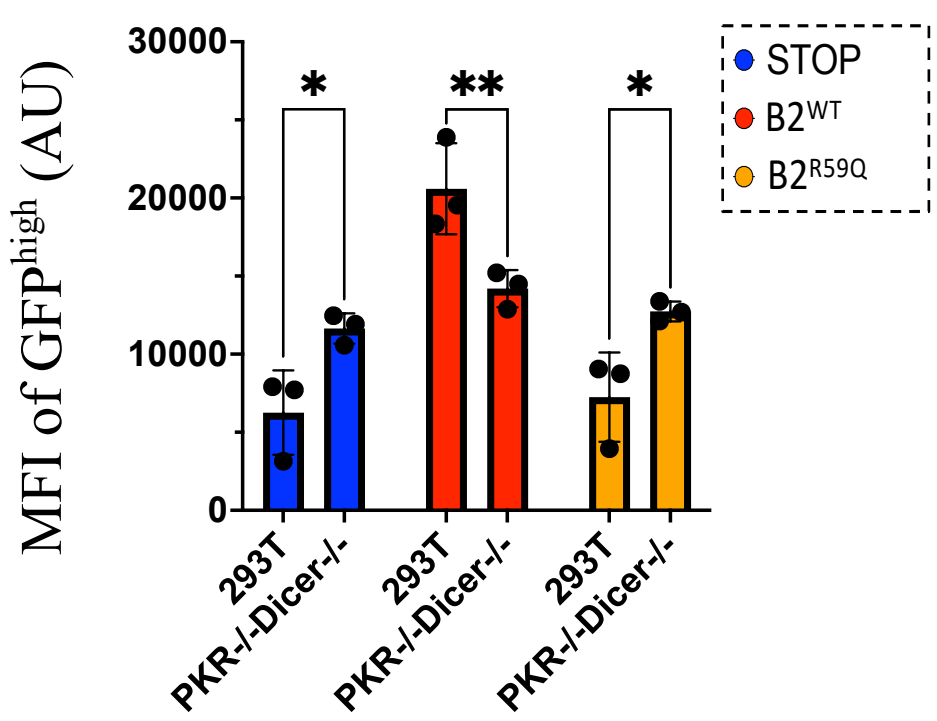

HEK293T WT and HEK293T Dicer$^{-/-}$ PKR$^{-/-}$ cells (derived from the parental Dicer$^{-/-}$ clone 4-25) were transfected with CHIKV-GFP-STOP (Blue), -B2^{WT} (red) or -B2^{R59Q} (orange) saRNA. Flow cytometry was used to monitor MFI of GFP^{high} population 24 hours after transfection.

We observed that GFP Mean Fluorescence Intensity (MFI) from CHIKV-GFP-STOP and CHIKV-GFP-B2^{R59Q} was higher in *PKR*$^{-/-}$ *Dicer*$^{-/-}$ compared to WT cells, consistent with PKR-mediated

restriction of these saRNAs. In contrast, expression of NoV B2 restored GFP expression in wild-type (WT) cells demonstrating the ability of NoV B2 to alleviate PKR-mediated restriction.

Additionally, we observed a reduction in GFP MFI in HEK293T $PKR^{-/-}$ $Dicer^{-/-}$ cells transfected with CHIKV-GFP-$B2^{WT}$ compared with WT cells. This effect was not observed in $RIG\text{-}I^{-/-}MDA5^{-/-}$ cells relative to WT cells transfected with CHIKV-GFP-$B2^{WT}$ (Figure 2J), nor in HeLa $PKR^{-/-}$ versus HeLa WT cells (see response to comment 2 and Fig. 4B). Given the discrepancy, we considered the possibility of a clone-specific effect. The HEK293T $PKR^{-/-}$ $Dicer^{-/-}$ cell line was a kindly provided by Prof. Bryan Cullen and was originally generated by knocking out PKR in a pre-existing $Dicer^{-/-}$ clone (clone 4-25)[1]. To address this possibility, we transfected the parental $Dicer^{-/-}$ clone 4-25 with the three saRNAs and found that GFP levels were comparable between $Dicer^{-/-}$ and $Dicer^{-/-}$ $PKR^{-/-}$ cells transfected with CHIKV-GFP-$B2^{WT}$. These results suggest that the reduced GFP levels observed in the double knockout line of HEK293T likely reflect a clone specific effect. We have incorporated the new data with clone 4-25 into Figure 4A, added the corresponding statistical analyses and revised the main text (lines 488-499) and the Figure 4A legend accordingly.

Figure 4A:

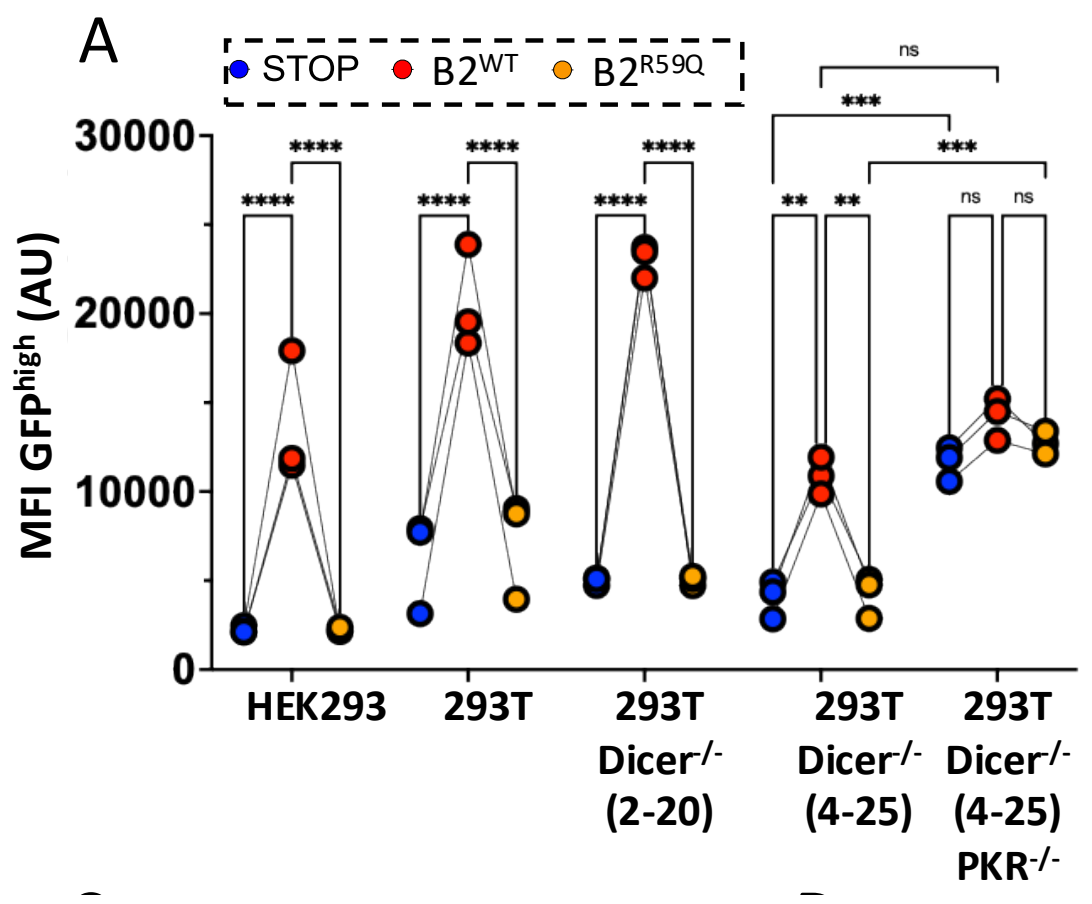

HEK293 and HEK293T as well as HEK293T $Dicer^{-/-}$ (clone 2-20 and clone 4-25) and HEK293T $Dicer^{-/-}$ $PKR^{-/-}$ cells (derived from the parental $Dicer^{-/-}$ clone 4-25[1,31]) were transfected with CHIKV-GFP-STOP (Blue), -$B2^{WT}$ (red) or -$B2^{R59Q}$ (orange). Flow cytometry was used to monitor MFI of GFP^{high} population at 24 hours after transfection. Data represent the mean ± s.d. of three independent experiments. Statistical analyses were performed using two-way ANOVA with Sidak's multiple comparisons test as post-test for pairwise comparisons. ***$P < 0.001$; ns, not significant.

2. *Similarly, does Chikv-gfp-stop, Chikv-gfp-B2 or Chikv-gfp-B2-R59Q direct GFP expression at significantly different levels between HeLa and HeLa-PKR-/- cells (lines 448-449/Fig. 4B)?*

We thank the reviewer for this comment. HeLa WT and HeLa $PKR^{-/-}$ cells were transfected with CHIKV-GFP-STOP, CHIKV-GFP-$B2^{WT}$ or CHIKV-GFP-$B2^{R59Q}$ and GFP expression was assessed by flow cytometry 24 hours post-transfection. We found that saRNAs encoding NoV $B2^{WT}$ resulted in enhanced GFP expression compared to saRNAs lacking B2 or encoding the $B2^{R59Q}$ mutant (see below revised Figure 4B). This enhancement was abolished in HeLa $PKR^{-/-}$ cells confirming that $B2^{WT}$ enhances saRNA-mediated gene expression by alleviating PKR-mediated restriction. Data from three independent experiments have now been incorporated in the revised Figure 4B, and the main text (lines 499-504) as well as the Figure 4B legend have been updated accordingly.

Figure 4B:

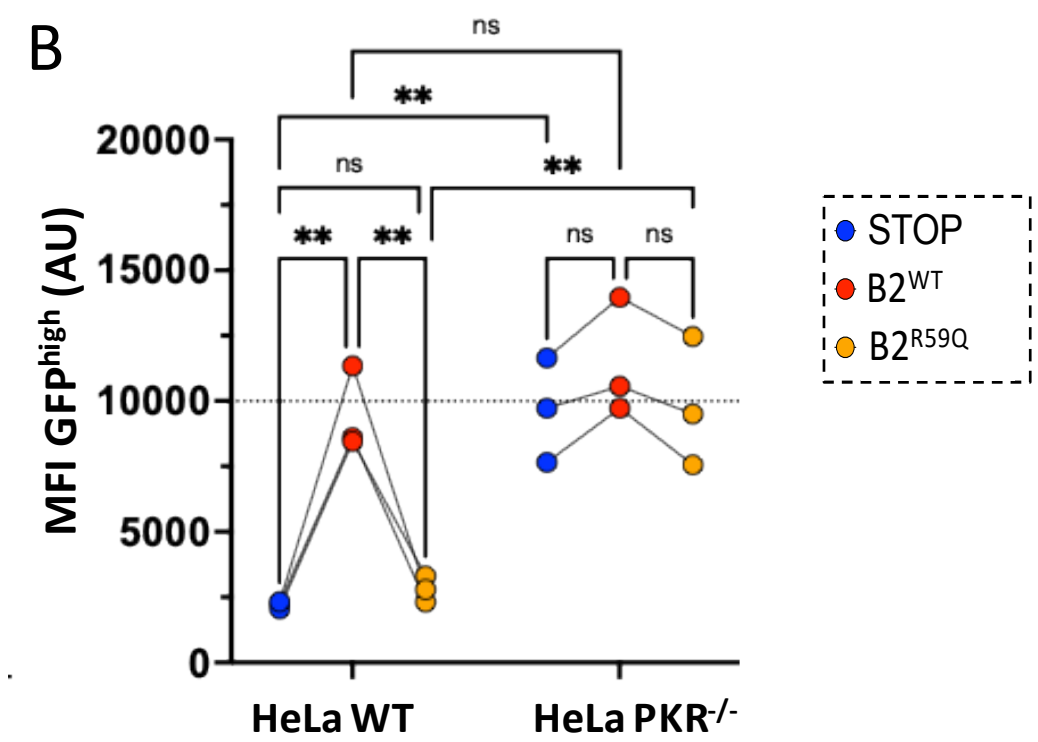

HeLa WT and HeLa $PKR^{-/-}$ cells were transfected with CHIKV-GFP-STOP (Blue), -$B2^{WT}$ (red) or -$B2^{R59Q}$ (orange). Of note, 1µg of saRNA was transfected in these experiments compared to 0.1µg used throughout the study. Data represent the mean ± s.d. of three independent experiments. Statistical analyses were performed using two-way ANOVA with Sidak's multiple comparisons test as post-test for pairwise comparisons. **P < 0.01; ns, not significant.

3. *It is curious why RIG-I/MDA5 double knockout does not enhance gene expression from the CHIKV replicon (Fig. 2J). In contrast, ref 41 described drastic differences in GFP expression from CHIKV replicons between WT and RIG-I/MDA5 double knockout NIH 3T3 cells. Ref 41 compared GFP expression after the infection of cells with packaged viral particles that contain the replicon RNA.*

We thank the reviewer for this insightful observation. In the study by Akhrymuk et al.[2] (i.e ref. 41 in the manuscript), mutations in the CHIKV nsP2 gene abolished host transcriptional shutoff and markedly reduce cytopathic effect without affecting viral RNA replication. As a result, these mutants failed to suppress type I interferon (IFN-I) induction and were sensitive to the IFN-I response. In their study they therefore found that both CHIKV WT and mutants viruses spread efficiently in *RIG-I*$^{-/-}$ *MDA5*$^{-/-}$ NIH 3T3 cells, whereas only CHIKV WT spread in IFN-competent cells.

Importantly, the authors used a fully replicative recombinant CHIKV virus encoding GFP from viral subgenomic promoter while additional subgenomic promoter was inserted to drive the expression of structural genes (see figure below from Akhrymuk *et al.*, showing the schematic representation of CHIKV/GFP used in their study).

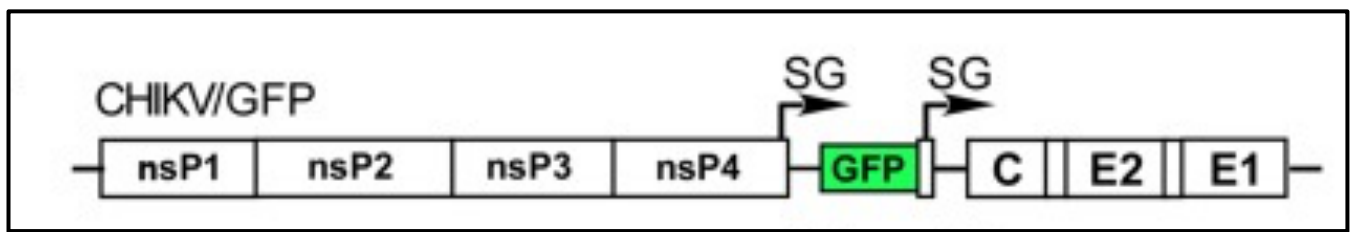

Taken from Figure 6A in Akhrymuk et al.,2019[2]

Thus, viral spread reflected production of infectious particles. In IFN-competent cells, CHIKV nsP2 mutants, lacking the ability to induce a transcriptional shutoff, induced IFN-I, which limited viral spread. This restriction was relieved in *RIG-I*$^{-/-}$ *MDA5*$^{-/-}$ cells that are deficient in IFN-I induction. Notably, despite reduced spread, the authors showed that GFP intensity per infected cell was not significantly altered (see figure below from Akhrymuk *et al.*).

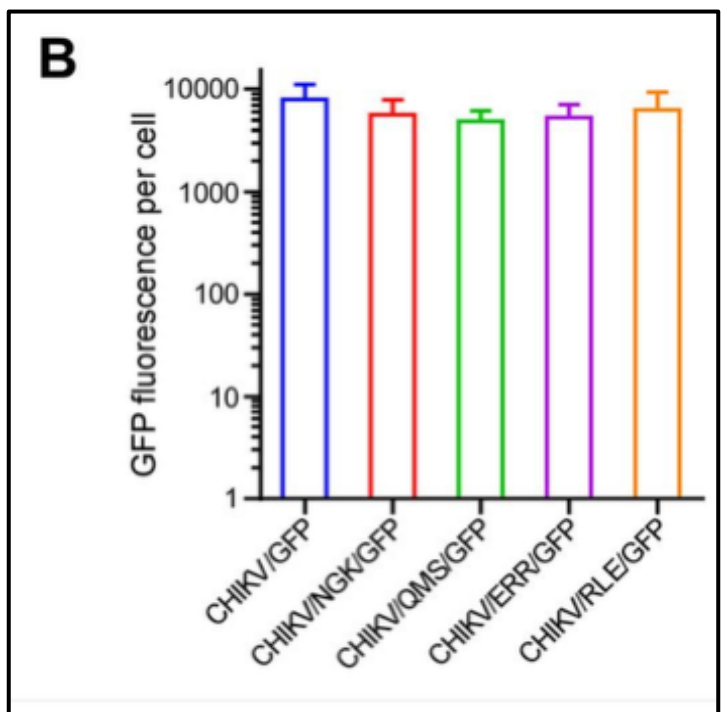

Taken from Figure 7B in Akhrymuk et al.,2019[2]

In contrast, our study employs saRNAs in which structural genes are replaced by GFP gene, precluding assessment of viral spread. Consistent with Akhrymuk *et al.*, we observed no difference in the GFP MFI between WT and *RIG-I*$^{-/-}$ *MDA5*$^{-/-}$ HEK 293 cells (Figure 2J). Moreover, nsP2 proteins encoded by our saRNAs contain the same ATL to ERR mutations identified in their study (named as CHIKV/ERR/GFP), which they found to induce IFN-I but not to reduce GFP fluorescence per cell (see above, Figure 7B from Akhrymuk *et al.*,2019). Consistent with the findings of Akhrymuk *et al.*, we observed an increased percentage of GFP-positive cells in *RIG-I*$^{-/-}$ *MDA5*$^{-/-}$ HEK293 cells (Figure S2F). These data suggest that the interferon response primarily influences the proportion of cells that become permissive to saRNA replication, rather than the magnitude of transgene expression within cells that successfully engage in replication and amplification.

4. *Moreover, a recent study indicates that the productive infection of cultured cells with CHIKV resulted in very low levels of eIF2α phosphorylation as compared to alphaviruses SINV and SFV (Frolova EI et al., J Virol 2023, 97, e0097923-23). Thus, I am wondering whether unlike natural viral infection, direct replicon RNA transfection via Lipofectamine in this work triggers a different innate immune response. This issue can be readily resolved by comparing GFP expression from wildtype and PKR knockout cells infected with the described CHIKV replicons packaged in viral particles.*

We thank the reviewer for highlighting the potential differences between replicon RNA transfection and natural viral infection in shaping innate immune responses, including PKR activation and eIF2α phosphorylation. To address this, we generated CHIKV viral replicon particles (VRPs) containing the saRNAs used in this study. Despite multiple attempts at high multiplicities of infection (MOI), we were unable to efficiently transduce HeLa wild-type and *PKR*$^{-/-}$ cells, precluding a direct comparison of GFP expression under these conditions. See representative data below:

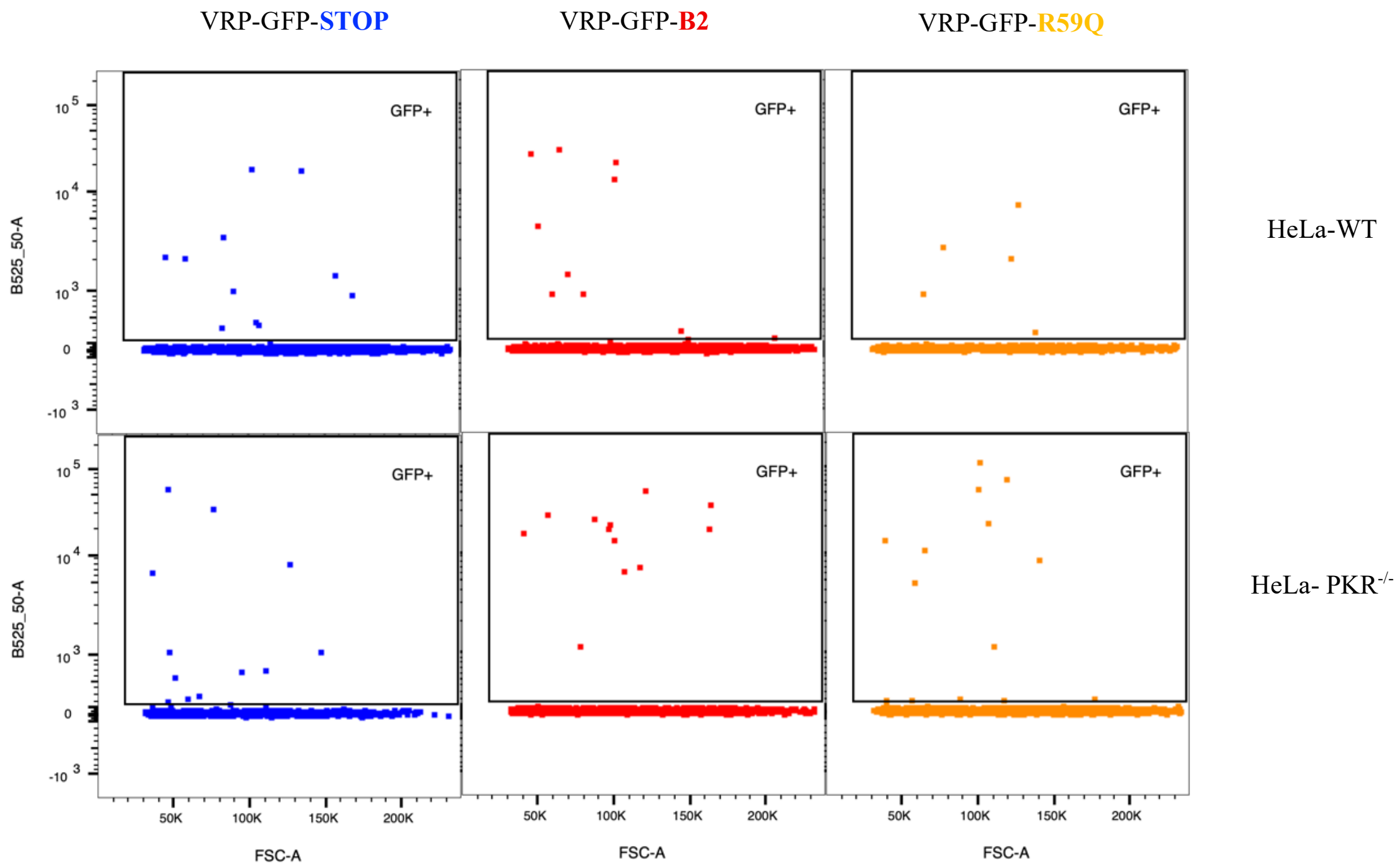

HeLa WT or PKR$^{-/-}$ cells were exposed to CHIKV viral replicon particles (VRPs) carrying CHIKV-GFP-STOP (Blue), -B2^{WT} (red) or -B2^{R59Q} (orange) saRNA (MOI of 10 based on BHK-21 cells). Flow cytometry was used to follow GFP expression 24 hours post-transduction. Dot plots show GFP expression against forward scatter area (FSC-A).

However, in Baby Hamster Kidney-21 (BHK-21) cells, which are highly permissive to VRP transduction, delivery of VRPs encoding NoV B2^{WT} resulted in a robust increase in GFP MFI compared to VRPs lacking functional B2 indicating that NoV B2 enhances CHIKV replicon gene expression in the context of VRP-based delivery (see below). These results suggest that the ability of B2 to enhance replicon-driven gene expression is not restricted to Lipofectamine-mediated RNA transfection. Taken together, these data suggest that B2-mediated enhancement of CHIKV replicon expression is preserved across delivery strategies, while direct comparisons of PKR dependency under infection-based conditions will require future investigation. We have now incorporated this new set of data as part of Figure S2J and S2K (see below), updated the main text (lines 454-463), the Materials and Methods (lines 221-231) and the Figure S2 legend accordingly.

Figure S2 J and S2K:

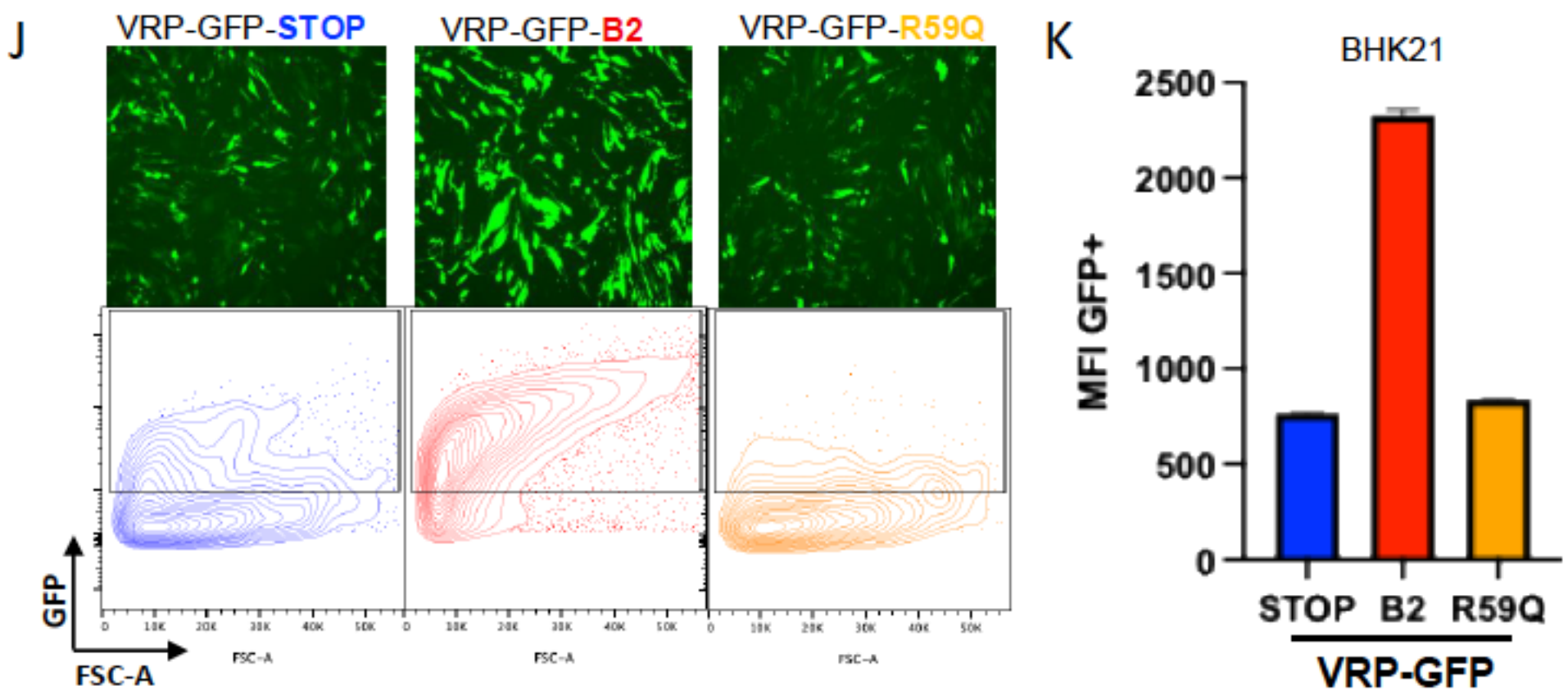

(J) CHIKV-GFP-STOP, CHIKV-GFP-STOP-B2^{WT} or CHIKV-GFP-STOP-B2^{R59Q} saRNAs were packaged into viral replicon particles (VRPs) and used to transduce BHK-21 cells at a multiplicity of infection (MOI) of 10 (shown in the panels as VRP-GFP-STOP, VRP-GFP-B2^{WT}, VRP-GFP B2^{R59Q}). Representative fluorescence microscopy images of transduced cell populations 24 hours post-transduction are shown with corresponding contour plots of GFP^{+} cells displayed below each image. **(K)** MFI of GFP^{+} BHK-21 cells following VRP-mediated delivery of the indicated saRNA analysed by flow cytometry 24 hours post-transduction. Data is a representative of 3 independent experiments.

5. *Further proof-reading is necessary. See below for a few examples:*

- *Line 244: change "50'000" to "50,000";*
- *Line 507: change "form" to "forms";*
- *Line 546: insert "and" before dsRNA;*

We thank the reviewer for identifying these errors and have now corrected them as well as other typos in the text.

Reviewer #2 (Remarks to the Author)

Summary
In 'Tuning intracellular immunity by Nodamura virus B2 protein enhances self-amplifying RNA activity', describe R.Y. Sanchez-David et al. a novel self-amplifying RNA design to boost gene of interest expression. This is a highly relevant development as improved gene of interest expression could lower the initial vaccine dose, reduce costs, and increase vaccine efficiency.
By adding the Nodamura virus B2 gene to two alphavirus-based self-amplifying RNA vectors, the gene of interest expression was increased in a stem cell line and a couple of somatic cell lines. The authors also unraveled insights into the molecular mechanism by showing the involvement of a Dicer-mediated RNA interference and the protein kinase R-driven translational shutdown. Furthermore, confocal images show that the addition of B2 results in retention of the dsRNA in replication spherules at the plasma membrane and binding of B2 to the alphavirus non-structural protein 2. The exact mechanism behind this observation is not discussed in this manuscript.
Overall, this is a valuable study with some limitations in the transfection efficiency of the self-amplifying RNA. Although B2 boosts protein production for the chikungunya virus-based vector, this vector seems less relevant by its low transfection efficiency; only a limited number of cells express the GFP marker. This in contrast to the widely used Venezuelan equine encephalitis virus-based vector which shows a higher percentage of GFP expressing cells. This vector is known for its high gene of interest expression levels, but the addition of the B2 gene shows to increase this expression level further. In addition to a GFP marker, the self-amplifying RNA-induced expression of two antigens is shown as well. I am looking forward to upcoming research in which the efficiency of this novel self-amplifying RNA vector is evaluated in vivo.

Major comments

1. *The authors use a very systematic transfection approach (RNA quantification and integrity check) to make the comparison between the different sa-RNA vectors as reliable as possible. Nevertheless, RNA is delegate and transfection efficiency can vary greatly. In this manuscript, the transfection efficiency also seems to vary between different repeats based on the variation in the percentage GFP positive cells after CHIKV-GFP-B2 transfection in mESCs in Figure S1B and Figure 1E, respectively 15% and 6%. Furthermore, the percentage of GFP positive cells is low in many transfections, e.g. below 4% in HeLa cells. I therefore wonder how reliable the results in Figure 1 and 2 are?*

We thank the reviewer for this important comment. We agree that transfection efficiency is relatively low in some of our experiments and that RNA-based transfection can introduce variability. However, despite variation in the percentage of GFP-positive between experiments, the phenotypes we report are highly reproducible at the level of the MFI across multiple independent biological replicates. The consistency of these effects across repeats supports the robustness of our phenotype. Importantly, in HEK293T cells where transfection efficiency reaches ~20% (Fig. S7B), we consistently observe the same phenotype, i.e that saRNA-mediated expression of NoV B2 enhanced GFP expression (Fig. 4A), indicating that the findings are not driven by rare, transfected cells. Similarly, the use of VEEV-based vectors, which typically yields higher transfection efficiencies, recapitulates the phenotype (Fig. 6A, 6B, 6K, 6L) reinforcing that our findings are not specific to conditions with low transfection rates.

To further address the reviewer's concern, we transfected CHIKV-GFP-STOP, CHIKV-GFP-B2^{WT} or CHIKV-GFP-B2^{R59Q} in highly permissive BHK-21 cells and achieved transfection efficiencies above

50% (revised Figure S2G, see below). Under these conditions, we again observed a significant increase in MFI GFP^{high} in cells transfected with NoV B2-encoding saRNA confirming that the phenotype is maintained even at high transfection efficiency (Figure S2 H-I). We have incorporated this new data in the revised manuscript as part of Figure S2G-I and have updated the figure legend and main text accordingly (lines 454-463).

Figure S2 H-I:

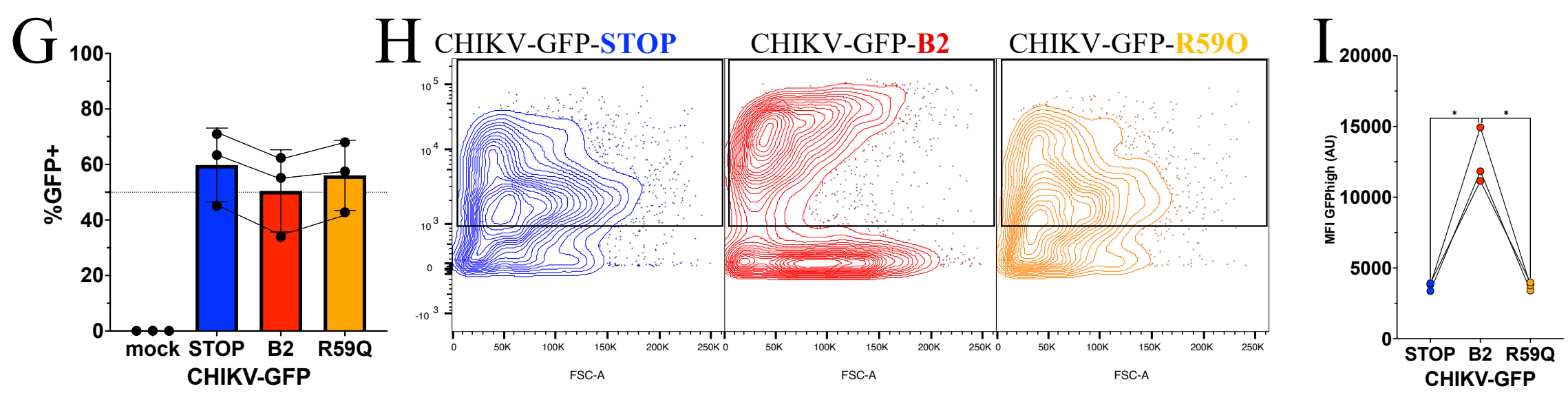

G) Percentage of GFP-positive (% GFP$^+$) BHK-21 cells 24 hours after transfection with CHIKV-GFP-STOP (Blue), CHIKV-GFP-STOP-B2^{WT} (red) or CHIKV-GFP-STOP-B2^{R59Q} (orange) analysed by flow cytometry. Data are means ± s.d. from 3 independent experiments **(H)** Representative contour plots of GFP$^+$ BHK-21 cells transfected with the CHIKV-GFP-STOP (Blue), -B2^{WT} (red) or -B2^{R59Q} (orange) saRNA. **(I)** Histogram showing the MFI of GFP$^+$ BHK-21 cells transfected with the indicated saRNAs. Data are means ± s.d. from 3 independent experiments**.** Statistical analysis was performed using one-way ANOVA followed by Sidak's multiple comparisons test. *P $<$ 0.05.

In addition, we also found that the enhancing effect of NoV B2^{WT} on saRNA-mediated gene expression extends beyond CHIKV and VEEV-derived saRNA and is also observed with Semliki Forest virus (SFV)-derived saRNAs. Transfection of SFV-based saRNA encoding GFP with either wild-type NoV B2^{WT} (SFV-GFP-B2^{WT}), B2^{R59Q} (SFV-GFP-B2 ^{R59Q}) or no B2 (SFV-GFP-STOP) in HEK293, BHK-21, THP-1 and HeLa cells consistently showed enhanced GFP expression in the presence of wild-type NoV B2 compared to B2^{R59Q} mutant or no B2 controls. Furthermore, this enhancement was markedly reduced in HeLa *PKR*$^{-/-}$ cells confirming that it is PKR-dependant. These data have been incorporated in Figure S7A and the corresponding text has been updated (lines 623-629).

Figure S7A:

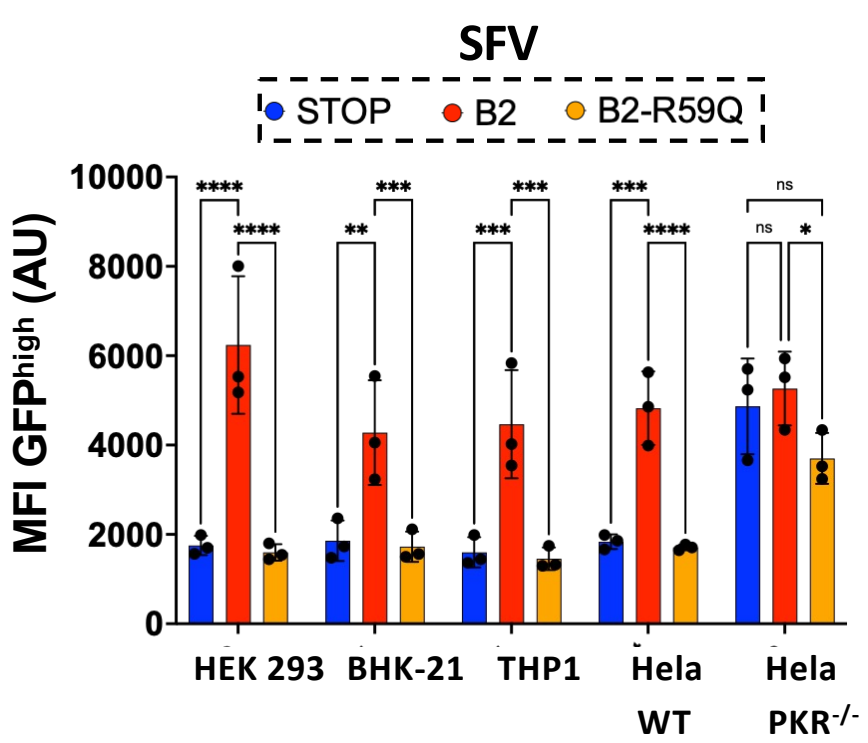

(A) HEK293T, BHK-21, THP-1, and HeLa WT and Hela *PKR*$^{-/-}$ cells were transfected with SFV-GFP-STOP (blue), SFV-GFP-B2^{WT} (red) or SFV-GFP-B2^{R59Q} (orange) saRNA. Flow cytometry was used to monitor MFI of GFP^{high} population 24 hours post-transfection.

Together, these results demonstrate that the enhancement mediated by NoV B2 and the PKR-dependent restriction of saRNA expression represent a consistent phenotype observed across multiple alphavirus saRNA backbones (i.e CHIKV, VEEV and SFV) and cellular contexts. These findings further support the reliability and reproducibility of the data presented in Figures 1 and 2.

2. *In Figure 1, I miss a description of the amount of (independent) repetitions that have been performed for Figure 1B, C, E and F. Are the results consistent over multiple independent repetitions?*

We have now included this information within the figure legend of Figure 1B, C, E, and F.

3. *In Figure 2, comparisons are made in HeLa and HEK293 cells, which both show low percentages of GFP positive cells after transfection (based on Figure 2G and Figure S6). I suggest that the authors add another cell line in which a higher percentage of GFP positive cells is reached, or use another transfection method (e.g. electroporation) to increase the percentage of GFP positive cells.*

We thank the reviewer for this constructive suggestion. To address this concern and as noted above (in our response to comment 1), we first included BHK-21 cells in our analysis. Using cationic lipid-based transfection reagents (i.e. MessengerMAX) we achieved >50% transfection efficiency. Under these high transfection efficiency conditions, we observe the same phenotype as in HeLa cells and HEK293 cells, demonstrating that our conclusions are not dependent on low transfection efficiency (data now incorporated in Fig. S2G-S2I).
In addition, we optimised the transfection conditions in HeLa cells by increasing the amount of saRNAs from 0.1µg to 1µg resulting in more than 10% of GFP-positive cells (see below for rebuttal only the titration experiment using CHIKV-GFP-STOP in HeLa cells, left panel). Using these optimised conditions (1µg of saRNA used per transfection), we recapitulated the enhancing effect of NoV B2 on saRNA-mediated GFP expression (see below, right panel). Transfection of CHIKV-GFP-STOP, CHIKV-GFP-$B2^{WT}$ or CHIKV-GFP-$B2^{R59Q}$ showed that NoV B2 expression enhanced GFP levels compared to saRNA lacking NoV B2 or expressing the NoV $B2^{R59Q}$ mutant. This enhancing effect of NoV $B2^{WT}$ was lost in HeLa *PKR*$^{-/-}$ cells. We have incorporated these new data in Figure 4B and updated the legend as well as the main text (lines 499-504).

A.

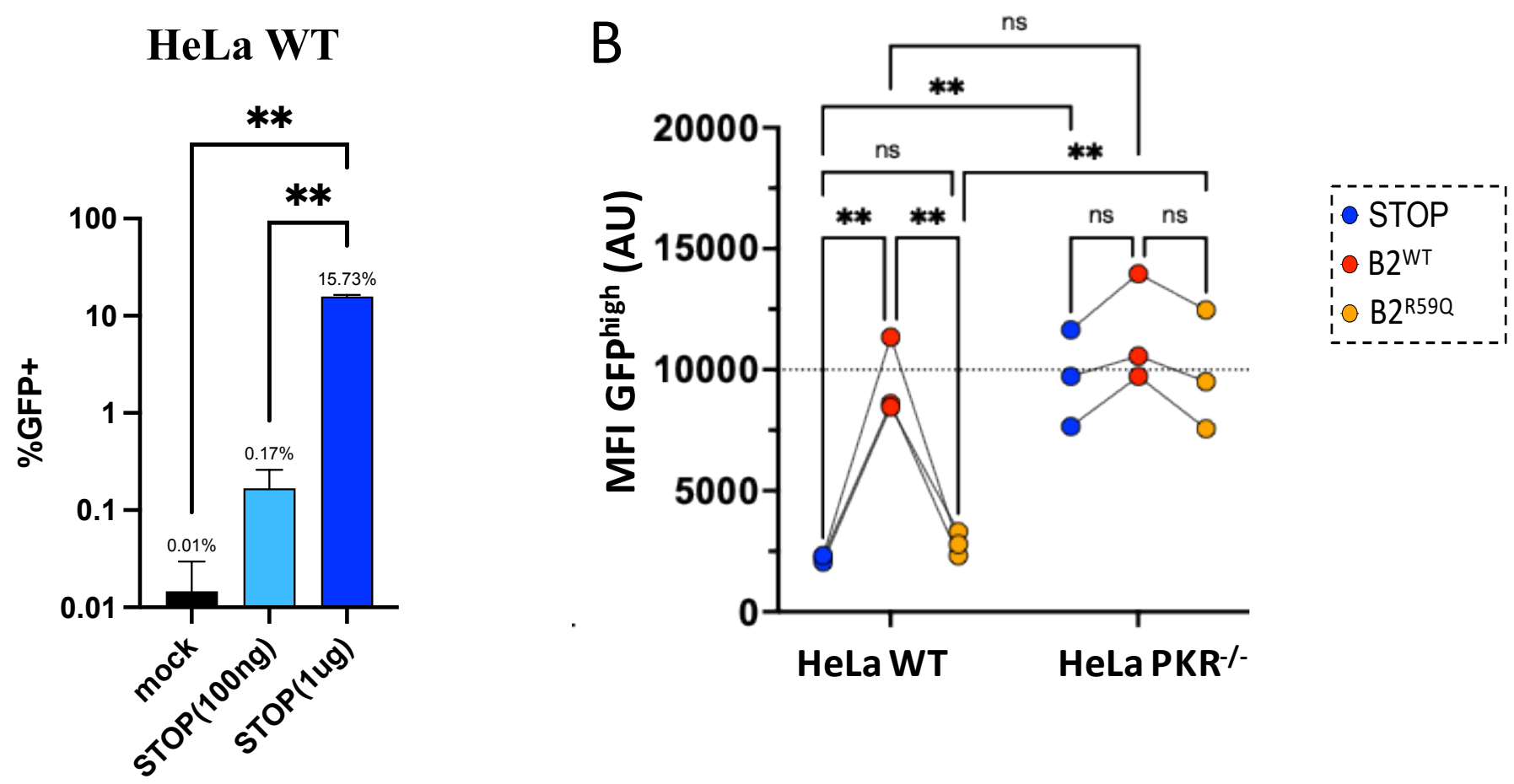

(a) Hela cells were transfected with 0.1 µg or 1µg of CHIKV-GFP-STOP saRNA as indicated. Flow cytometry was used to follow GFP expression. Graph represents the precent GFP^+ cells detected 24 hours after transfection. Data represent the mean ± s.d. of three independent experiments. Statistical analyses were performed using one-way ANOVA with Sidak's multiple comparisons test as post-test for pairwise comparisons. **P < 0.01. (b) Also shown as figure 4B in the manuscript. HeLa-WT and Hela-PKR-/- cells were transfected with 1µg of CHIKV-GFP-STOP (Blue), -$B2^{WT}$ (red) or -$B2^{R59Q}$ (orange). Flow cytometry was used to monitor MFI of GFP^{high} population 24 hours after transfection. Data represent the mean ± s.d. of three independent experiments. Statistical analyses were performed using two-way ANOVA with Sidak's multiple comparisons test as post-test for pairwise comparisons. **P < 0.01; ns, not significant.

4. *Line 402: the authors state that GFP expression is absent for CHIKV-GFP-B2R59Q, but in Figure 2F the MFI GFP high levels are not zero and also in Figure 2G a low level of GFP positive cells is described. If these levels are comparable to background levels, could the authors please visualize that in the graphs of Figure 2F and 2G.*

We agree with the reviewer, the level of GFP expression with CHIKV-GFP-B2^{R59Q} is not completely absent as the level of GFP is above zero. We have therefore revised the sentence in line 432-434 replacing "absent" with "markedly reduced" to more accurately reflect the data.
Lines 430-432 now reads: "Importantly, this enhancement required NoV B2's dsRNA binding ability as GFP expression was markedly reduced with saRNAs encoding NoV B2^{R59Q} (Fig. 2D, 2F)."

5. *Line 403-406: the authors conclude that the higher levels of GFP and nsP1 (+)-sense RNA, observed after CHIKV-GFP-B2WT transfection, are due to the B2 dsRNA-binding ability. However, the percentage of GFP positive cells is also higher for the CHIKV-GFP-B2WT vector compared to the other two vectors. Could this be another explanation for the increased RNA levels, or was the RNA extracted from only GFP positive cells?*

We thank the reviewer for this comment. We agree that differences in the proportion of GFP-positive cells could potentially contribute to the difference observed by RT-qPCR. To address this directly, we performed three independent experiments in which HEK293T cells were transfected with CHIKV-GFP-STOP, CHIKV-GFP-B2^{WT}, and CHIKV-GFP-B2^{R59Q} saRNAs and then GFP-positive cells were sorted by FACS prior to RNA extraction and RT-qPCR analysis. However, slight differences in transfection efficiency between wells could influence RNA levels measured in GFP-positive population. Importantly, GFP-negative cells may still contain input saRNA but fail to support production replication. Therefore, to control for variability in total RNA delivery per well, we also sorted the GFP-negative population from the same wells and incorporated both fractions into the ΔΔCt calculation (see below for a representative gating strategy used for FACS sorting). This approach provides an internal normalisation for transfection efficiency and input RNA levels within each well for each of the three biological replicates.

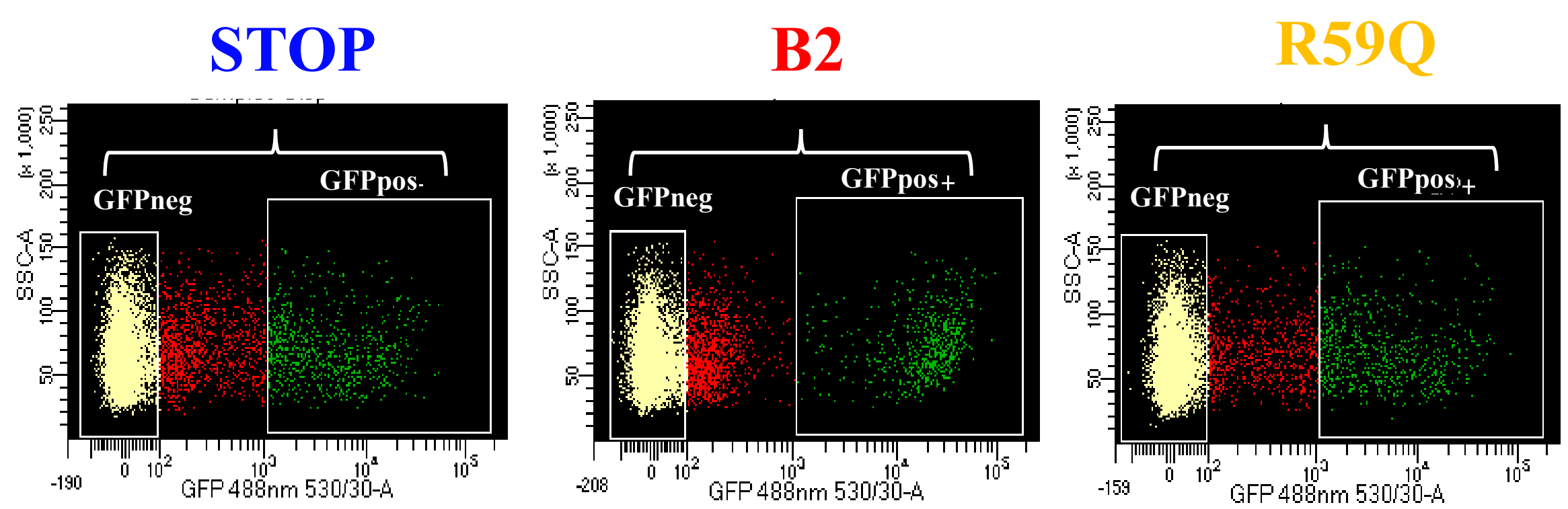

HEK293T cells were transfected with CHIKV-GFP-STOP (Blue), -B2^{WT} (red) or -B2^{R59Q} (orange) saRNA. Dot plots represent the gating strategy used for FACS sorting of GFP$^-$ and GFP$^+$ cells for cells processed in figure S2C.

The ΔΔCt calculation was performed as follows:

$$2^{-\Delta\Delta Ct} = 2^{-[(Ct(saRNA)-Ct(GAPDH))\boldsymbol{GFPpos}]-[(Ct(saRNA)-Ct(GAPDH))\boldsymbol{GFPneg}]}$$

Under these optimised conditions, we again observed an increased in GFP RNA levels (+strand) for the B2^{WT} construct, whereas this enhancement was lost with the R59Q mutant, consistent with a requirement for B2 dsRNA-binding activity. For (-)strand saRNA, no difference was observed between STOP and B2^{WT} construct, while a modest reduction was detected with for the R59Q mutant (see Fig.S2C below). Together, these results indicate that the observed differences in RNA levels cannot be explained by differences in the proportion of GFP-positive cells, but instead reflect specific effects of B2 and its dsRNA-binding activity on +strand saRNA accumulation. In contrast, NoV B2^{WT} does not enhance (-)strand RNA levels compared to the STOP vector, although a modest reduction is observed with the R59Q mutant.

These new data have been incorporated in Fig. S2C, while the original data obtained from bulk (unsorted) cells are retained in Fig.2 H and Fig. S2B. The corresponding figure legends and main text (lines 434-438) have been revised accordingly

Fig. S2C:

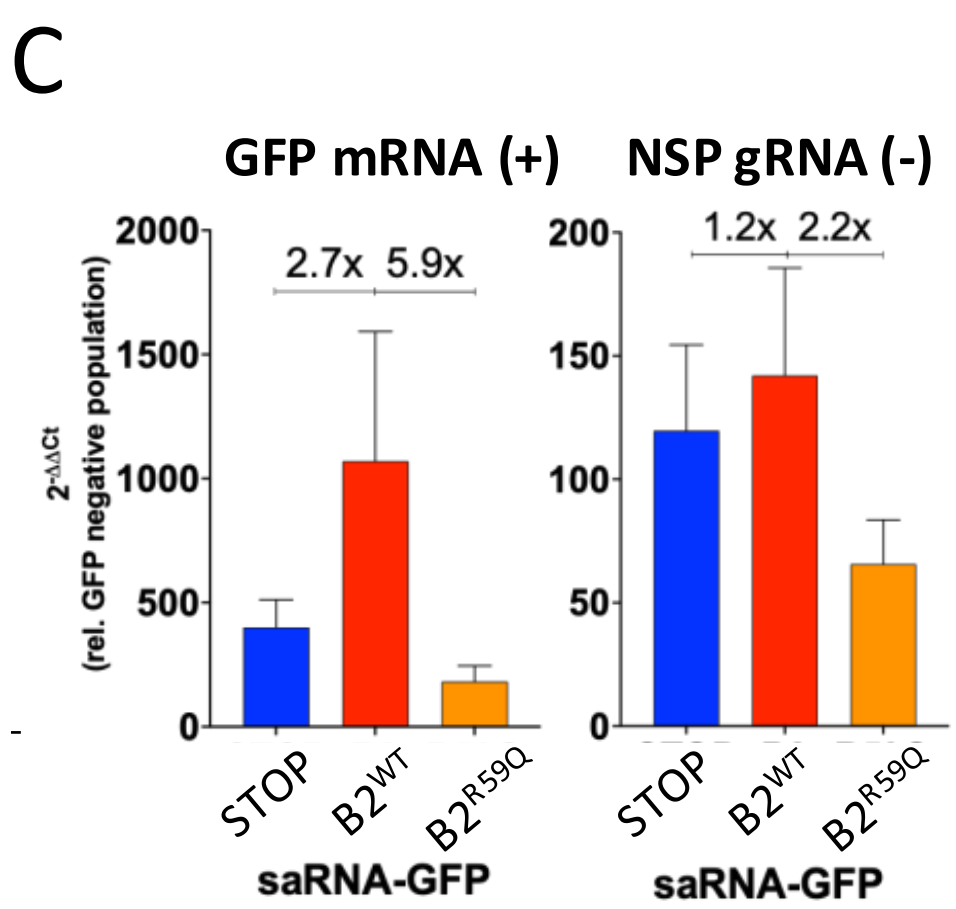

(C) HeLa cells were transfected with CHIKV-GFP-STOP (blue), CHIKV-GFP-B2^{WT} (red) or CHIKV-GFP-B2^{R59Q} (yellow) expressing saRNA. 24 hours post-transfection, GFP^{high} cells were isolated by FACS prior to RNA extraction and RT-qPCR analysis to assess the levels of positive (+)- strand GFP mRNA (left) or CHIKV negative (-)-strand RNA levels using primers targeting nsP1 region (right).

6. *Line 446-448: Figure 4A shows that the GFP expression levels in HEK293T Dicer-/- PKR-/- were similar for all three constructs. The CHIKV-GFP-STOP and CHIKV-GFP-B2R59Q are higher, while the CHIKV-GFP-B2WT levels are lower than in the other three described cell lines. Is there an explanation why the CHIKV-GFP-B2WT GFP expression drops in this cell line?*

We thank the reviewer for this insightful comment. As noted, GFP expression driven by CHIKV-GFP-B2^{WT} is reduced in HEK293T *Dicer* $^{-/-}$ *PKR* $^{-/-}$ compared with HEK293T WT and *Dicer*$^{-/-}$ (clone 2-20) cells. This reduction was not observed in HEK293 *RIG-I*$^{-/-}$ *MDA5*$^{-/-}$ relative to WT cells (Figure 2J) nor in HeLa *PKR* $^{-/-}$ cells compared to HeLa WT cells (see revised Fig.4B and response to reviewer 1 comments 1 and 2), suggesting that the reduced GFP levels in HEK293T *Dicer*$^{-/-}$ *PKR*$^{-/-}$ reflect a clone-specific effect rather a general consequence of PKR loss. The *Dicer*$^{-/-}$ *PKR*$^{-/-}$ cells were kindly provided by Prof. Bryan Cullen and were originally generated by knocking out PKR in a pre-existing Dicer $^{-/-}$ clone, clone 4-25[1] . In our initial experiments, the *Dicer*$^{-/-}$ line used corresponded to the clone 2-20, the other independent *Dicer*$^{-/-}$ clone described in Kennedy *et al.*, 2015[1] . To test whether the reduced GFP levels were clone-specific, we included *Dicer*$^{-/-}$ clone 4-25 in our experiments and observed a reduced GFP expression driven by CHIKV-GFP-B2^{WT} consistent with the GFP level seen in the *Dicer*$^{-/-}$ *PKR*$^{-/-}$

cells. Despite this overall reduction, CHIKV-GFP-B2^{WT} still exhibited higher GFP expression than CHIKV-GFP-STOP and CHIKV-GFP-B2 ^{R59Q} in clone 4-25 (see below revised Figure 4A) .
These results indicate that the lower GFP levels in *Dicer*$^{-/-}$ *PKR*$^{-/-}$ line reflect properties of the parental clone 4-25, while the enhancing effect of NoV B2 on saRNA-mediated GFP expression remains detectable in this clone but is lost upon PKR knockout. We have revised Figure 4A to include the clone 4-25 data and updated the figure legend to indicate that the HEK 293T *Dicer*$^{-/-}$ *PKR*$^{-/-}$ line was originally derived from clone 4-25 with a reference to the corresponding studies. We have also updated the main text accordingly (lines 490-499).

Figure 4A:

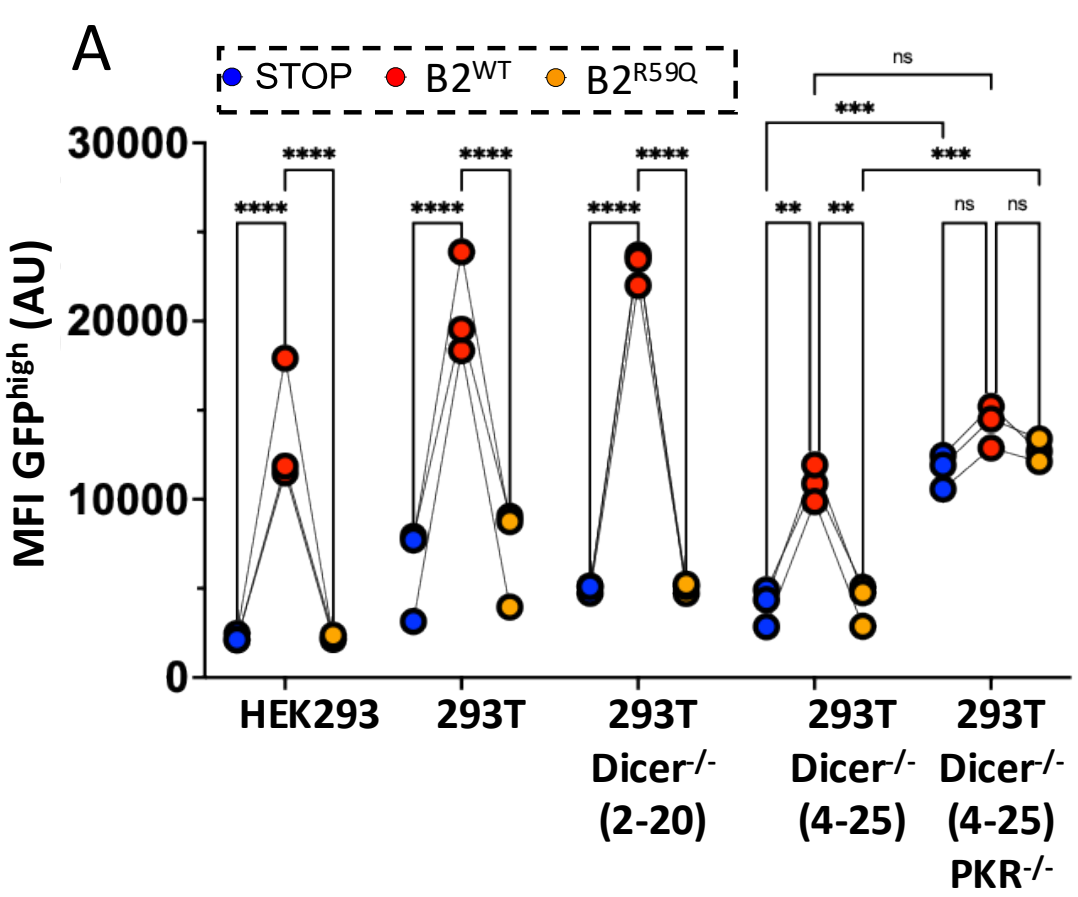

HEK293 and HEK293T as well as HEK293T *Dicer*$^{-/-}$ (clone 2-20 and clone 4-25) and HEK293T *Dicer*$^{-/-}$ *PKR*$^{-/-}$ cells (derived from the parental *Dicer*$^{-/-}$ clone 4-25[1,31]) were transfected with CHIKV-GFP-STOP (Blue), -B2^{WT} (red) or -B2^{R59Q} (orange). Flow cytometry was used to monitor MFI of GFP^{high} population at 24 hours after transfection. Data represent the mean ± s.d. of three independent experiments. Statistical analyses were performed using two-way ANOVA with Sidak's multiple comparisons test as post-test for pairwise comparisons. ***$P < 0.001$; ns, not significant.

7. *Line 479-481: the authors describe that the P-PKR levels in the THP1 transfected cells are similar for CHIKV-GFP-B2R59Q and CHIKV-GFP-B2WT, but that is not very clear from the western blot (vague bands and shadows). Can the authors provide a clearer western blot for the PKR and P-PKR detection? Perhaps the antibody is just too bad to improve the western blot and the authors can show in another way that the PKR and P-PKR levels are similar.*

We agree that the original western blot did not clearly demonstrate the comparable PKR and P-PKR levels between CHIKV-GFP-B2^{R59Q} and CHIKV-GFP-B2^{WT} conditions. In response, we have repeated the experiment and carried out the western blot using optimised sample preparation and exposure settings to improve the signals. The new blot shows improved band clarity for both PKR and P-PKR, allowing to better compare between conditions. We confirmed that P-PKR levels are comparable in THP1 transfected with CHIKV-GFP-B2^{WT} and CHIKV-GFP-B2^{R59Q}. However, differences were observed at the level of eIF2α phosphorylation: P- eIF2α levels were increased in cells transfected with CHIKV-GFP-B2^{R59Q}, whereas P- eIF2α remained at levels comparable to non-transfected cells when CHIKV-GFP-B2^{WT} was used. These results demonstrate that NoV B2^{WT} inhibits the phosphorylation of eIF2α, while this inhibitory effect is lost with the NoV B2^{R59Q} mutant.
We have replaced the original western blot with this higher-quality immunoblot in the revised manuscript (see below revised Figure 4F).

Figure 4F:

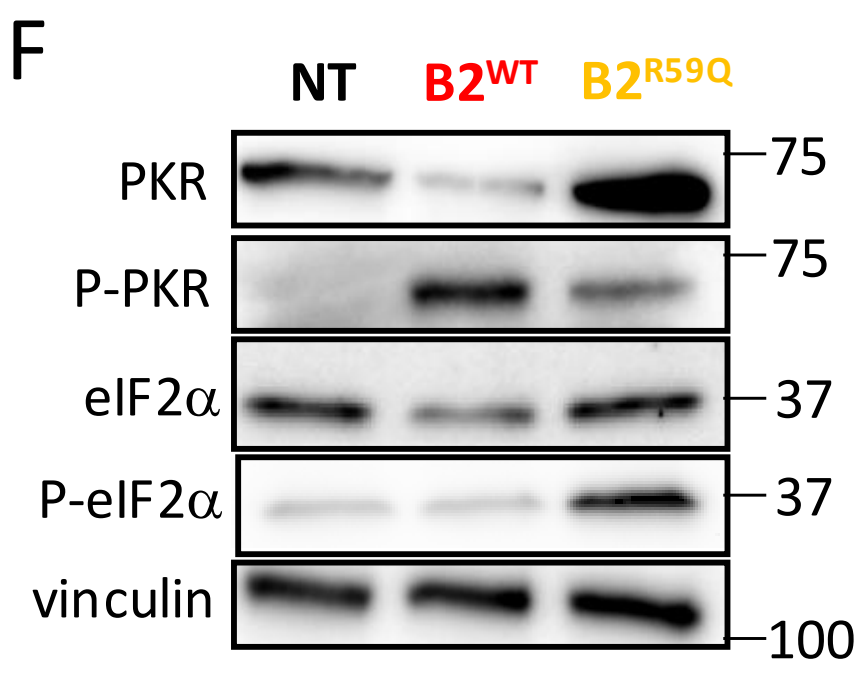

Western blot analysis of THP-1 cells 24 hours post-transfection with CHIKV-GFP-saRNA expressing indicated B2 proteins. Vinculin served as a loading control.

8. *Line 481-484: based on Figure 4H, I would not state that P-eIF2α is absent in CHIKV-GFP-B2WT transfected cells, and present in CHIKV-GFP-B2R59Q transfected cells. Both panels show equally vague levels of P-eIF2α, whereas the results from Figure 4F and 4G are more convincing.*

We thank the reviewer for this careful observation and agree that, in the originally submitted Figure 4H, the levels of P-eIF2α were difficult to interpret due to the overall image quality. To address this concern, we have repeated the experiment and improved the immunofluorescence analysis by including actin staining as an additional cytoplasmic marker to better delineate cellular morphology and signal localisation. In the updated figure (see below, revised Figure 4H), cells transfected with CHIKV-GFP-$B2^{R59Q}$ show limited GFP expression while displaying a P-eIF2α signal, whereas cells transfected with CHIKV-GFP-$B2^{WT}$ exhibit strong GFP expression and no detectable P-eIF2α staining. These observations are now more clearly appreciable and are consistent with the conclusions drawn from Figures 4F and 4G. We have replaced Figure 4H with the improved microscopy data and updated the corresponding figure legend accordingly.

Revised Figure 4H:

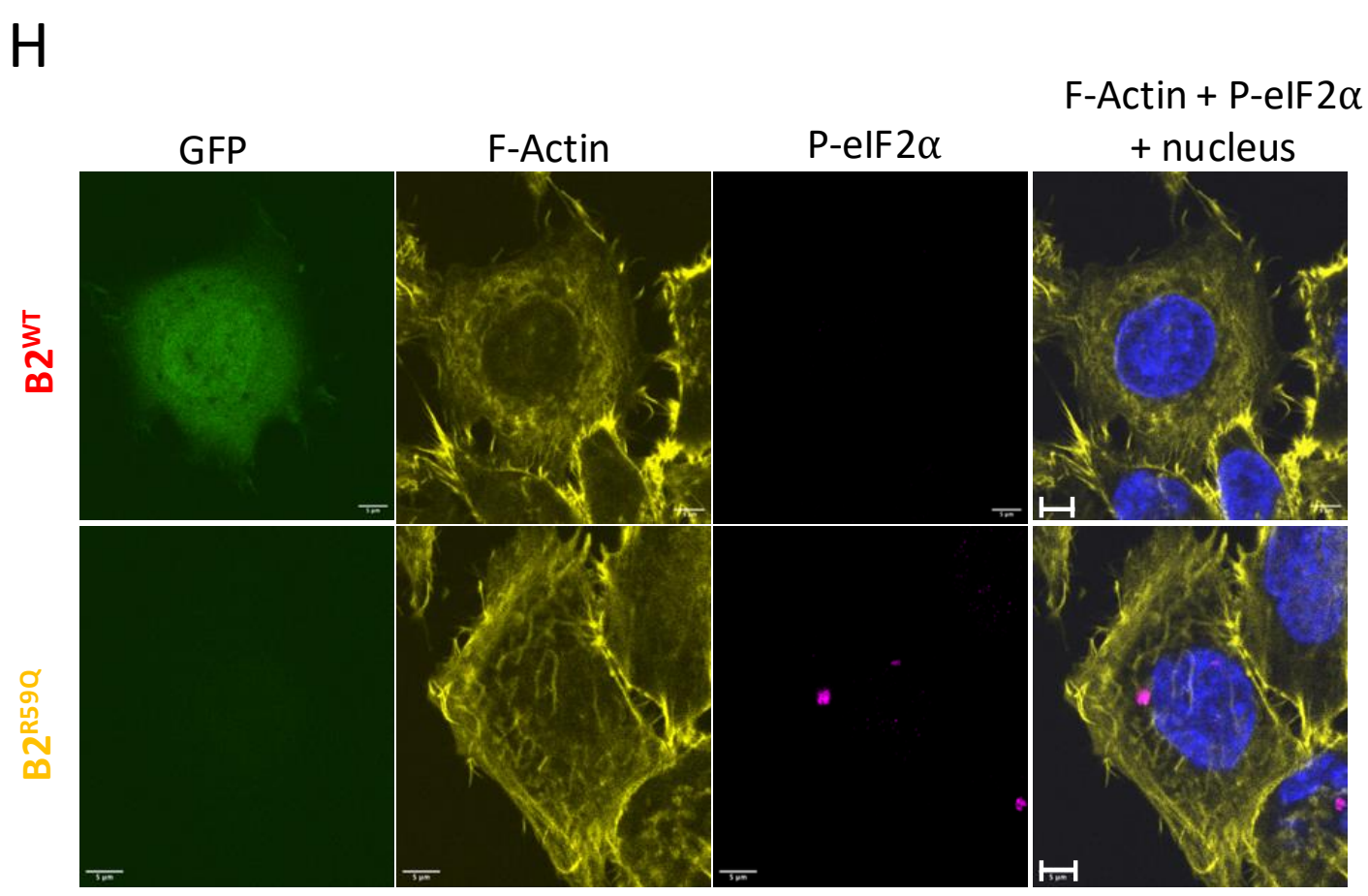

Representative images HeLa cells transfected with corresponding CHIKV-GFP-$B2^{WT}$ or -$B2^{R59Q}$ saRNA for 24 hours and analysed by confocal microscopy. Cells were stained for P-eIF2α (magenta), F-actin (yellow), nuclei (blue); GFP fluorescence (green) indicates saRNA-mediated expression. Scale bars: 5 µm.

9. *Line 515-516: visually there seems a difference between the B2 distribution of the CHIKV-GFP-B2R59Q and CHIKV-GFP-B2WT in Figure 5 and Figure 5S, however cell markers are lacking to conclude that NoV B2WT localizes to the periphery and B2R59Q throughout the cytoplasm. I suggest that the authors add a panel of stains to show co-localization with the periphery and the cytoplasm, or at least of the nucleus as in Figure 6C and D.*

We thank the reviewer for this constructive comment. In response, we performed a time-course analysis at 8, 12 and 24 hours post-transfection including NucleoSpot and Phalloidin stainings to label nuclei and F-actin, respectively, and thereby improve visualisation of cell morphology and the cytoplasmic compartment. Across all analysed time-points, visualisation of the actin cytoskeleton confirmed that the GFP signal encoded by the saRNA is distributed throughout the entire cell and can therefore be reliably used to define the cellular mask for image analysis. Using this approach, we observed that STrEP-tagged NoV B2^{WT} shows peripheral enrichment as early as 8 hours post-transfection and this peripheral localisation becomes progressively more pronounced at later time points (12 and 24 hours). In contrast, STrEP-tagged NoV B2^{R59Q} displays a more uniform cytoplasmic distribution at all analysed time-points. We have now included representative images from these time-points showing co-straining of STrEP-tagged NoV B2 (WT or R59Q) with a nuclear staining (NucleoSpot) and Phalloidin in Figure S5A (see below). We have also revised the main text to include these experiments (lines 573-577).

Revised Figure S5A.

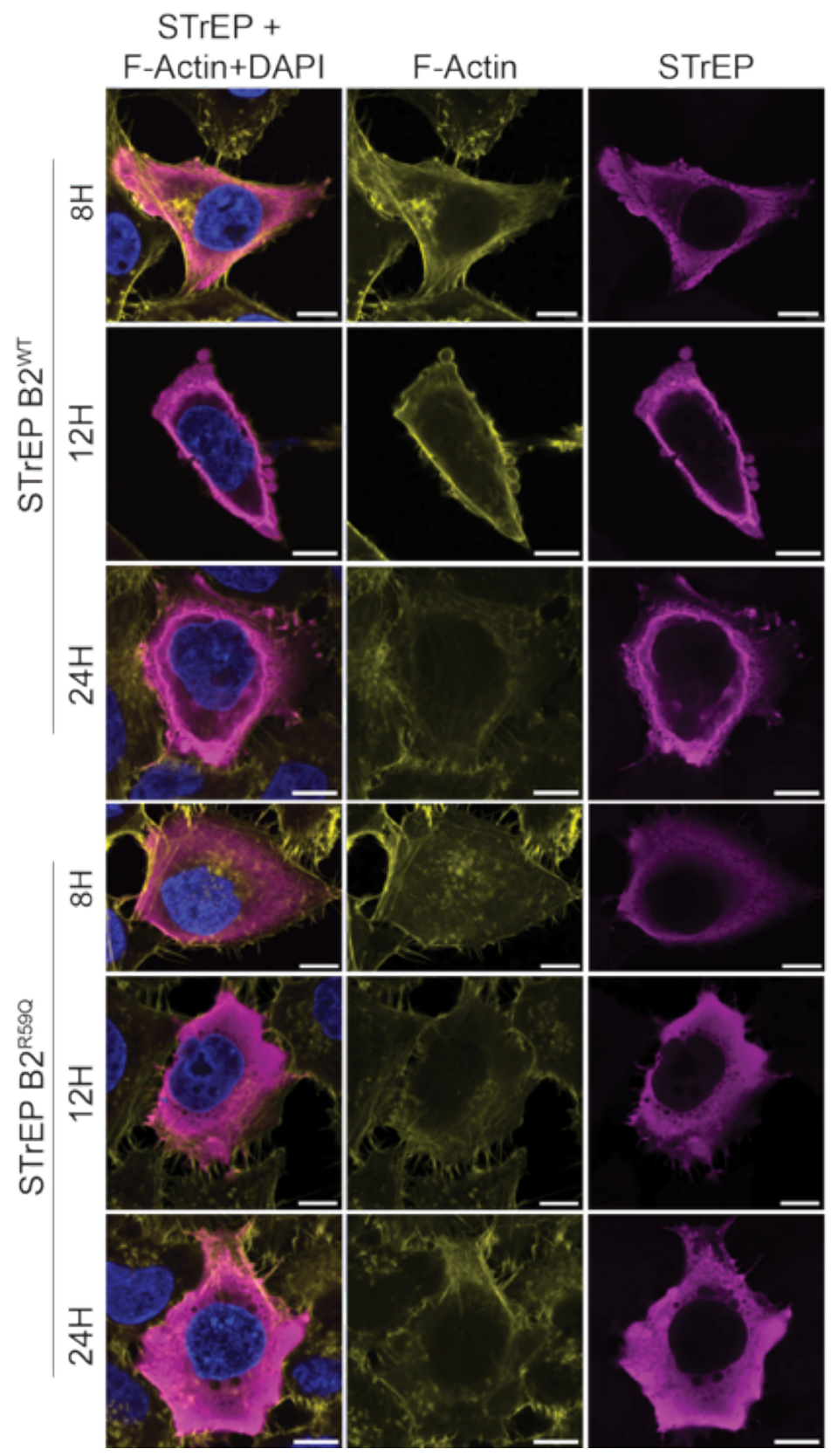

HeLa *PKR*$^{-/-}$ cells were transfected with CHIKV-GFP saRNA encoding STrEP-B2^{WT} or STrEP-B2^{R59Q} and analysed by confocal microscopy at 8, 12 and 24 hours post-transfection. Cells were stained for STrEP tag (magenta), nuclei (blue) and F-actin (yellow).

10. *Line 518: I assume that the yellow color in Figure S5A indicates overlap between B2 and GFP in the cytoplasm. Based on this, I think it is incorrect to state in line 518 that there is 'no overlap with GFP'.*

We agree with the reviewer that the merged image in the original Figure S5A (which has now been moved to Fig.S5B and S5C) shows a degree of overlap between NoV B2 and GFP signals (appearing yellow in the merged image), We have therefore revised the main text to more accurately reflect the microscopy data. The sentence (lines 578-580) now reads:

"Similar results were obtained in *Ifnar2*$^{-/-}$ THP-1 cells where NoV B2^{WT} localised predominantly at the cell periphery and showed limited overlap with GFP, whereas NoV B2^{R59Q} exhibited greater co-localisation with cytoplasmic GFP (Fig. S5B, S5C)".

11. *Alphaviruses are known to internalize their dsRNA-containing spherules in type I cytopathic vacuoles (CPV-I) (Girard et al. Journal of Virology 2024). It is therefore very interesting that the dsRNA does not internalize in the presence of B2, and keeps located at the cell periphery (Figure 5 and 6). Based on this, I have some questions and comments:*
    - *11.1 At which time point post transfection were the cells in Figure 5A-D and Figure 6C-D stained, and is there a difference with later time points, e.g. does relocation of the dsRNA take place?*

We thank the reviewer for this interesting question. All the microscopy images shown in Figure 5A-D and Figure 6C-D were acquired at 20 hours post-transfection of saRNAs into HeLa WT or *PKR*$^{-/-}$ cells. We have now clarified this information in the corresponding figure legends. To note, internalization of spherules and formation of CPV-I varies greatly between alphaviruses being prominent for SFV but much more modest, or even absent, for other alphaviruses including CHIKV; the determinant responsible for these differences has been mapped to junction between alphavirus unique domain and hypervariable domain of nsP3[3]. Our data aligns with this finding.

To directly address the reviewer's comment regarding potential dsRNA relocalisation at later time points, we performed a time-course experiment in which we transfected HeLa PKR $^{-/-}$ with CHIKV-GFP-B2^{WT} and CHIKV-GFP-B2^{R59Q} saRNAs. Cells were stained for dsRNA, F-actin, nuclei together with GFP imaging at 36 hours post-transfection. At this time point we could observed a similar distribution pattern to earlier time points: in NoV B2^{WT}-expressing cells, dsRNA remained predominantly at the cell periphery, whereas in NoV B2^{R59Q}-expressing cells, more of the dsRNA reside in the body of the cell. These new data have been incorporated in Figure S6B (see below).

Figure S6B:

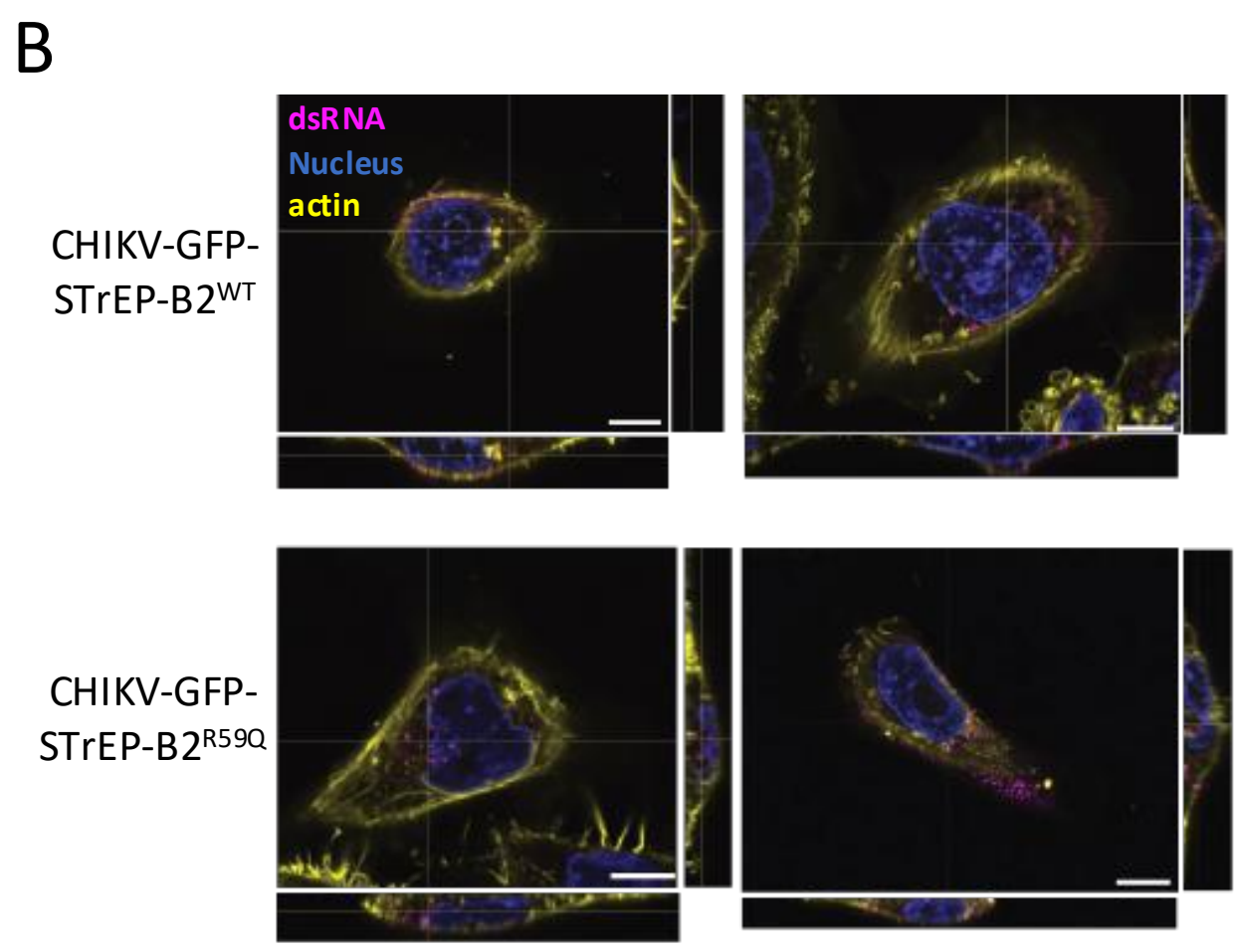

Representative images of HeLa *PKR*$^{-/-}$ cells transfected with CHIKV-GFP-STrEP-B2^{WT} or -STrEP-B2^{R59Q} for 36 hours. Cells were stained for dsRNA (magenta), nuclei (blue) and F-actin (yellow). Scale bar is 10 µm. All images are lined with orthogonal views showing distribution of STrEP signal, F-actin and dsRNA.

- *11.2: Is the cytoplasmic dsRNA upon CHIKV-GFP-STrEP-B2R59Q transfection a result of the spherule internalization or do the authors believe that this is free floating dsRNA (as visualized in the graphical abstract)? A staining of dsRNA and nsP1 could create some more clarity.*

We thank the reviewer for this insightful comment. To address whether the cytoplasmic dsRNA observed upon CHIKV-GFP-STrEP-B2^{R59Q} transfection remains associated with replication structures or represents free cytosolic dsRNA, we performed co-staining for nsP1 and dsRNA, together with F-actin and nuclei. In cells transfected with CHIKV-GFP-B2^{R59Q} saRNA, we observed nsP1-enriched cytoplasmic structures containing dsRNA, consistent with alphavirus replication compartments such as type I cytopathic vacuoles (CPV-I). However, we also detected dsRNA signal in regions lacking nsP1, indicating that dsRNA is not exclusively confined to these structures and is also present in the surrounding cytoplasm.

These new data have been incorporated into Figure S6C (see below), the main text (lines 589-595) has been revised accordingly and the graphical abstract (now incorporated in Fig. S8) has been updated.

Figure S6C:

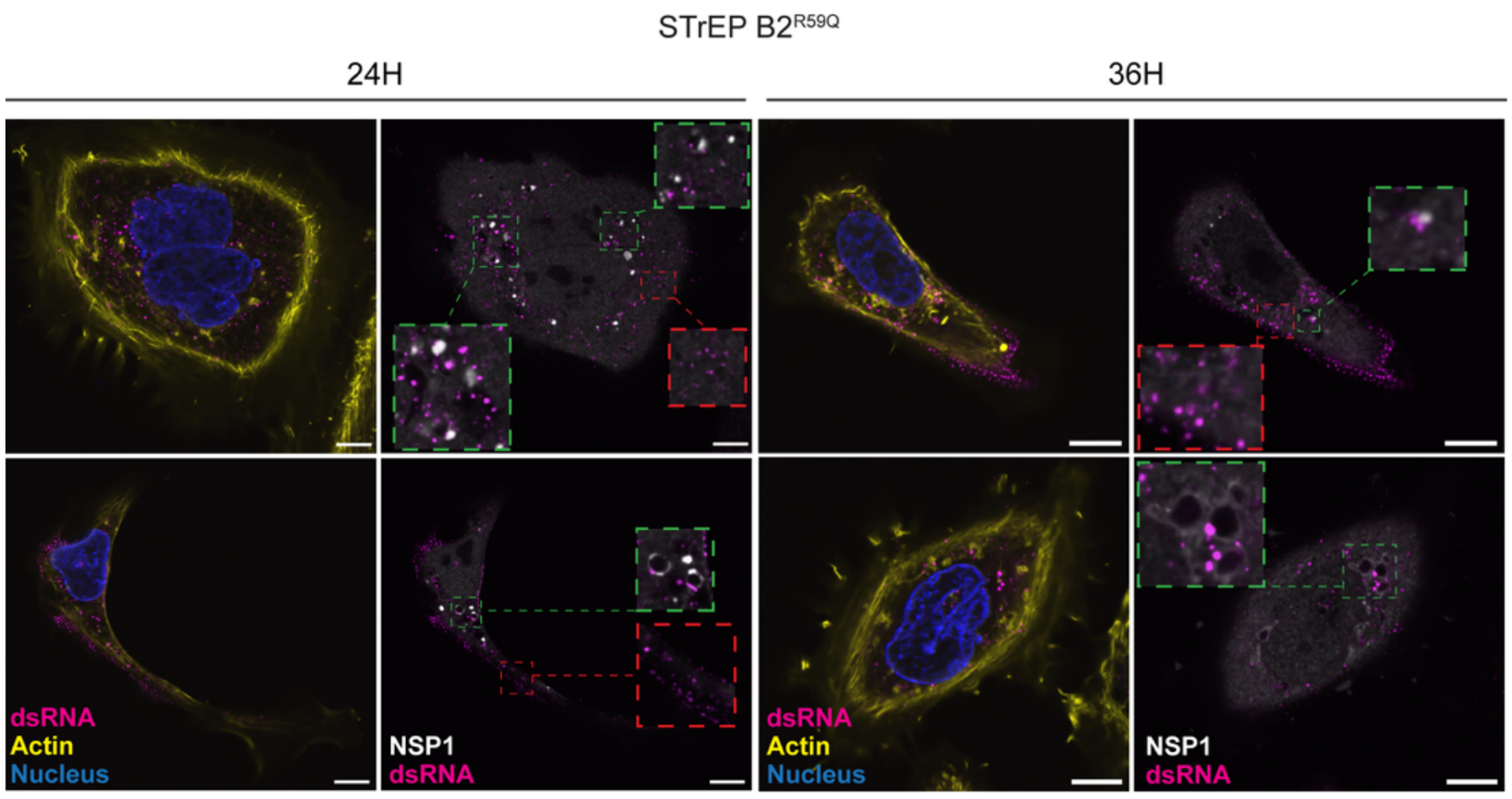

(C) Representative images of HeLa *PKR*$^{-/-}$ cells transfected with CHIKV-GFP-STrEP-B2 ^{R59Q} for 24 hours or 36 hours. Cells were co-stained for nsP1 (white), dsRNA (magenta), F-actin (yellow) and nuclei (blue). Insets show magnified views with green dashed squares indicating dsRNA in nsP1-enriched cytoplasmic structures and red dashed squares indicating dsRNA in regions lacking nsP1. Scale bars: 10 µm.

- *11.3 Does this lack of internalization alleviate PKR restriction, or creates B2 a sort of protective layer? Do the authors have a theory for this, and can they provide experimental evidence?*

We thank the reviewer for raising these important questions regarding the relationship between NoV B2 localisation and alleviation of PKR restriction. Our results support a model in which B2 enhances saRNA-mediated gene expression and alleviates PKR-mediated restriction through a combination of spatial and molecular mechanisms. NoV B2 expressed in cis from saRNA alleviates PKR-mediated inhibition by preventing eIF2α phosphorylation, resulting in enhanced transgene expression. Confocal microscopy in B2^{WT}-expressing cells revealed that B2 accumulates at the cell periphery, forming a layer beneath dsRNA foci, which themselves are more enriched at the periphery compared to cells expressing

the dsRNA-binding mutant NoV $B2^{R59Q}$. In contrast, in $B2^{R59Q}$-expressing cells, dsRNA is more broadly dispersed throughout the cytoplasm and is detected both in nsP1-enriched cytoplasmic structures and in regions lacking nsP1, consistent with dsRNA that is no longer confined to replication structures. To note, our analysis cannot confirm is the dsRNA diffusely present in cytoplasm viral dsRNA, its degradation products or replicase generated dsRNA of non-viral origin that is also present in cells containing active alphavirus replicase[4].

Based on these observations, we propose that B2 promotes the retention and/or stabilisation of dsRNA within spherules at the plasma membrane. Alphavirus replication complexes initially form at the plasma membrane before being internalised into CPV-I in some alphaviruses[5,6]. In this context, B2 peripheral localisation and the associated enrichment of dsRNA at the cell periphery are compatible with a model in which B2 increases the residence of dsRNA within membrane-associated spherules, and/or prevents different dsRNAs generated by replicase proteins to enter cytosol thereby limiting their exposure to PKR. B2 may act as a membrane-proximal "protective layer" that stabilises dsRNA at peripheral replication sites and reduces its accessibility, rather than necessarily preventing internalisation per se. We note that dsRNA binding alone is unlikely to fully explain the protective effect of B2, as other dsRNA-binding proteins do not recapitulate the enhancement of saRNA expression, suggesting that additional properties of B2, such as its localisation and/or interactions with nsP2, a component of replicase complex, also contribute to this activity. The precise mechanistic link between B2 localisation, dsRNA distribution and PKR restriction will require further investigation.

12. *Line 137-138 'B2 expression causes saRNA-derived dsRNA to localize to the cell periphery' and line 549 'B2 can alleviate this restriction by spatially redistributing the dsRNA', suggest that it is the B2 protein that causes the localization of the dsRNA at the plasma membrane, whereas I think it would be more correct to say that B2 prevents the re-localization of the dsRNA to the cytoplasm.*

We agree with the reviewer's suggestion and we have revised the text accordingly.

Lines 141-142 now read: "Notably, B2 expression limits the cytoplasmic re-localisation of the saRNA-derived dsRNA and is associated with its accumulation at the cell periphery."

Lines 637-639 now read "Thus, VEEV-derived vectors, like CHIKV-based saRNAs, are restricted by PKR, and NoV B2 limits the redistribution of the dsRNA to the cytoplasm."

We believe that this revised wording more accurately reflects our data and thank the reviewer for highlighting this important point.

13. *Line 578: it was shown before that transfection of VEEV and CHIKV saRNA vectors in HeLa cells results in a low percentage of cells expressing the gene of interest. Did the authors compensate for potential differences in transfection efficiency that could result in higher overall Spike production? And is the same trend observed in other cell lines with higher percentage of gene of interest expressing cells?*

We thank the reviewer for this important point and agree that differences in transfection efficiency could potentially influence the overall SARS-CoV-2 Spike expression.
For most experiments, we used saRNA constructs encoding GFP followed by a self-cleaving P2A peptide and NoV B2. This design allowed us to gate specifically on GFP-positive cells (MFI higher

than 10^3) and analyse GFP expression at the single-cell level by flow cytometry. By restricting the analysis to the GFP-positive population, we directly assessed expression per transfected cell, thereby minimising the impact of potential differences in transfection efficiency between conditions. Within this gated population, expression of NoV $B2^{WT}$ consistently resulted in increased GFP MFI compared to CHIKV-GFP-$B2^{R59Q}$ or CHIKV-GFP-STOP. Importantly, as requested by this reviewer in Comment 5, we further quantified GFP mRNA levels in sorted GFP-positive HEK 293T cells. We observed increased GFP mRNA in cells transfected with CHIKV-GFP-$B2^{WT}$ compared to CHIKV-GFP-$B2^{R59Q}$ or CHIKV-GFP-STOP. This additional analysis supports that NoV B2 enhances transgene expression from the subgenomic RNA independently of transfection efficiency.
For the western blot analyses shown in Figure 6N and performed in HeLa cells, the SARS-CoV-2 Spike coding sequence was inserted in place of GFP with NoV B2 encoded either upstream or downstream of Spike. We acknowledge that western blot analysis represents a bulk readout and does not by itself distinguish between increased transfection efficiency and increased expression per transfected cells. However, equal amounts of saRNA were transfected in all conditions and IFIT1 levels were comparable across samples supporting similar RNA input and innate immune activation. Notably, positioning NoV $B2^{WT}$ upstream of the Spike gene resulted in higher Spike protein expression compared to positioning NoV $B2^{WT}$ downstream, whereas NoV $B2^{R59Q}$ has no effect regardless of the position.
To further address whether this trend is reproduced in a more permissive cell line, we repeated the experiment in HEK 293T cells, which consistently display higher transfection efficiency (approx. 20%, see Fig. S7B). As observed in HeLa cells, positioning NoV $B2^{WT}$ upstream of the Spike gene resulted in increased Spike expression compared to downstream positioning, whereas position of NoV $B2^{R59Q}$ had no effect. We have now included this new data in Figure S7F (see below) and revised the text accordingly (lines 673-676).

Figure S7F:

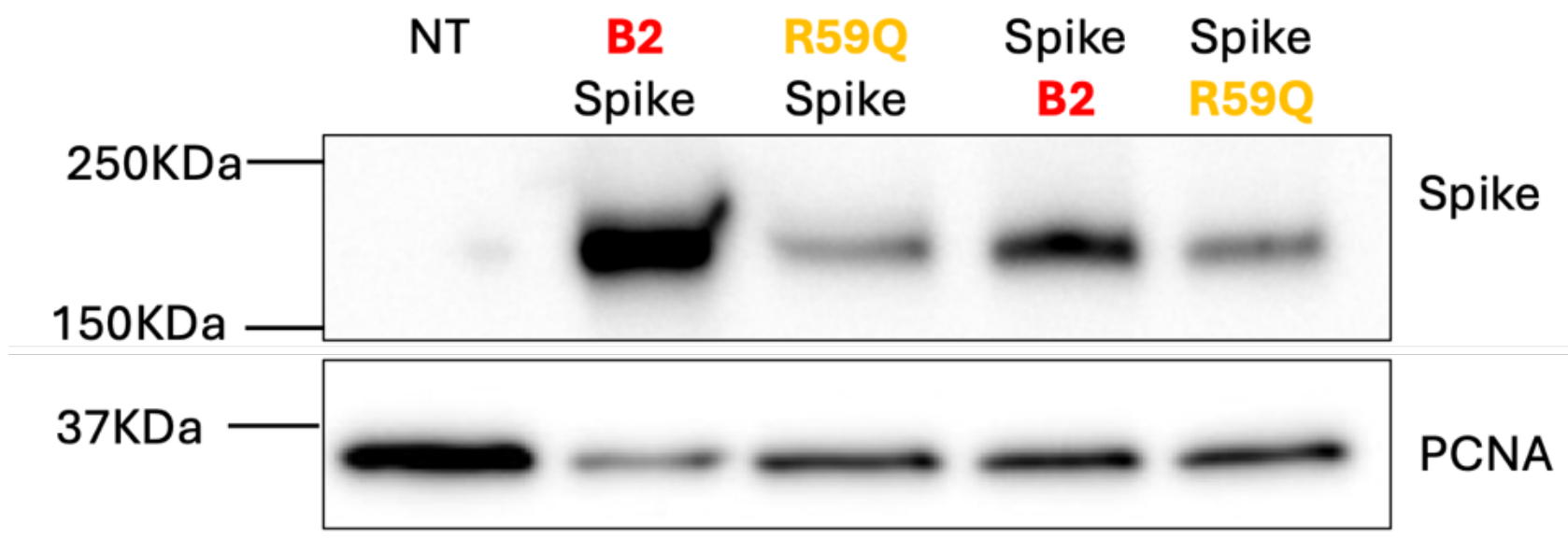

HEK293T cells were transfected with CHIKV saRNAs expressing NoV-$B2^{WT}$ (B2, red) or NoV-$B2^{R59Q}$ (R59Q, orange) upstream or downstream of SARS-CoV-2 Spike prefusion protein. Protein lysates were prepared 24 hours post-transfection and analysed by western blotting using the indicated antibodies. PCNA served as a loading control.

14. *Line 683: the potential reduced cytotoxicity could further boost gene of interest expression as the cell might be able to longer produce the protein. This has an advantage, but could this also cause problems in overproduction of the gene of interest or potential overstimulation of the immune system?*

We thank the reviewer for this thoughtful comment. We agree that prolonged cell viability and sustained antigen expression could, in principle, influence the magnitude or duration of immune activation, particularly in vivo and depending on antigen properties, dose and delivery context. Enhanced and prolonged replication of saRNA may theoretically modulate innate immune engagement through

sustained production of immunostimulatory RNA intermediates (e.g. dsRNA). However, in our experimental system we did not observe an increased type I interferon response upon B2-mediated enhancement of transgene expression as assessed by ISG induction. Moreover, while saRNA platforms are known to induce transient innate immune activation in vivo, we are not aware of evidence showing harmful effects specifically attributed to enhanced antigen production per se. We agree that this is an important consideration in translational optimisation and have amended the discussion accordingly.

Lines 779 to 785 now read:

"The cis-expression of NoV B2 from saRNA may therefore not only enhance antigen expression but also preserve cell viability by reducing this global translational shutdown. While prolonged cell viability and sustained transgene expression could, in principle, influence immune activation kinetics in vivo, such effects are likely to be context- and dose-dependent. Importantly, we did not observe increased ISG induction upon B2-mediated enhancement of transgene expression under our experimental conditions, suggesting that enhanced saRNA expression does not exacerbate IFN-I response."

Minor comments

1. *Line 134: suggestion to clarify in the sentence that this refers to sa-RNA vectors by changing the sentence in 'CHIKV and VEEV-based sa-RNA vectors'.*

This has been corrected as suggested.

2. *Line 142 and 621: suggestion to change 'replicon vector' in 'sa-RNA vector' to keep the text consistent and clear.*

Corrected as suggested.

3. *Line 247 and 248: replace comma by full stop after 'duplicates' and 'Switzerland'.*

Corrected as suggested.

4. *Line 358: the authors use mainly 'sa-RNA' but a couple of times 'saRNA'. For consistency, please keep to one abbreviation.*

This has now been corrected and the abbreviation "saRNA" is now used throughout the manuscript.

5. *Figure S1: In panel B the names of the vectors are different from the names in Figure 1 and the figure description. It would be recommended to adapt the name in Figure S1B. Furthermore, I was wondering what is meant with the vehicle in panel B. Can the authors explain this in the figure description.*

We have now adapted the names of the vectors in Figure S1B. We have also clarified the figure legend to specify that cells transfected with the vehicle correspond to cells transfected with transfection reagent alone.

6. *Figure 1: In panel A the schematic representation of the CHIKV sa-RNA vectors is too small to fully understand the design of the vectors. In panel B the x-axis description is missing.*

We increased the size of the schematic representation of the vectors in Fig.1A and added the x-axis description in Fig.1B.

7. *Line 737: I think that 'HeLa cells' should be 'mESCs'?*

We corrected this mistake.

8. *Line 745: the figure description states '21- and 23-nt reads', but in the text it states (line 381) '21-23-nt viral reads'. I assume that the figure indeed shows the 21- to 23-nt reads? If so, please adapt this in the figure description.*

We revised and mentioned "21-23-nt reads" in the main text.

9. Line 505: in this case sa-RNA-GFP means CHIKV-GFP sa-RNA?

It indeed corresponds to CHIKV-GFP saRNA and we have now amended the text.

10. Figure 5: there is some inconsistency in the names in the figure, e.g. 'STrEP B2 – wt' in A vs 'STrEP B2WT'in D, and 'αNSP2' in E vs 'αNsp2' in F (in text described as nsP2).

The nomenclature has been standardised throughout figure 5 using "STrEP $B2^{WT}$" in panel A and D and adopted "nsP2" consistently across all panels.

11. Line 854: M should be N. Description of M is missing.

"M" has been corrected to "N" and the description of Figure 6M has now been added in the revised manuscript.

Reviewer #3 (Remarks to the Author):

This manuscript investigates whether expression of the Nodamura virus (NoV) B2 protein can improve self-amplifying RNA (sa-RNA) expression by counteracting host antiviral defenses. The authors conclude that B2 suppresses RNAi in embryonic stem cells and alleviates PKR-mediated translational shutdown in somatic cells, thereby enhancing reporter or antigen expression from alphavirus replicons while maintaining interferon induction. They further suggest that this strategy could inform vaccine development. While the study is technically competent, several issues limit its conceptual impact and overall significance.

Major Concerns

1. Lack of novelty and overstated claims. The central observation—that a dsRNA-binding viral suppressor of RNA silencing can improve replicon expression by blunting host defenses—is predictable. Viral proteins such as vaccinia E3L and influenza NS1 have long been known to block PKR and other sensors through dsRNA sequestration. That NoV B2 would exert a similar effect on synthetic alphavirus replicons is not a surprising advance. The claims of conceptual novelty and translational relevance are overstated.

We thank the reviewer for this comment and would like to address the concern about predictability with both published evidence and our own experimental observations. While VACV-E3L and Influenza NS1 have been shown to antagonise PKR in the context of viral infection, the outcome of applying such strategies to alphavirus replicating RNA systems cannot be assumed to be predictable. Indeed, our own experience illustrates this point. Encouraged by our findings with NoV B2 in alphavirus saRNA, we tested whether a similar approach would improve performance of trans-amplifying RNAs (taRNAs), a related but distinct alphavirus-based RNA replication system[7]. Strikingly, rather than improving expression, cis-expression of NoV B2 markedly reduced expression across multiple taRNA systems, suggesting that the effect of dsRNA-binding proteins on alphavirus RNA replication platforms is highly context-dependent and cannot be predicted a priori, While a full investigation of this observation is beyond the scope of the current manuscript, it demonstrates that the outcome of such interventions cannot be assumed.

We agree that certain viral dsRNA-binding proteins such as vaccinia E3L and Influenza NS1 have been shown to antagonise PKR and related antiviral sensors in the context of a viral infection. However, our data and previous studies indicate that dsRNA binding alone is not sufficient to enhance the efficiency of self-amplifying RNA (saRNAs) or to evade innate immune activation and therefore cannot be assumed to predict replicon efficacy. Consistent with this, we found that encoding other known dsRNA-binding proteins in *cis*, such as human enterovirus 71 3A and vaccinia E3L, either failed to increase or

only modestly increased transgene expression via partial alleviation of PKR restriction (Figure 4J). Similarly, NS1 encoded in *cis* was previously shown to be unable to boost saRNA gene expression[8]
In contrast, our study identifies NoV B2 as viral antagonist that effectively enhances saRNA-mediated gene expression and provides mechanistic insights into how this occurs. Beyond dsRNA binding, we show that NoV B2 alleviates PKR-mediated restriction, a central bottleneck for saRNA expression in somatic cells. While dsRNA binding is required for this effect, we further observe that NoV B2 limits the redistribution of dsRNA within the cell, leading to its retention at the cell periphery. We further show that NoV B2 localises to the cell periphery and associates with a component of the viral replication complex (i.e.nsP2), supporting a model in which NoV B2 binds both dsRNA and the saRNA replication complex, thereby stabilising dsRNA at the peripheral replication sites. To our knowledge, this spatial mechanism of dsRNA sequestration has not been previously been described in the context of engineered saRNA platforms. Importantly, NoV B2 does not impair interferon-stimulated gene induction, thereby preserving the intrinsic immunostimulatory properties of saRNA. This provides a strategy to enhance antigen expression without compromising type I interferon signalling and associated self-adjuvanticity, a balance that remains a key challenge in current saRNA optimisation approaches.

2. Unclear biological relevance. The physiological role of B2 in mammalian immunity is not convincingly established. Nodamura virus has at best a limited ability to replicate in mammals, raising doubts that its interactions with PKR or RNAi pathways reflect an evolved immune antagonism rather than experimental artifact. Without stronger evidence that NoV naturally manipulates mammalian antiviral defenses, the significance of B2 as a "tool" for vaccine design remains questionable.

While Nodamuravirus (NoV) is most studied in invertebrate systems, there is substantial genetic evidence demonstrating that NoV B2 actively antagonise innate immune pathway (e.g. antiviral RNA interference) in both mammalian cells and in vivo. B2-deficient NoV (NoV delta B2) is markedly attenuated in mouse embryonic stem cells and its accumulation is rescued in cells lacking Argonaute proteins[9] . NoV delta B2 is also attenuated in suckling mice and induces accumulation of viral small interfering RNAs in vivo[10] . More recently, NoV infection of adult mice was shown to trigger Dicer-dependent antiviral RNAi with B2 suppressing this response to promote viral replication [11,12]. Together these loss-of-function and in vivo studies indicate that NoV B2 activity in mammalian system reflects functional antagonism of host antiviral pathways.
Importantly, our study does not rely on whether NoV B2 evolved specifically to counteract mammalian immunity. We show experimentally and unequivocally that NoV B2 alleviates PKR-mediated restriction and Dicer-dependent RNAi in mammalian cells in the context of saRNA. The utility of viral immune antagonists as molecular tools does not depend on strict host adaptation, but rather on their mechanistic capacity to modulate conserved innate immune pathways.

3. Insufficient mechanistic insight. The manuscript does not provide rigorous mechanistic explanations for many of the observed effects. The RNAi-related experiments in embryonic stem cells are inconclusive and do not demonstrate a functional antiviral role for Dicer. In somatic cells, the data argue against antiviral RNAi altogether.

Previous studies have demonstrated an antiviral role for RNAi in mouse embryonic stem cells and tissue stem cells, which are characterised by an attenuated type I IFN response[9,13,14]. Upon infection with RNA viruses, these cells mount an antiviral RNAi response marked by the accumulation of viral-derived small interfering RNAs (siRNAs) of 21-23 nucleotides in length. In contrast, this response is reduced, although not completely abolished upon differentiation of mESCs, indicating that Dicer-mediated processing of long dsRNA is restrained in differentiated cells[9] . This has been linked to an antagonistic

relationship between the type I IFN system and RNAi, whereby RNAi activity is suppressed in somatic cells in which the IFN response is fully active[15–17] . In addition, a recently identified isoform of Dicer, termed antiviral Dicer (aviD), exhibits enhanced dsRNA cleavage activity and is selectively expressed in stem cells supporting a specialised role for RNAi in these cells[13] .

Within this established framework, the present study examines how the viral suppressor of RNAi (VSR) NoV B2 modulates saRNA biology. We find that expression of NoV B2 in *cis* from a CHIKV-GFP saRNA enhances GFP expression in mESCs, a cellular context in which antiviral RNAi is active. Concomitantly, transfection of CHIKV-GFP-B2^{R59Q} leads to the accumulation of 21-23 nt small RNA mapping to both strands of the viral genome with enrichment at the 5'end of the saRNA, consistent with Dicer-derived product. In contrast, expression of NoV B2^{WT} results in a marked reduction of these viral-derived small RNAs together with increased GFP expression from the saRNA.

These observations are consistent with the well-documented activity of NoV B2 as a dsRNA-binding protein that inhibits Dicer-mediated processing, thereby limiting the accumulation of viral-derived siRNAs[9,10,18,19] .The reciprocal effects of NoV B2^{WT} and the dsRNA-binding mutant (R59Q) are in strong agreement with previously described mechanisms of RNAi suppression by B2. In somatic cells, our data do not support a detectable contribution of antiviral RNAi to saRNA-mediated GFP expression, consistent with previous studies indicating that this pathway is restrained in differentiated cells[15,17,20–22] . Instead, our data indicate that saRNA restriction in somatic cells is primarily mediated by PKR, a central dsRNA-dependent antiviral effector.

4. The dsRNA strand analysis is interpreted inconsistently—if B2 protected replication intermediates by binding dsRNA, both positive and negative strands should be stabilized, not just the positive strand. More direct assays (e.g., RNA immunoprecipitation) would be required to demonstrate B2 binding to replicon dsRNA.

The reviewer suggests that if NoV B2 protected replication intermediates by binding dsRNA, both positive (+) and negative (–) strands should be similarly stabilised. In our data, however, NoV B2 preferentially enhances (+)-sense genomic and subgenomic RNA, with little impact on (–)-sense genomic RNA (RT-qPCR from bulk or sorted cells; see Figure 2H, revised Fig.S2B and S2C and response to Reviewer 2, comment 5).

We interpret these observations in the context of the temporally ordered nature of alphavirus replication. Translation of the incoming (+)-sense RNA produces the nsP1–4 polyprotein, which forms the early replication complex responsible for (–)-strand synthesis. Only after proteolytic processing does the complex switch to robust (+)-sense genomic and subgenomic RNA production. As NoV B2 is expressed from the subgenomic RNA, it accumulates during this later phase of replication, after the majority of (–)-strand synthesis has occurred. Consequently, NoV B2 would not be expected to substantially influence initial (–)-strand generation but rather to act during the amplification of (+)-sense RNA.

Consistent with this interpretation, strand-specific RT-qPCR performed on FACS-sorted GFP-positive cells reproduced the preferential increase in (+)-strand RNA (see Fig. S2C added as panel A below for this rebuttal). In parallel, dsRNA staining confirmed that dsRNA replication intermediates are still generated in HeLa WT cells in which saRNA encoding NoV B2^{R59Q} mutant remains restricted (panel B). Furthermore, quantification of dsRNA foci in HeLa *PKR*$^{-/-}$ cells transfected with CHIKV-GFP-B2^{WT} or CHIKV-GFP-B2^{R59Q} did not reveal a significant difference between conditions (panel C). Together, these observations do not support a model in which NoV B2 globally stabilises dsRNA replication intermediates.

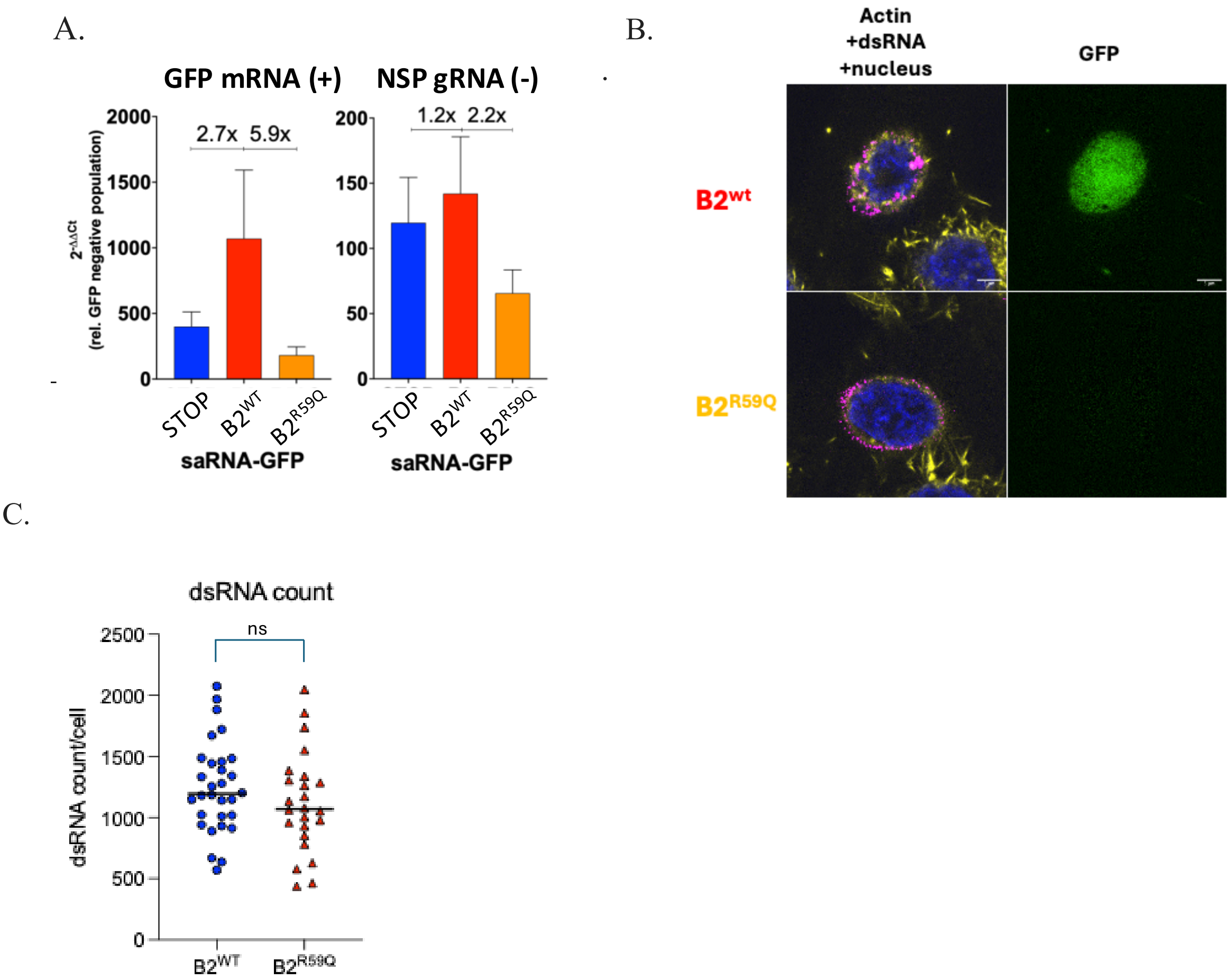

(A) HeLa cells were transfected with CHIKV-GFP-STOP (blue), CHIKV-GFP-$B2^{WT}$ (red) or CHIKV-GFP-$B2^{R59Q}$ (yellow) expressing saRNA. 24 hours post-transfection, GFP^{high} cells were isolated by FACS prior to RNA extraction and strand-specific RT-qPCR analysis to assess the levels of positive (+)- strand GFP mRNA (left) or CHIKV negative (-)-strand RNA levels using primers targeting nsP1 region (right). (B) Representative image of HeLa WT, transfected with CHIK-GFP- STrEP-$B2^{WT}$ or -STrEP-$B2^{R59Q}$ saRNA for 20 hours. Cells were stained for dsRNA with clonal J2 antibody (magenta), F-actin (yellow) and nuclei (blue). GFP fluorescence (green) indicates saRNA expression. Scale Bars: 5 µm. (C) dsRNA was quantified using confocal images of HeLa $PKR^{-/-}$ cells transfected with CHIKV-GFP-$B2^{WT}$ (blue, n=31 cells) or CHIKV-GFP-$B2^{R59Q}$ (red, n=26 cells). Each dot represents a single cell.

To directly address the reviewer's request for evidence of dsRNA binding, we performed immunoprecipitation experiments. As the saRNA expressing NoV B2 mutant is restricted in PKR-proficient cells, these experiments were conducted in HeLa *PKR* $^{-/-}$ cells. Cells were transfected with CHIKV saRNA encoding N-terminally STrEP-tagged NoV $B2^{WT}$ (CHIKV-GFP-STrEP-B2) or the dsRNA-binding-negative mutant NoV $B2^{R59Q}$ (CHIKV-GFP-STrEP-$B2^{R59Q}$) followed by STrEP-mediated immunoprecipitation 24 hours post-transfection. dsRNA was measured by dot blot analysis of total and co-precipitated RNA. Robust dsRNA signals were detected in total lysates from saRNA-transfected cells (see below). Consistent with our previous observations, comparable levels of dsRNA were present in cells expressing NoV $B2^{WT}$ compared to NoV $B2^{R59Q}$ indicating that NoV B2 does not increase overall dsRNA abundance. We validated that dsRNA was efficiently co-precipitated with NoV $B2^{WT}$ but not with NoV $B2^{R59Q}$ mutant, demonstrating that dsRNA binding is retained in this context. These findings are consistent with prior biochemical studies showing that NoV B2 binds dsRNA[19,23,24] and confirm that this activity is retained in the context of saRNA. We have included these new data in

Figure 5E (see below) and have updated the main text (lines 596-600), the Materials and Methods (lines 348-364) and the figure legend accordingly.

Figure 5E:

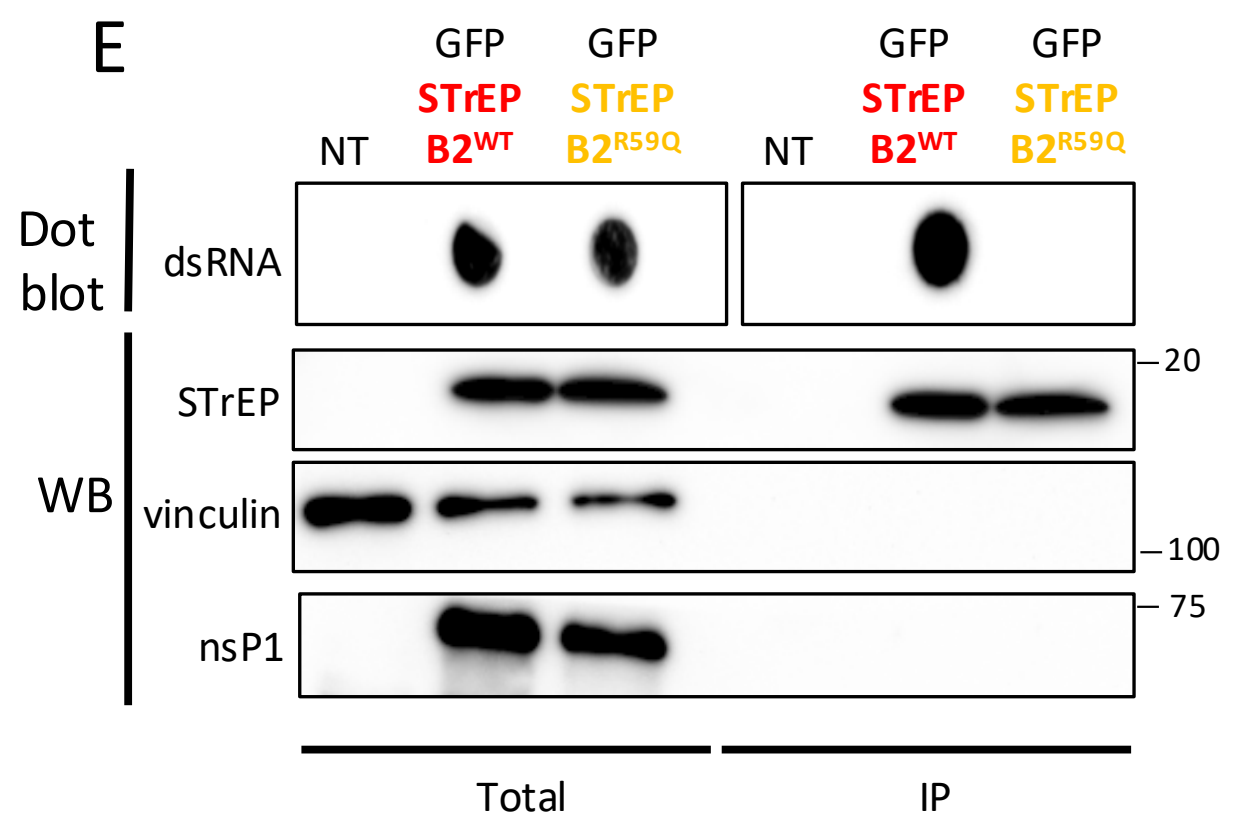

(E) HeLa *PKR*$^{-/-}$ cells were transfected with CHIKV saRNA encoding GFP-STrEP-B2^{WT} or -B2^{R59Q} saRNA. At 24 hours post-transfection STrEP-mediated immunoprecipitation was performed followed by dot blot analysis and western blotting. dsRNA levels were assessed by dot blot analysis in total lysate (input) and immunoprecipitated fractions (IP) (top panels). Levels of STrEP-tagged B2 proteins and nsP2 were assessed in total and IP fractions by western blotting (WB, bottom panels). Vinculin served as a loading control.

5. Overstated translational impact. The suggestion that co-opting B2 provides a viable strategy for sa-RNA vaccine improvement is premature. Introducing a viral immune evasion protein into vaccine platforms raises safety and immunogenicity concerns that are not addressed. The limited interferon data do not exclude broader effects on innate signaling, and the potential for dampening protective immunity remains unresolved.

We agree that the translational implications of our findings should be framed more carefully and we have revised the discussion accordingly to make clear that our study reveals a novel mechanistic strategy that we believe will inspire the development of next-generation saRNA and should not be considered a clinically ready strategy.
Regarding the specific concern that NoV B2 may dampen protective immunity through broader suppressing of innate immune signalling, we respectfully note that our data already address this point to a significant extent. We show that NoV B2 expression from CHIKV and VEEV saRNAs does not alter the expression of multiples interferon-stimulated genes (ISGs) (i.e IFIT1, IFIT2, RIG-I, CCL5) (Fig.2I, S2D, 6N) nor induction of type I IFN (IFN-I) as measured by the IFIT2 promoter-driven luciferase reporter in THP1-2R cells (Fig. 3D, 6G). Importantly, in somatic cells NoV B2 enhances saRNA efficacy by alleviating PKR-mediated translational shutdown rather than broadly suppressing innate immune signalling. These findings are supported by multiple published studies. Li *et al.*[10] performed a comprehensive quantitative RT-qPCR analysis of 84 key genes from known innate antiviral pathways in suckling mice and detected no differences in innate immune gene expression between infection with wild-type NoV, which encodes B2, and a B2-deficient mutant. Fan *et al.*[25] , who demonstrated that NoV infection of mouse embryonic fibroblasts (MEFs), despite the presence of B2 protein, still induced a robust RIG-I-dependent IFN-β and ISG response. Consistent with this, Han *et al.*[11] showed directly that the *cis*-expression of NoV B2 from replicating NoV RNA had no detectable effect on the induction of ISG15 and RIG-I in MEFs and similarly had no inhibitory effect on IFN-β, RIG-I and ISG15 induction in adult mice. We fully agree however that a comprehensive *in vivo* analysis of immunogenicity and safety remains an important focus of future work and we have revised the discussion to reflect this.

6. Presentation and rationale. Experimental rationale is often missing. The choice of cell lines is not explained, and some assays (e.g., co-immunoprecipitations, eIF2α phosphorylation) are performed

only in specific contexts without justification. Figures are numerous and descriptive, but not all are necessary; the Introduction and Discussion reiterate background material rather than providing synthesis or perspective.

We agree that the rationale for certain experimental systems and approaches was not always stated explicitly and we have revised the manuscript to clarify the purpose and justification of key experiments. Specifically, we have added rationale statements for: (i) the choice of HeLa, THP-1 and HEK293T cell lines (lines 422-424, 464-468 and 482-484, respectively); (ii) the co-immunoprecipitation experiments (lines 602-611); (iii) the eIF2α phosphorylation analysis (lines 535-539).

To address this point in more details, we have added brief explanatory statements throughout the Results section to clarify the choice of cell lines and assays at the point where they are introduced. For example, we now explicitly state the rationale for the use of THP-1 cells as an immune-relevant model, HEK293T for biochemical analyses and to dissect pathway-specific contributions due to their transfectability and availability of knockout lines and HeLa cells, a widely used somatic cell line.

Regarding the co-immunoprecipitation experiments, these were motivated by our microscopy observations and by compelling structural parallels between the replication complexes of Nodaviridae and alphaviruses. By confocal microscopy, NoV B2 was frequently observed in proximity to nsP1 and dsRNA although clear co-localisation was not detected. In contrast, nsP2 staining showed partial overlap with NoV B2 (Figure 5A, 5B). Structural analyses of the CHIKV replication complex indicate that nsP2 is docked above the membrane-anchored nsP1 dodecameric ring facing the cytoplasm and adjacent to nspP1 within the replication core [26], placing it in a spatial position accessible to cytoplasmic factors such as B2. These microscopy observations therefore raised the possibility that NoV B2 might interact with components of the alphavirus replication complexes. This hypothesis was further supported by striking structural and functional parallels between nodavirus and alphavirus replication complexes. Both NoV Protein A and alphavirus nsP1 multimerise into 12-mer ring-like structures anchored at cellular membranes and share significant homology in their MTase/GTase domains[27,28] Importantly, the B2 protein from FHV, another member of the Nodaviridae family closely related to NoV, has previously been shown to co-immunoprecipitate with Protein A, the viral RNA-dependent RNA polymerase, in FHV-infected insect cells supporting an association between nodaviral B2 proteins and the viral replication machinery[18,29] . Given these structural parallels between nodavirus and alphavirus replication complexes and the known interaction between FHV B2 and protein A, we hypothesised that NoV B2 might similarly associate with components of the alphavirus replication complex when expressed from CHIKV saRNAs. We therefore co-expressed STrEP-tagged $B2^{wt}$ or $B2^{R59Q}$ with either a combination of expression plasmids encoding for CHIKV replicase proteins (nsP1+nsP2+nsP3+nsP4) or a plasmid encoding nsP2 alone followed by STrEP immunoprecipitation and western blotting analysis for nsP1 and nsP2. NoV B2 specifically co-immunoprecipitated with nsP2 confirming that NoV B2 associates with the alphavirus replication machinery. This interaction was dependent on both B2's dsRNA binding activity and the MTase-like domain of nsP2, which is distally located from spherule, faces cytosol and is exposed enough to be able to functionally interact with different proteins, including host factors of CHIKV replication[30] . This rationale has now been clarified in the Results section (lines 600-617).

For the analysis of eIF2α phosphorylation, the rationale stems from the observation that NoV B2 alleviates PKR-mediated restriction of saRNAs (Figure 4A-E). PKR activation is known to exert its antiviral effects primarily through phosphorylation of eIF2α, resulting in translational shutdown. We therefore assessed eIF2α phosphorylation to determine whether NoV B2 counteracts this PKR-dependent pathway, testing the mechanistic hypothesis that NoV B2 enhances saRNA activity by

preventing PKR-mediated translational inhibition. This explanation has now been added to the Results section (lines 533-537).

Finally, we have revised the Introduction and Discussion to remove redundant background material and improve focus. We have consolidated and streamlined the figure panels where possible to improve clarity.

7. In conclusion, the study shows that NoV B2 can enhance expression from alphavirus saRNAs by interfering with host defenses, but this is an expected outcome given prior knowledge of viral dsRNA-binding proteins. The mechanistic evidence is incomplete, the biological relevance in mammalian systems is uncertain, and the translational claims are overstated. While the experiments are competently executed, the work does not provide the level of conceptual advance or physiological insight required for in a high-impact journal.

We believe that the additional experiments and clarifications in response to all reviewers' comments provide clearer mechanistic insights and strengthen our findings.

References:

1. Kennedy, E. M. *et al.* Production of functional small interfering RNAs by an amino-terminal deletion mutant of human Dicer. *Proceedings of the National Academy of Sciences* **112**, E6945-54 (2015).

2. Akhrymuk, I., Lukash, T., Frolov, I. & Frolova, E. I. Novel Mutations in nsP2 Abolish Chikungunya Virus-Induced Transcriptional Shutoff and Make the Virus Less Cytopathic without Affecting Its Replication Rates. *J Virol* **93**, (2019).

3. Thaa, B. *et al.* Differential Phosphatidylinositol-3-Kinase-Akt-mTOR Activation by Semliki Forest and Chikungunya Viruses Is Dependent on nsP3 and Connected to Replication Complex Internalization. *J. Virol.* **89**, 11420–11437 (2015).

4. Omler, A., Rutmane, A., Mahalingam, S. & Merits, A. The ability of alphavirus replicases to synthesize non-viral type I interferon-inducing RNAs correlates with viral RNA synthesis and has a diverse impact on virus replication and pathogenicity. *J. Virol.* **100**, e02162-25 (2026).

5. Kujala, P. *et al.* Biogenesis of the Semliki Forest Virus RNA Replication Complex. *J. Virol.* **75**, 3873–3884 (2001).

6. Frolova, E. I., Gorchakov, R., Pereboeva, L., Atasheva, S. & Frolov, I. Functional Sindbis Virus Replicative Complexes Are Formed at the Plasma Membrane. *J. Virol.* **84**, 11679–11695 (2010).

7. Beissert, T. *et al.* A Trans-amplifying RNA Vaccine Strategy for Induction of Potent Protective Immunity. *Mol. Ther.* **28**, 119–128 (2020).

8. Blakney, A. K. *et al.* Innate Inhibiting Proteins Enhance Expression and Immunogenicity of Self-Amplifying RNA. *Mol Ther* **29**, 1174–1185 (2021).

9. Maillard, P. V. *et al.* Antiviral RNA interference in mammalian cells. *Science (New York, N.Y.)* **342**, 235–238 (2013).

10. Li, Y., Lu, J., Han, Y., Fan, X. & Ding, S.-W. RNA Interference Functions as an Antiviral Immunity Mechanism in Mammals. *Science* **342**, 231–234 (2013).

11. Han, Q. *et al.* Mechanism and Function of Antiviral RNA Interference in Mice. *mBio* **11**, 855–18 (2020).

12. Zhang, Y. *et al.* Efficient Dicer processing of virus-derived double-stranded RNAs and its modulation by RIG-I-like receptor LGP2. *Plos Pathog* **17**, e1009790 (2021).

13. Poirier, E. Z. *et al.* An isoform of Dicer protects mammalian stem cells against multiple RNA viruses. *Science* **373**, 231–236 (2021).

14. Xu, Y.-P. *et al.* Zika virus infection induces RNAi-mediated antiviral immunity in human neural progenitors and brain organoids. *Cell research* **46**, 509 (2019).

15. Maillard, P. V. *et al.* Inactivation of the type I interferon pathway reveals long double-stranded RNA-mediated RNA interference in mammalian cells. *The EMBO journal* **35**, 2505–2518 (2016).

16. Veen, A. G. van der *et al.* The RIG-I-like receptor LGP2 inhibits Dicer-dependent processing of long double-stranded RNA and blocks RNA interference in mammalian cells. *Embo J* **37**, e97479 (2018).

17. Maillard, P. V., Veen, A. G., Poirier, E. Z. & Sousa, C. R. e. Slicing and dicing viruses: antiviral RNA interference in mammals. *Embo J* **38**, e100941 (2019).

18. Aliyari, R. *et al.* Mechanism of induction and suppression of antiviral immunity directed by virus-derived small RNAs in Drosophila. *Cell host & microbe* **4**, 387–397 (2008).

19. Sullivan, C. S. & Ganem, D. A virus-encoded inhibitor that blocks RNA interference in mammalian cells. *Journal of virology* **79**, 7371–7379 (2005).

20. Veen, A. G. V. der *et al.* The RIG-I-like receptor LGP2 inhibits Dicer-dependent processing of long double-stranded RNA and blocks RNA interference in mammalian cells. *The EMBO journal* **37**, e97479 (2018).

21. Seo, G. J. *et al.* Reciprocal inhibition between intracellular antiviral signaling and the RNAi machinery in mammalian cells. *Cell host & microbe* **14**, 435–445 (2013).

22. Uhl, S. *et al.* ADAR1 Biology Can Hinder Effective Antiviral RNA Interference. *J. Virol.* **97**, e00245-23 (2023).

23. Körber, S., Ali, P. S. S. & Chen, J. C.-H. Structure of the RNA-Binding Domain of Nodamura Virus Protein B2, a Suppressor of RNA Interference,. *Biochemistry* **48**, 2307–2309 (2009).

24. Chao, J. A. *et al.* Dual modes of RNA-silencing suppression by Flock House virus protein B2. *Nature structural & molecular biology* **12**, 952–957 (2005).

25. Fan, X., Dong, S., Li, Y., Ding, S. W. & Wang, M. RIG-I-dependent antiviral immunity is effective against an RNA virus encoding a potent suppressor of RNAi. *Biochemical and biophysical research communications* 1–6 (2015) doi:10.1016/j.bbrc.2015.03.145.

26. Tan, Y. B. *et al.* Molecular architecture of the Chikungunya virus replication complex. *Sci. Adv.* **8**, eadd2536 (2022).

27. Ahola, T. & Karlin, D. G. Sequence analysis reveals a conserved extension in the capping enzyme of the alphavirus supergroup, and a homologous domain in nodaviruses. *Biol. Direct* **10**, 16 (2015).

28. Boon, J. A. den, Nishikiori, M., Zhan, H. & Ahlquist, P. Positive-strand RNA virus genome replication organelles: structure, assembly, control. *Trends Genet.* **40**, 681–693 (2024).

29. Boon, J. A. den *et al.* Multifunctional Protein A Is the Only Viral Protein Required for Nodavirus RNA Replication Crown Formation. *Viruses* **14**, 2711 (2022).

30. Wang, S. & Merits, A. G3BP/Rin-Binding Motifs Inserted into Flexible Regions of nsP2 Support RNA Replication of Chikungunya Virus. *J. Virol.* **96**, e01278-22 (2022).

31. Bogerd, H. P., Whisnant, A. W., Kennedy, E. M., Flores, O. & Cullen, B. R. Derivation and characterization of Dicer- and microRNA-deficient human cells. *Rna* **20**, 923–937 (2014).

We thank the editor and reviewers for their careful evaluation of our manuscript and for their constructive feedback, which has helped us strengthen the manuscript further. Our point-by-point responses are provided below.

REVIEWERS' COMMENTS

Reviewer #1 (Remarks to the Author):

The authors have satisfactorily addressed all my questions.

We are pleased that our revisions have satisfactorily addressed their concerns.

Reviewer #2 (Remarks to the Author):

Thank you for your thorough and careful response to the review comments. The authors have addressed all my concerns in a convincing and systemic manner. I appreciate the inclusion of an additional cell line in the transfection experiment, as well as the optimization of the transfection conditions, both enhanced the reliability of the results. Furthermore, the additional experiment in which RNA measurement was performed on GFP-sorted cells strengthens the evidence for the effect of B2 on the saRNA replication. The revised immunofluorescence analyses are clear and the additional immunofluorescence data provide a clearer understanding of the saRNA distribution pattern. I also appreciate the author's insightful discussion on the potential mechanism of B2 localization, dsRNA distribution and PKR restriction. Overall, the additional results and refined figures strengthen the manuscript and increase confidence in the conclusions. I have no further comments and recommend this manuscript for acceptance.

We appreciate the reviewer's constructive assessment and their recognition that the additional cell line, transfection optimisation, GFP-sorted RNA measurements and refined immunofluorescence analyses have substantiated our findings and clarified the mechanistic insights regarding B2 localisation and PKR restriction.

Reviewer #3 (Remarks to the Author):

The revised manuscript is substantially improved and the authors have addressed many of the major concerns raised in the initial review. In particular, they have added important new controls and experiments showing that the B2-dependent enhancement of saRNA expression is reproducible under high-transfection conditions in BHK-21 cells, is observed after VRP-mediated delivery, and extends to additional alphavirus backbones. They also now provide sorted-cell RT-qPCR data to address whether increased RNA levels simply reflect differences in transfection efficiency, improved PKR/eIF2α analyses, and new biochemical evidence that STrEP-tagged B2WT, but not B2R59Q, co-precipitates dsRNA in saRNA-transfected cells. These additions substantially strengthen the central claim that NoV B2 enhances saRNA-driven gene expression by counteracting PKR-dependent translational restriction in somatic cells while preserving measurable IFN/ISG induction. The authors have also improved the rationale for several experimental choices, including the use of THP-1 reporter cells, HEK293T knockout lines, HeLa PKR−/− cells, and BHK-21 cells. The revised microscopy and nsP2 co-immunoprecipitation data provide a more coherent model in which B2 binds dsRNA and associates with the alphavirus replication environment, potentially limiting cytoplasmic redistribution of dsRNA and reducing access to PKR-dependent translational control. This model is plausible and interesting, although still not fully proven.

We thank the reviewer for their positive evaluation of our revised manuscript. We address the remaining points below.

1. *Some concerns remain. First, the evidence for a functional antiviral RNAi restriction of saRNA in mESCs remains suggestive rather than definitive. The accumulation of 21–23 nt viral small RNAs and their reduction by B2WT are consistent with Dicer-mediated processing, but the manuscript still lacks direct genetic evidence that Dicer/Ago activity functionally suppresses saRNA expression in this system. The authors should therefore temper statements implying that antiviral RNAi is conclusively responsible for saRNA restriction in stem cells.*

We have now revised the abstract to read:

Lines 30 to 32: "In stem cells, NoV B2 limits the accumulation of Dicer-generated viral small RNAs, consistent with suppression of antiviral RNA interference, while in somatic cells, it prevents protein kinase R (PKR)-driven translational shutdown."

We have also added the following to the Discussion:

Lines 473 to 476: "However, we have not directly tested this using genetic depletion of Dicer or Ago2 and we cannot exclude a contribution from other B2-sensitive pathways. Genetic loss-of-function approaches will be needed to formally establish the functional contribution of antiviral RNAi in this context."

2. *Second, although the new dsRNA co-IP/dot blot experiment addresses an important previous gap, it does not identify the dsRNA species bound by B2 or prove that this interaction is the causal event preventing PKR-mediated eIF2α phosphorylation. The authors should present this as supportive evidence rather than as a complete mechanistic explanation.*

To address the reviewer's comment, we have added the following sentence to the Discussion, where we discuss the proposed model:

Lines 544 to 546: "Similarly, the precise identity of the dsRNA species bound by B2, and whether this interaction directly drives inhibition of PKR-mediated eIF2α phosphorylation, remain to be determined."

3. *Similarly, while the CHIKV nsP2 interaction is intriguing, the corresponding interaction with VEEV nsP2 was not tested, even though the mechanism is generalized to VEEV-based saRNA.*

We agree with the reviewer that we demonstrated the B2-nsP2 interaction specifically for CHIKV nsP2. We have revised the Discussion to state this explicitly:

Lines 540 to 543: "We note that the interaction between NoV B2 and nsP2 was demonstrated specifically for CHIKV nsP2. While NoV B2 similarly enhanced VEEV-based saRNA expression in a PKR-dependent manner and altered VEEV dsRNA distribution, we did not test whether NoV B2 interacts with VEEV nsP2, and whether this interaction generalises across alphavirus backbones remains to be established."

4. *Finally, the translational claims should remain cautious. The data support a potentially useful engineering principle for increasing saRNA expression, but all experiments remain cell-culture based. In vivo immunogenicity, safety, toxicity, antigen overexpression, effects on broader innate*

signaling, and possible immune responses against B2 remain unresolved. The revised Discussion acknowledges some of these issues, but the vaccine-development framing should be further softened.

We have revised the Discussion to keep our translational claims appropriately cautious. Following our discussion on the need to evaluate in vivo immunogenicity and toxicity, we have added:

Lines 566 to 572: "As a foreign viral protein, NoV B2 may itself be immunogenic in vivo. Whether anti-B2 immune responses develop upon vaccination and whether these affect antigen expression kinetics or limit repeat dosing remains to be determined. Our characterisation of innate immune activation was also limited to a defined panel of ISGs and cytokines in vitro. Broader effects of NoV B2 on innate immune signalling in vivo, as well as any consequences of sustained antigen expression, remain to be evaluated."

We have also revised the closing sentence of the Discussion to more precisely frame the translational implications of our findings as follows:

Lines 585 to 587: "Altogether, our findings define an engineering strategy to enhance saRNA-driven protein expression while preserving self-adjuvanticity, providing a foundation for future in vivo evaluation in vaccine and therapeutic contexts."

5. *Overall, the revised manuscript is much stronger and addresses most of the prior concerns. I would support publication after minor revisions focused on tempering the RNAi and translational claims and clarifying the remaining mechanistic uncertainties.*

We thank the reviewer for their support of publication. Our responses to comments 1-4 above detail the revisions made to temper the RNAi-related and translational statements and clarify the remaining mechanistic uncertainties.
